# Members of a Large Retroposon Family Are Determinants of Post-Transcriptional Gene Expression in *Leishmania*

**DOI:** 10.1371/journal.ppat.0030136

**Published:** 2007-09-28

**Authors:** Frédéric Bringaud, Michaela Müller, Gustavo Coutinho Cerqueira, Martin Smith, Annie Rochette, Najib M. A El-Sayed, Barbara Papadopoulou, Elodie Ghedin

**Affiliations:** 1 Laboratoire de Génomique Fonctionnelle des Trypanosomatides, Université Victor Segalen Bordeaux 2, Bordeaux, France; 2 UMR-5234 CNRS, Bordeaux, France; 3 Infectious Diseases Research Center, Centre Hospitalier de l'Université Laval Research Center, Quebec, Canada; 4 Department of Medical Biology, Faculty of Medicine, Laval University, Quebec, Canada; 5 The Institute for Genomic Research, Rockville, Maryland, United States of America; 6 Departamento de Bioquimica e Imunologica, Universidade Federal de Minas Gerais, Minas Gerais, Brazil; 7 Department of Cell Biology and Molecular Genetics, The University of Maryland, College Park, Maryland, United States of America; 8 Center for Bioinformatics and Computational Biology, The University of Maryland, College Park, Maryland, United States of America; 9 Division of Infectious Diseases, Department of Medicine, University of Pittsburgh School of Medicine, Pittsburgh, Pennsylvania, United States of America; Seattle Biomedical Research Institute, United States of America

## Abstract

Trypanosomatids are unicellular protists that include the human pathogens Leishmania spp. (leishmaniasis), Trypanosoma brucei (sleeping sickness), and Trypanosoma cruzi (Chagas disease). Analysis of their recently completed genomes confirmed the presence of non–long-terminal repeat retrotransposons, also called retroposons. Using the 79-bp signature sequence common to all trypanosomatid retroposons as bait, we identified in the Leishmania major genome two new large families of small elements—LmSIDER1 (785 copies) and LmSIDER2 (1,073 copies)—that fulfill all the characteristics of extinct trypanosomatid retroposons. LmSIDERs are ∼70 times more abundant in L. major compared to T. brucei and are found almost exclusively within the 3′-untranslated regions (3′UTRs) of L. major mRNAs. We provide experimental evidence that LmSIDER2 act as mRNA instability elements and that LmSIDER2-containing mRNAs are generally expressed at lower levels compared to the non-LmSIDER2 mRNAs. The considerable expansion of LmSIDERs within 3′UTRs in an organism lacking transcriptional control and their role in regulating mRNA stability indicate that *Leishmania* have probably recycled these short retroposons to globally modulate the expression of a number of genes. To our knowledge, this is the first example in eukaryotes of the domestication and expansion of a family of mobile elements that have evolved to fulfill a critical cellular function.

## Author Summary

Transposable elements (TEs) are DNA sequences capable of moving from one chromosomal region to another. A considerable fraction of higher eukaryote genomes is comprised of TEs, as exemplified in human (over 40% of the genome) and maize (over 50% of the genome). There is now a growing body of evidence to suggest that TEs can be functionally important and not just “junk,” “selfish,” or “parasitic” DNA sequences that make as many copies of themselves as possible. Indeed, during the past ten years, a considerable number of TE copies have been described as domesticated or exapted elements playing a cellular function, such as transcriptional regulation and contribution to protein-coding regions. TE domestication has been described for only a few copies of TE families, and exaption of a whole TE family has not been reported so far. We provide evidence that Leishmania spp., unicellular protists responsible for human diseases, have recycled and expanded a whole family of short and extinct TEs (retroposons) that have evolved to fulfill an important biological pathway, i.e., regulation of gene expression. We also observed that Trypanosoma brucei (a close relative of Leishmania spp.) developed other approaches to maintain the same cellular function.

## Introduction

Trypanosomatids are members of the kinetoplastid family of unicellular protists, which includes human pathogens responsible for Chagas disease (Trypanosoma cruzi), African sleeping sickness (Trypanosoma brucei), and leishmaniasis (Leishmania spp.). T. brucei and T. cruzi belong to the monophyletic *Trypanosoma* group, which is distantly related to all the other trypanosomatids, including Leishmania spp. [1]. Kinetoplastid protein-coding genes are often organized as large directional gene clusters (DGCs) that form polycistronic units [2–4]. Individual mRNAs with a 39-nt 5′ capped spliced leader sequence and 3′ poly(A) tail are generated from the polycistronic pre-mRNAs via 5′ *trans*-splicing and 3′ cleavage-polyadenylation reactions [5]. Several lines of evidence raise the intriguing possibility that in trypanosomatids poly(A) addition is coupled to *trans*-splicing of the downstream gene [6,7]. *trans*-splicing signals are often U-rich polypyrimidine tracts, which precede AG acceptor sites on average 50–100 nt upstream of the translational start site. There is no consensus polyadenylation signal in trypanosomatid mRNA, and evidence obtained from a small number of loci suggests that polyadenylation occurs within a short region 100–400 nt upstream of the next polypyrimidine *trans*-splicing signal [7,8]. It was recently reported that in 89% of all available cDNA sequences from T. brucei, polyadenylation usually occurs at an A residue located between 80 and 300 nt from a downstream polypyrimidine tract [9]. The aforementioned polycistronic transcription, and the absence of pol II promoters in all known protein-coding genes, necessitate that gene expression be controlled post-transcriptionally. Indeed, numerous examples in kinetoplastids, including *Leishmania,* show that sequences predominantly located in the 3′-untranslated regions (3′UTRs) control mRNA stability and translation [10–18].

Transposable elements (TEs) are DNA sequences capable of moving from one chromosomal region to another. They are classified into two major groups based on the mechanisms used for their transposition. Class I TEs, or retroelements, transpose via reverse transcription of an RNA intermediate and are further divided into the long-terminal repeat (LTR) retrotransposons with LTRs and the non-LTR retrotransposons, also called retroposons. Class II TEs, or DNA transposons, move strictly through a DNA intermediate. A considerable fraction of higher eukaryote genomes comprises TEs, as exemplified in human (over 40% of the genome) [19] and maize (over 50% of the genome) [20]. There is now a growing body of evidence to suggest that TEs can be functionally important and not just “junk,” “selfish,” or “parasitic” DNA sequences that make as many copies of themselves as possible [21–23]. For example, there is a considerable number of domesticated TE copies that act as transcriptional regulatory elements or contribute to protein-coding regions of cellular genes (for review see [24–26]).

**Figure 1:**
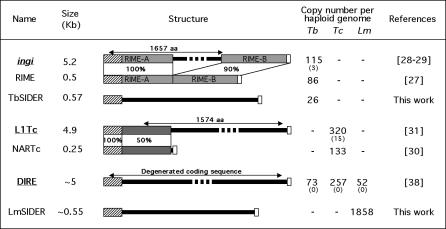
Description and Copy Number of the Trypanosomatid Retroposons Retroelement names and sizes are indicated in the left margin. The names of coding or potentially coding retroposons (including the few retrotransposition-competent *ingi* and L1Tc elements) are underlined and boldfaced; the other elements are short non-autonomous retroposons (RIME, NARTc, TbSIDER, and LmSIDER). The central panel (“Structure”) represents the schematic map of the retroelements highlighting nucleotide sequence conservation, such as for the *T. brucei ingi*/RIME and T. cruzi L1Tc/NARTc pairs. The grey boxes represent conserved sequences between autonomous and non-autonomous members of a pair (the percentage of identity is indicated below), the hatched boxes represent the 79-bp sequence conserved at the 5′-extremity (“79 bp signature”), and the white boxes indicate the adenosine-rich stretch terminal sequences. The right panel indicates the number of each retroelement per haploid genome, including minichromosomes for T. brucei (dashes indicate the absence of elements in the corresponding genome). For autonomous elements, the value in brackets indicates the number (per haploid genome) of potentially functional elements, which may code for their own retrotransposition. Lm, L. major; Tb, T. brucei; Tc, T. cruzi.

The recent completion of the Tritryp genome projects confirmed the presence of LTR retrotransposons and non-LTR retrotransposons (transposons) but no DNA transposons [2–4]. Retroposons constitute the most abundant TEs described in the genome of T. cruzi and T. brucei (∼3% of nuclear genome), while no potentially active TEs have been characterized to date in L. major [3]. The most abundant retroposons, *ingi* and ribosomal mobile element (RIME) in T. brucei [27–29] and L1Tc and NARTc in T. cruzi [30,31], are distributed across their respective genomes, although they do show a relative site specificity for insertion [32,33]. The T. brucei RIME (0.5 kb) appears as a truncated version of the *T. brucei ingi* (5.25 kb), in which the central 4.7 kb fragment has been deleted (Figure 1). Similarly, the T. cruzi NARTc (0.25 kb) element was derived from L1Tc (4.9 kb) by a 3′ deletion [30]. The potentially functional *ingi* and L1Tc each encode a large single multifunctional protein that is probably responsible for their retrotransposition and that of the short non-autonomous RIME and NARTc, respectively [32,33]. Consequently, *ingi*/RIME and L1Tc/NARTc are considered as pairs of retroposons, as previously described for the human long interspersed element 1 (LINE1)/Alu, the eel UnaL2/UnaSINE1, and the plant LINE/S1 pairs [34–37]. Until now, potentially active or short non-autonomous TEs have not been detected in the L. major genome [3,4]. However, the genome does contain degenerated retroelements (L. major degenerated *ingi*/L1Tc-related elements [LmDIREs]) corresponding to remnants of extinct *ingi*/L1Tc-like retroposons [38]. Interestingly, the *ingi*/RIME and L1Tc/NARTc pairs and DIREs share the first 78–79 nucleotides even though they are otherwise unrelated to each other [30,38] (Figure 1). This “79 bp signature,” therefore, constitutes the hallmark of trypanosomatid retroposons.

Using the “79 bp signature” for BLASTN searches, we identified in the L. major genome 1,858 short (∼550 bp), noncoding and degenerated retroposons that belong to two new large families of relatively conserved repetitive DNA elements (L. major short interspersed degenerated retroposon 1 [LmSIDER1] and LmSIDER2), which display all the hallmarks of trypanosomatid retroposons. LmSIDER1 and LmSIDER2 are predominantly located in the 3′UTR of L. major mRNAs and represent the most abundant TEs now characterized in trypanosomatid genomes. Considering that regulation of gene expression in *Leishmania* is mediated almost exclusively by sequences within 3′UTRs, we hypothesized that LmSIDERs may play a role in the regulation of gene expression. In the present study, we provide experimental evidence that members of the second retroposon subfamily in L. major, LmSIDER2, promote mRNA destabilization. We conclude that Leishmania spp., but not trypanosomes, have recycled and probably expanded an extinct family of short retroposons that participate in the maintenance of an essential cellular function, i.e., the regulation of gene expression.

## Results

### Characterization of Short Degenerated Retroposons in L. major and T. brucei Genomes

**Figure 2:**
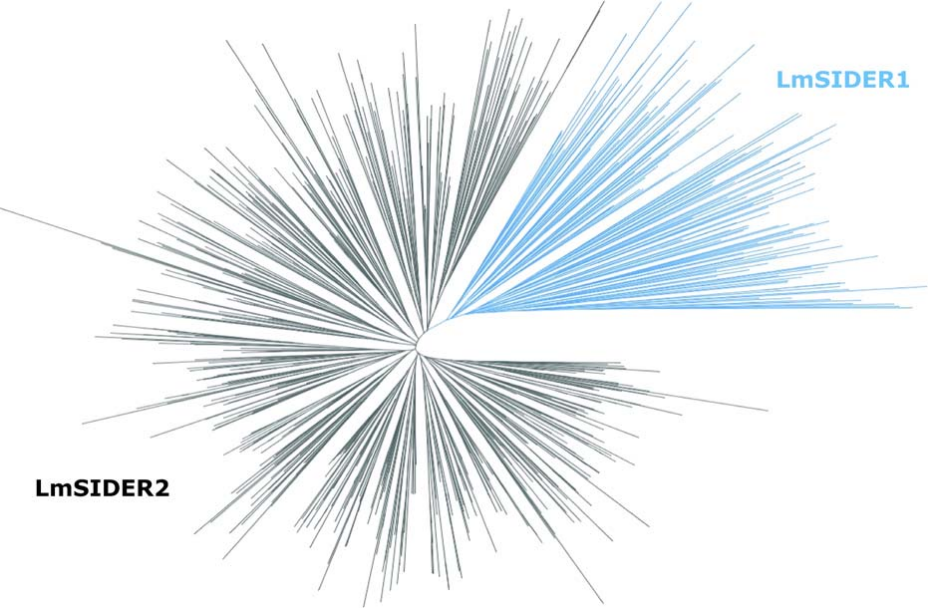
Minimum Evolution Phylogenetic Tree of 785 LmSIDER Sequences Only the LmSIDER sequences longer than 400 bp and smaller than 700 bp were considered to produce an alignment as described in Materials and Methods. The unrooted phylogenetic tree displays 140 LmSIDER1 (blue cluster) and 645 LmSIDER2 (black cluster) sequences.

All *ingi*/RIME, L1Tc/NARTc, and DIRE present in the T. brucei, T. cruzi, and L. major genomes have been identified and annotated [3]. These different retroposon families contain at their 5′-extremity a 79-bp conserved motif (called “79 bp signature”), which constitutes the hallmark of trypanosomatid retroposons [38]. In order to identify other repeated sequences containing the “79 bp signature,” we surveyed the L. major and T. brucei genomes for the presence of the first 79 bp of *ingi* and 78 bp of L1Tc. BLASTN searches initially detected 108 significant matches in the L. major genome, in addition to identifying the LmDIRE sequences. Comparison of the sequences located downstream of these 108 “79 bp signature” matches revealed two heterogeneous groups of sequences, which we named LmSIDER1 and LmSIDER2. After several rounds of BLASTN searches with complete LmSIDER1 and LmSIDER2 sequences, we identified 1,858 related sequences (785 LmSIDER1 and 1,073 LmSIDER2) in the L. major genome (Figure 1). Coordinates for these elements on each L. major chromosome are listed in Table S1. A phylogenetic analysis of 789 LmSIDER sequences confirmed their division into two distinct subfamilies, LmSIDER1 and LmSIDER2 (Figure 2). A similar BLASTN analysis of the T. brucei genome revealed 22 sequences forming two groups of relatively conserved sequences ranging from 558 to 587 bp, named T. brucei short interspersed degenerated retroposon 1 (TbSIDER1) (ten sequences) and TbSIDER2 (12 sequences) (Figure 1).

The work reported will hereafter primarily focus on the LmSIDER2 family. One thousand thirteen LmSIDER2s were aligned with the inclusion of numerous gaps to maximize the alignments (Figure S1). The aligned LmSIDER2 sequences ranged between 178 bp and 702 bp, with a mean of 545 bp. Although LmSIDER2 sequences are highly heterogeneous in composition and size (the alignment comprises 1,612 positions), a conserved core sequence was identified (538 bp) by removing insertions (1,074 positions) (Figure S2). The removed positions (66.6% of the positions in the original alignment) account for 14.5% of the aligned nucleotides. The defined core sequence was used to perform all the subsequent bioinformatics analyses.

**Figure 3:**
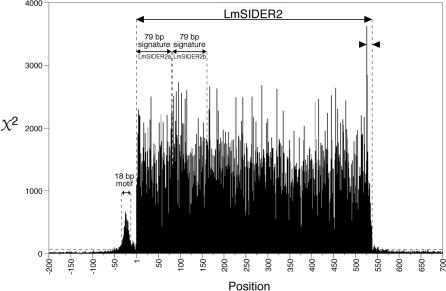
The *χ* ^2^ Values for Individual Positions of the LmSIDER2 Core Sequence (538 bp) and Adjacent Regions (200 bp Upstream and 160 bp Downstream) The *χ* ^2^ values were calculated as described in Materials and Methods from the set of 1,013 aligned LmSIDER2. The base composition of the whole L. major genome sequence was used to determine the background base distribution. The *χ* ^2^ values above the broken horizontal line correspond to significance levels of *p* < 0.001 for three degrees of freedom. Both “79 bp signatures,” called LmSIDER2a and LmSIDER2b, are positioned. The adenosine-rich stretch and the 18-bp thymidine-rich motif located at the 3′-extremity and upstream of the LmSIDER2, respectively, are indicated between arrowheads.

To determine whether the LmSIDER2 sequences are significantly conserved, we performed a chi-square (*χ* ^2^) test on the LmSIDER2 core and the flanking sequences (200 bp upstream and 160 bp downstream) (Figure 3). All positions of the LmSIDER2 core show a *χ* ^2^ score far above the threshold line corresponding to significant levels (using three degrees of freedom, a *χ* ^2^ value of 16.3 corresponds to a significance level of *p* < 0.001), indicating that the LmSIDER2 core is conserved. The flanking regions are not conserved, except for a thymidine-rich stretch (18 residues) starting at 15 bp upstream from the LmSIDER2 (unpublished data).

### LmSIDER and TbSIDER Sequences Contain All Hallmarks of Trypanosomatid Retroposons

**Figure 4:**
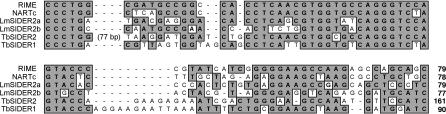
Comparison of the “79 bp Signature” Consensus Sequences between Different Trypanosomatid Retroposons The first 79 bp of RIME, 78 bp of NARTc, 161 bp of TbSIDER2, 90 bp of TbSIDER1, and both “79 bp signatures” located at the 5′-extremity of the LmSIDER core sequence (LmSIDER2a and LmSIDER2b) were aligned, with the introduction of gaps (-) to maximize the alignments. Identical residues are shaded in grey.

**Figure 5:**
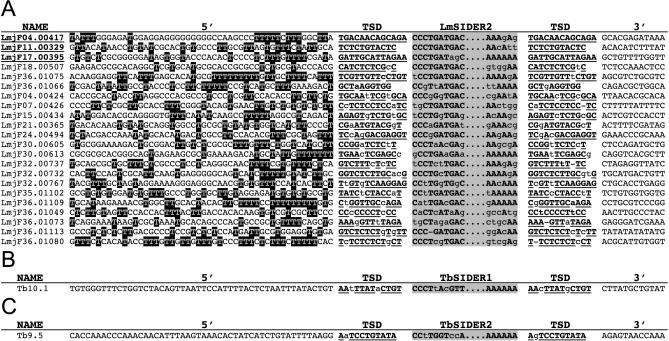
Comparison of the Target Site Duplication (TSD) Flanking LmSIDER2 (A), TbSIDER1 (B), and TbSIDER2 (C) Sequences In the left margin, the name of the element is indicated (the chromosome number is followed by the locus number). In this figure, only the LmSIDER/TbSIDER elements flanked by a TSD presenting two mismatches at the most are shown. The underlined names mean that the corresponding element is flanked by conserved TSD. The alignment of all the selected sequences was based on the retroelement sequences (grey column headed “LmSIDER2/TbSIDER1/TbSIDER2”) from which only the first 10 bp and the last 6 bp are shown (the conserved residues of the retroposon are boldfaced and capital characters). The TSD flanking the retroelements is indicated by boldfaced and underlined capital characters for the conserved residues. Lowercase characters in the TSD column correspond to nonconserved residues. In (A), T residues within the 5′-flanking sequences (called “5′”) that are abundant upstream of the TSD are indicated with white characters on a black background.

Several lines of evidence demonstrate that members of LmSIDER2 are clearly related to retroposons identified in trypanosomes (*ingi*/RIME, L1Tc/NARTc, and DIRE). (i) Two tandemly arranged “79 bp signatures” are found at the 5′-extremity of the LmSIDER2 core (Figures 3 and 4). These are 68% and 62% identical with the first 79 bp residues of the *T. brucei ingi*/RIME. (ii) The 3′-extremity of the LmSIDER2 core sequence is composed of an adenosine-rich stretch, which is a hallmark of retroelements due to the requirement of an RNA intermediate during retrotransposition [39] (Figures 3 and 5). (iii) The LmSIDER2 sequences show a high GC content (65.3%), similar to the one seen in LmDIRE (64.5%), as compared to the rest of the L. major genome (59.7%) (Table 1). The GC content is also higher for the T. brucei RIMEs (53.8%), *ingi*s (52.3%), and TbDIREs (48.7%), as compared to the rest of the T. brucei genome (41%). The relative lower GC content of the TbDIREs compared to the *ingi*/RIME sequences is probably due to the accumulation of point mutations in TbDIREs, as previously observed for extinct retroposons [24]. This interpretation may also explain the relative lower GC content bias observed in the degenerated LmSIDER2 and LmDIRE sequences, compared to the potentially active *ingi* and RIME elements. (iv) As previously observed for the *T. brucei ingi*/RIME and T. cruzi L1Tc/NARTc retroposons, an 18-bp thymidine-rich motif is conserved upstream of LmSIDER2 (Figures 3 and 5). According to the current model of retrotransposition, this sequence motif corresponds probably to the recognition site of the endonuclease encoded by *ingi*/L1Tc-related elements [32,33]. (v) During retrotransposition, the retroposon-encoded endonuclease performs two assymetrical single-strand cleavages, leading to a duplication of the residues between both cleavages. The duplicated motif, flanking the newly inserted retroposons, is called target site duplication (TSD) (Figure 5). One hundred ninety-one LmSIDER2 sequences (18.9% of the aligned LmSIDER2) are flanked by a conserved motif (>75% identity) ranging from 11 bp to 19 bp (69 of them being 13 bp long), which resemble vestiges of TSDs. For three of them, the 11–13-bp TSD is conserved without mismatch (Figure 5A). Interestingly, the size of TSD flanking LmSIDER2 and the *ingi*/RIME/L1Tc/NARTc elements is similar (∼13 bp versus 12 bp) [32,33]. (vi) 90% of the identified LmDIRE sequences (47 out of 52) overlap with a LmSIDER2 sequence at their 5′- and/or 3′-extremities (unpublished data), suggesting that LmDIRE (previously characterized as retroelement vestiges related to *ingi* and L1Tc [38]) and LmSIDER are related. This last observation suggests that LmSIDER was derived from LmDIRE by deletion, as observed for the T. brucei (*ingi*/RIME) and T. cruzi (L1Tc/NARTc) autonomous/non-autonomous pairs of retroposons [30] (Figure 1).

**Table 1:**
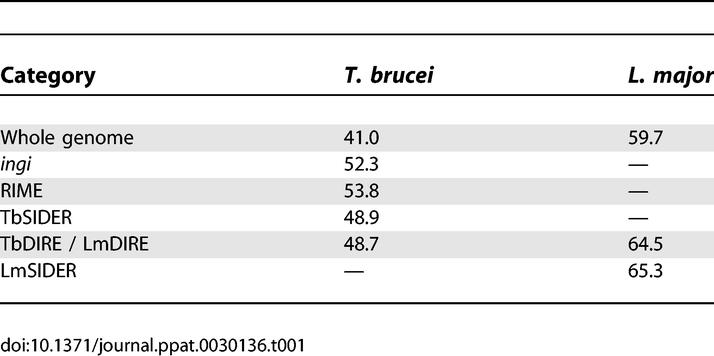
GC Percentage of Trypanosomatid Retroposons

Similarly, both TbSIDER groups show hallmarks of trypanosomatid retroposons, including the presence of the “79 bp signature” (Figure 4) and an adenosine-rich stretch (Figure 5B and 5C) at their 5′- and 3′-extremity, respectively. In addition, one member each of the TbSIDER1 and TbSIDER2 groups is flanked by a degenerated TSD sequence (Figure 5B and 5C).

### SIDERs Are Extinct Retroposons

**Figure 6:**
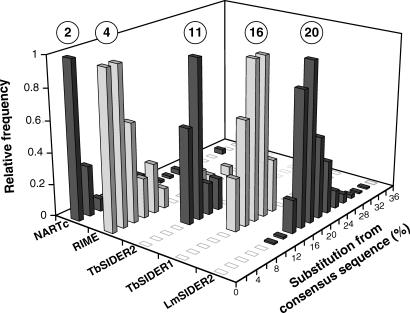
Divergence between Members of LmSIDER2 (1,013 Copies), TbSIDER1 (10 Copies), TbSIDER2 (12 Copies), RIME (70 Copies), and NARTc (115 Copies) Bases covered by the L. major (LmSIDER2), T. cruzi (NARTc), and T. brucei (RIME, TbSIDER1, and TbSIDER2) short retroposons were sorted by their divergence from their consensus sequence. The consensus sequences, determined from the alignment of the core sequence of all the analyzed retroposons, approximate the element's original sequence at the time of insertion. The number of retroposons per fraction of 2% divergence is expressed as a fraction of the highest value, for which an arbitrary value of 1 has been assigned. The percentage of divergence was calculated using the matching region of the consensus sequences. The circled numbers on the top indicate the median value of each graph.

The difficulties encountered in performing the LmSIDER2 alignment reflect the high level of divergence of this TE family. To study the extent of this divergence and gain better insight into the evolutionary dynamics of the LmSIDER2 family, we calculated the percentage of divergence between the consensus LmSIDER2 core sequence deduced from the alignment and each LmSIDER2 core sequence. Since the consensus sequence is assumed to approximate the element's original sequence at the time of insertion, the percentage of substitution from the consensus sequence is correlated to the age of a given element (the age corresponds to the time of retrotransposition). The divergence ranged between 12% and 40%, with median and mean values of 20% and 17%, respectively (Figure 6). The high level of divergence between the consensus and the most conserved LmSIDER2 sequence (12%) implies that LmSIDER became extinct a long time ago.

The same analysis was carried out on the T. brucei RIME/TbSIDER and T. cruzi NARTc elements, which are the only short retroposons characterized so far in the trypanosome genomes [40]. For TbSIDERs, the percentage of divergence from the consensus TbSIDER1 and TbSIDER2 core sequences ranged between 11.6% and 18% and 8% and 13.7%, with median values of 16% and 11%, respectively (Figure 6). This indicates that TbSIDERs are also extinct TEs, as observed for LmSIDERs. In contrast, RIME and NARTc are far more conserved compared to SIDERs (median divergence value of 4% and 2%, respectively) (Figure 6). In addition, 13.8% and 22.5% of the analyzed RIME and NARTc sequences are over 99% identical with the consensus sequence, respectively, indicating recent retrotransposition activities in the trypanosome genomes.

### SIDER Distribution in the L. major and T. brucei Genomes

The T. brucei and L. major genomes are highly syntenic, with approximately 70% of all genes remaining in the same genomic context [40]. This large-scale synteny enables a comparative analysis of TE distribution in these two completed trypanosomatid genomes. The trypanosomatid genomes are characterized by their unique arrangement of DGCs, which are separated by short (0.9–14 kb) divergent or convergent strand-switch regions. For example, the L. major genome (32.6 Mb) has 36 pairs of chromosomes (0.25–2.7 Mb) that are organized into 133 DGCs of tens to hundreds of protein-coding genes (up to 1.26 Mb per DGC) [4]. The T. brucei genome is more compact (26 Mb), with 11 pairs of megachromosomes (1.1–5.5 Mb) containing subtelomeric genes at both extremities, which account for ∼20% of the genome (∼5.2 Mb) [2], while L. major chromosomes do not contain large subtelomeric regions [4].

**Figure 7:**
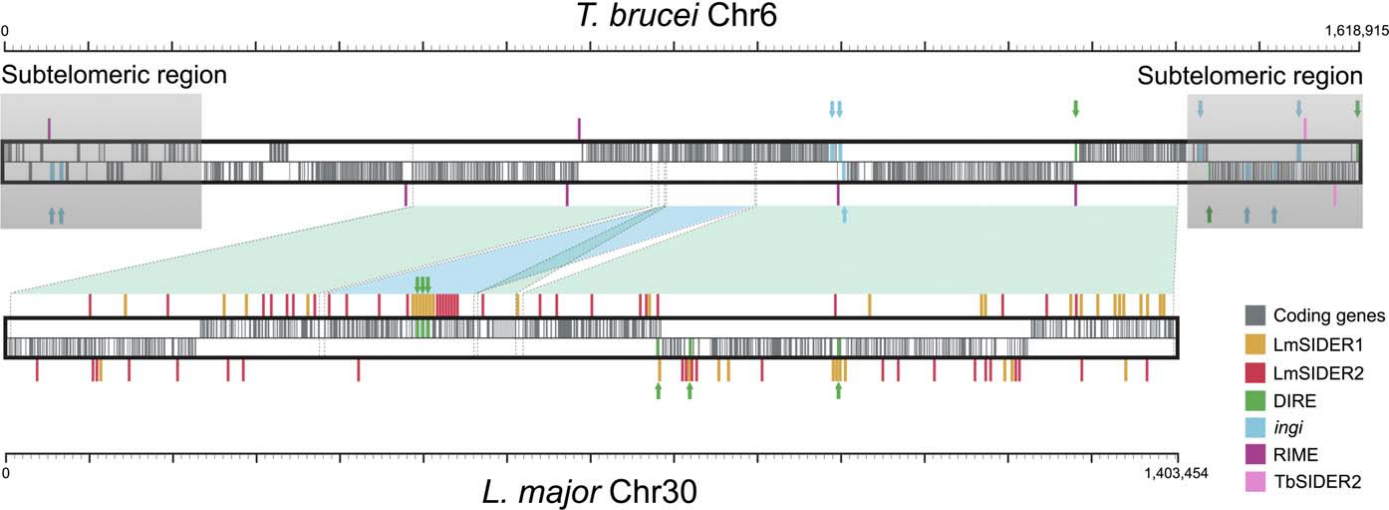
Comparative Analysis of TbChr6 and LmChr30 Syntenic Chromosomes The syntenic regions between the L. major Chromosome 30 (LmChr30) and the T. brucei Chromosome 6 (TbChr6) are represented by blue diamonds. The grey shaded extremities of TbChr6 represent subtelomeric regions primarily composed of pseudogenes. The position of protein-coding genes and retroposons in each chromosome is indicated by vertical bars with the color code displayed on the right margin. Protein-encoding genes and *ingi* and DIRE retroposons are shown on the upper or lower part of the schematic chromosomes, depending on their strand location. Above or below the schematic chromosomes, the other retroposons (LmSIDER1, LmSIDER2, TbSIDER2, and RIME) are indicated, as are the blue and green arrows, which show the position of *ingi* and DIRE retroposons, respectively. The size (bp) of the chromosomes is indicated by the scale bars.

Interestingly, retroposons do not show the same distribution in the L. major and T. brucei genomes (Tables 2 and 3). Indeed, almost all of LmSIDERs and LmDIREs in L. major are located in DGCs (95.4% of the TE), while the *ingi*, RIME, TbDIRE, and TbSIDER retroposons in T. brucei are primarily located in subtelomeric regions (60.1% of the TE). Tables 2 and 3 also show that strand-switch regions display the highest TE richness in both T. brucei and L. major, i.e., over 110 TE per Mb, which corresponds to 23.4% (54 TE) and 4.6% (88 TE) of the retroposons, respectively. The most striking observation is that retroposons are ∼50 times more abundant in L. major DGCs compared to T. brucei DGCs (1,821 versus 38), despite the high level of synteny observed between these regions, which contain an equivalent number of protein-coding genes [40]. This extraordinary difference is the consequence of the unusual distribution and high copy number of LmSIDERs, as exemplified by the comparative analysis of T. brucei Chromosome 6 and L. major Chromosome 30, which are almost completely syntenic (Figure 7) (see Figures S3 and S4 for the other chromosomes).

**Table 2:**
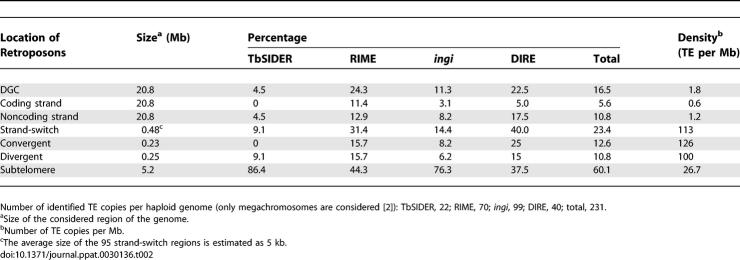
Retroposon Distribution in the T. brucei Genome

**Table 3:**
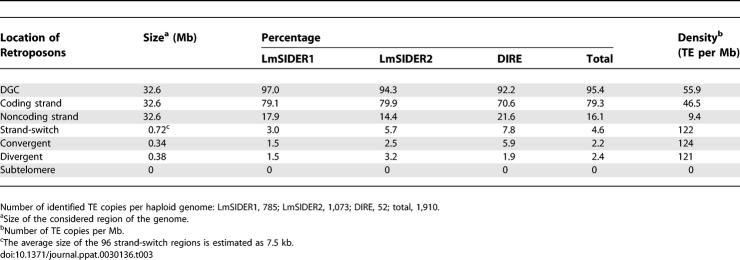
Retroposon Distribution in the L. major Genome

### LmSIDER2 Are Located within the 3′UTR of mRNAs

Since most LmSIDERs are present in the intergenic regions of DGCs, it was important to determine where they are located in regards to the pre-mRNA processing sites. Individual mature mRNAs in trypanosomatids are generated from polycistronic precursors by 5′ *trans*-splicing of a 39-nt capped leader RNA and 3′ polyadenylation [41]. To determine the putative position of polyadenylation sites in L. major, we used the prediction algorithm previously developed for trypanosome mRNA processing sites [9]. There are 8,162 genes annotated in version 4.0 of the L. major genome. The algorithm could predict the vast majority of the 5′UTRs and 3′UTRs of those genes with the exception of 121 5′UTRs (1.5%) and 569 3′UTRs (7%).

**Figure 8:**
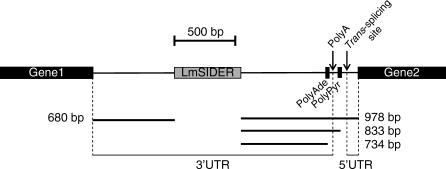
Predominant Localization of LmSIDERs in 3′UTRs Within the intergenic region, *trans*-splicing generally occurs at an AG dinucleotide (*trans*-splicing site) downstream of a long polypyrimidine tract (PolyPyr). Polyadenylation (PolyA) of the upstream cistron takes place possibly as part of a coupled process together with *trans*-splicing. Consequently, the 3′UTR of the *gene1* mRNA (upstream) ends at the putative polyadenylation site (PolyAde), and the 5′UTR of the *gene2* mRNA (downstream) starts at the *trans*-splicing site. This figure shows the average relative position of the polyadenylation sites and the polypyrimidine tracts estimated with a previously developed algorithm [9], as well as the position of the LmSIDERs. The median size between LmSIDERs and the stop codon of the upstream gene (*gene1*), PolyAde, PolyPyr, or the start codon of the downstream gene (*gene2*) are also indicated.

Of the 1,858 LmSIDERs characterized in the L. major genome, 1,356 were found to overlap with a 3′UTR, and 494 have at least one 3′UTR upstream, including 85 LmSIDERs found in strand-switch regions. Conversely, 1,852 have at least one 5′UTR downstream, including 50 LmSIDERs overlapping with the 5′UTR of a gene. Because 73% of the LmSIDERs are found within 3′UTRs, we calculated the median distance of these elements to the upstream stop codon (680 bp) and the downstream ATG (978 bp), as well as the distances from the polypyrimidine tract (833 bp) and putative polyadenylation site (734 bp) (Figure 8). The average location of LmSIDER2s is in the middle of the in silico–predicted 3′UTRs (at almost equal distance from the upstream stop codon and the downstream polyadenylation site), which clearly demonstrates that most LmSIDER2s are located in the 3′UTR of mRNAs.

### LmSIDER2-Containing Transcripts Are on Average Expressed at Lower Levels Relative to Transcripts Lacking LmSIDER2

**Figure 9:**
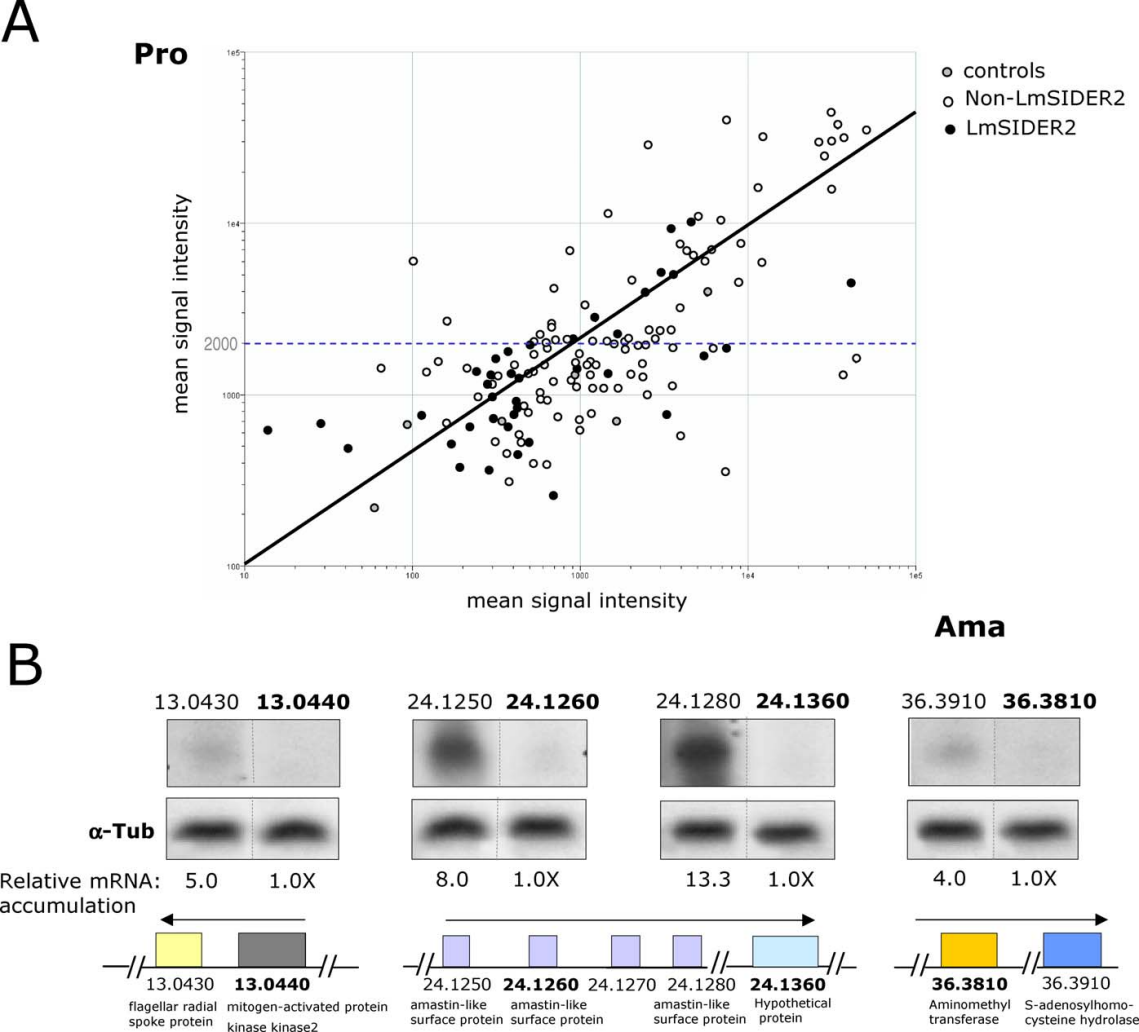
LmSIDER2-Containing mRNAs Are Expressed for the Most Part at Lower Levels Relative to Transcripts Lacking LmSIDER2 (A) Log-scale scatter plot comparing ratios of normalized hybridization intensities between fluorescently labeled L. major promastigotes (Cy3) and amastigotes isolated from mice lesions (Cy5) RNA samples. A custom-designed low density DNA oligonucleotide-based microarray comprising 154 L. major genes, from which only 38 are predicted to harbor LmSIDER2 in their 3′UTR, was used for this study. Total RNA was purified from L. major promastigotes (Pro) grown to mid-log phase and from L. major lesion amastigotes (Ama). Probes were synthesized from total RNA and hybridized to microarrays in quadruplicate. Hybridization experiments were scanned and analyzed using recommended statistic parameters for low spot density arrays in the GeneSpring software. Mean signal intensities of all spots corresponding to one gene were background substracted and normalized with the mean spot intensity of the alien RNA NAC1. Genes were considered as differentially regulated when their expression ratios satisfied a *p*-value below 0.05. The mean signal intensity within the array was calculated to be ∼2,000 (horizontal dotted line). 50% of the genes were identified as significantly differentially expressed. 75% of the LmSIDER2-containing transcripts showed signal intensity lower than the mean intensity of all the spots as compared to 40% for the non-SIDER2 transcripts. (B) The steady-state levels of four pairs of transcripts that are part of the same transcription unit on three different L. major chromosomes were estimated by quantitative northern blotting. LmjF13.0440, LmjF24.1260, LmjF24.1360, and LmjF36.3810 transcripts (in bold) harbor LmSIDER2 in their 3′UTR, whereas LmjF13.0430, LmjF24.1250, LmjF24.1280, and LmjF36.3910 do not. LmjF13.0430 and LmjF13.0440 genes are tandemly linked on Chromosome 13, LmjF24.1250 and LmjF24.1260 are tandemly linked on Chromosome 24, and LmjF24.1280 and LmjF24.1360 genes (chr 24) and LmjF36.3810 and LmjF36.3910 genes (chr 36) are separated by seven and eight genes, respectively. The predicted putative function of the above protein-coding genes is indicated at the lower panel. Equal amounts (∼20 μg) of total RNA were loaded on agarose gel prior to transfer onto a nylon membrane and hybridized with gene-specific probes that were of the same length, GC content, and labeling activity. mRNA levels were quantitated with respect to the amount of total RNA loaded on the gel as verified by hybridization using the alpha-tubulin (α-Tub) gene-specific probe. The normalized numbers are indicated below the blots.

3′UTRs are known to play a key role in regulating gene expression in *Leishmania* [13,15,18,42–46]. The widespread distribution of LmSIDER elements within the *Leishmania* genome and their predominant localization in 3′UTRs, therefore, support the hypothesis that LmSIDER2 may contribute to the regulation of gene expression in this organism. To test this hypothesis, we used custom-designed low density DNA oligonucleotide microarrays to determine expression profiles of LmSIDER2-containing mRNAs in L. major promastigotes and L. major lesion amastigotes isolated from BALB/c mice. Oligonucleotide microarrays were designed to represent 154 L. major genes, from which only 38 bear LmSIDER2 in their 3′UTR. Four independent hybridization experiments were scanned and analyzed using recommended statistic parameters for low spot density arrays in the GeneSpring software. The overall pattern of gene expression for L. major promastigotes and amastigotes is shown in the scatterplot of normalized data in Figure 9A. Approximately 50% of the LmSIDER2-containing transcripts are developmentally regulated in either L. major promastigotes or amastigotes, without any bias towards a particular life stage (24% amastigotes versus 26% promastigotes) and with the majority of genes being constitutively expressed (Figure 9A; Table S2). Interestingly, from these LmSIDER2-containing transcripts, more than 75% have signal intensities that are lower than the mean intensity of all the spots, as compared to 40% for the non-LmSIDER2 transcripts (Figure 9A). The minority of LmSIDER2-containing more abundant transcripts (∼25%) may be explained by a higher degeneracy of LmSIDER2 that results in a nonfunctional element or by the presence of additional elements within the 3′UTR.

To gain independent evidence for the relatively lower expression of LmSIDER2 mRNAs, a randomly selected number of L. major transcripts containing or lacking LmSIDER2 that are most likely clustered within the same transcription unit on three distinct chromosomes were analyzed by quantitative northern blotting. LmjF13.0440, LmjF24.1260, LmjF24.1360, and LmjF36.3810 transcripts harbor LmSIDER2 in their 3′UTR, whereas LmjF13.0430, LmjF24.1250, LmjF24.1280, and LmjF36.3910 do not. LmjF13.0430/LmjF13.0440 and LmjF24.1250/LmjF24.1260 are tandemly linked, whereas LmjF24.1280/LmjF24.1360 and LmjF36.3810/LmjF36.3910 are part of the same transcription unit but are separated by seven to eight genes (Figure 9B). Figure 9B demonstrates that LmSIDER2-containing mRNAs are systematically expressed at much lower levels compared to their co-transcribed genes lacking LmSIDER2. Taken together, these results argue for a more general role of LmSIDER2 in downregulating mRNA expression.

### Mutational Analysis of LmSIDER2 mRNAs Shows That LmSIDER2 Downregulate mRNA Expression Levels

We have recently identified conserved regulatory elements within the 3′UTR of a large set of developmentally regulated transcripts in *Leishmania* and showed that these elements operate principally at the translational level [16,17]. While characterizing the LmSIDER families, we found that these regulatory elements are part of the LmSIDER1 subfamily.

**Figure 10:**
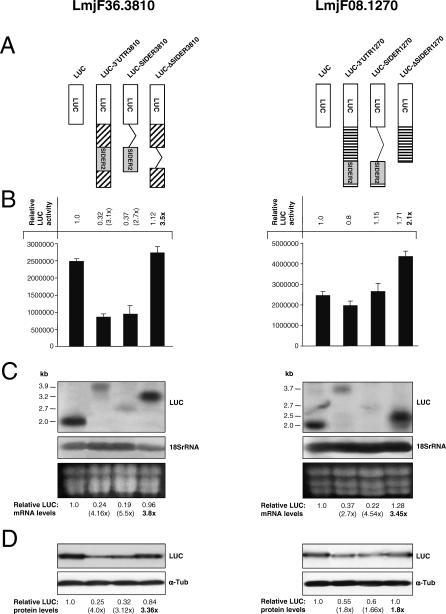
LmSIDER2 Promotes mRNA Downregulation in L. major The potential role of LmSIDER2-containing LmjF36.3810 and LmjF08.1270 3′UTRs in regulating either mRNA or protein levels was evaluated in L. major recombinant parasites grown as promastigotes. (A) Schematic representation of the different LUC chimeric constructs used in this study with the corresponding name indicated on the top. The cross-hatched boxes indicate 3′UTR sequences other than the SIDER sequence. In all the LUC-expressing vectors, the LUC transcript is processed at the 5′-end by sequences from the alpha-tubulin intercistronic region that provides signals for *trans*-splicing (see Materials and Methods). A series of 3′UTR sequences comprising either the full-length 3′UTR (3′UTR-3810, 3′UTR-1270) from the LmjF36.3810 (3,810) and LmjF08.1270 (1,270) transcripts, or the defined LmSIDER2 sequence alone (SIDER-3810, SIDER-1270), or the 3′UTR lacking the LmSIDER2 element (ΔSIDER-3810, ΔSIDER-1270), were cloned downstream of the reporter gene firefly luciferase (LUC). The presence and location of LmSIDER2 within the 3′UTR of *Lmj*F08.1270 and *Lmj*F36.3810 transcripts was verified by 3′UTR mapping. The pSPYNEOαLUC vector (LUC) lacking any regulatory 3′UTR region [16] was used here as a control. The above LUC constructs were introduced by electroporation into the L. major LV39 strain to obtain stable recombinant parasites. The copy number of the different LUC vectors per cell was similar as estimated by Southern blot hybridization (unpublished data). (B) LUC activity was measured as indicated in Materials and Methods. The data were normalized relatively to the control transfectant (LUC). Numbers in parentheses correspond to fold differences in LUC activity with respect to the control LUC strain. The number in bold represents the fold difference in LUC activity compared to the full-length 3′UTR-3810 or 3′UTR-1270. Values are mean + standard error of four independent experiments. (C) Northern blot analysis of total RNA extracted from L. major promastigotes expressing the different LUC-chimeric constructs described in (A). RNA loading on the gel was monitored by hybridization to the 18S rRNA–specific probe. A section of the ethidium-stained gel containing the three ribosomal RNAs is also shown to demonstrate loading. Northern blot hybridization was repeated at least two times and similar results were obtained. (D) Western blot analysis of total protein lysates from L. major–LUC recombinant promastigotes using the anti-LUC antibody. Membranes were stripped and reacted with an anti-alpha-tubulin (α-Tub) antibody to verify protein loading. Western blot analyses were carried out with three different cultures for each transfectant and similar results were obtained. Numbers in parentheses correspond to fold differences relative to the LUC control protein steady-state levels.

We next wanted to obtain direct evidence for the role of LmSIDER2 elements in the regulation of gene expression using luciferase (LUC) as a reporter mRNA. For this, two members of the LmSIDER2 subfamily were selected for further analysis. LmjF08.1270 encodes a hypothetical protein of unknown function [47] and LmjF36.3810 encodes an aminomethyltransferase. Both harbor LmSIDER2 in their 3′UTR. The LmSIDER2 in the LmjF08.1270 transcript (LmSIDER2–1270) is 563 nt long and is located at the end of a 1,531-nt-long 3′UTR (53 nt upstream from the mapped polyadenylation site, unpublished data). In the case of LmjF36.3810, LmSIDER2 (LmSIDER2–3810) is 610 nt long and is located within a 1,831-nt 3′UTR, at 534 nt from the 3′end of the mRNA (see Figure 10A). The sequence identity between the two LmSIDER2 is 60%. The full-length 3′UTR of either LmjF08.1270 or LmjF36.3810 mRNAs was cloned downstream of the LUC reporter gene. LUC reporter constructs with the whole 3′UTR lacking LmSIDER2 or the LmSIDER2 alone were also made (Figure 10A). Each construct was transfected into L. major promastigotes, and stable recombinant parasites were analyzed for LUC activity. Relative LUC activity was calculated by comparing the values obtained with either SIDER2-expressing or SIDER2-lacking recombinant parasites to the LUC control [16]. Figure 9B demonstrates that the LmjF36.3810 3′UTR (LUC-3′UTR3810) results in a 3.1-fold decrease in LUC activity in comparison to the LUC control. A similar decrease (2.7-fold) was obtained with the LmjF36.3810 LmSIDER2 alone (LUC-SIDER3810). Contrasting with this, deletion of SIDER3810 in L. major LUC-ΔSIDER3810 promastigotes caused a 3.5-fold increase in LUC activity with respect to the LUC-3′UTR3810 and LUC-SIDER3810 recombinant parasites. In the case of LUC-3′UTR1270 and LUC-SIDER1270 promastigote cultures, the presence of LmSIDER2 had only a slight effect on LUC activity; however, the deletion of LmSIDER2 in LUC-ΔSIDER1270 resulted in a 2.1-fold increase in LUC activity (Figure 10B), which is consistent with a putative role of LmSIDER2 in regulating LmjF08.1270 gene expression.

To investigate the basis of the differences observed in LUC activity between LmSIDER2-bearing and LmSIDER2-lacking LUC chimeric constructs, we first tested the effect of LmSIDER2 on LUC mRNA abundance by northern blotting. RNA loading on the gel was monitored by hybridization to the 18S rRNA–specific probe. The LmjF36.3810 or LmjF08.1270 LmSIDER2 reduces the levels of LUC chimeric mRNAs by an average of 5-fold with respect to the LUC control mRNA levels (Figure 10C). In contrast to this, deletion of LmSIDER2–3810 or LmSIDER2–1270 retroposons causes a marked increase in LUC mRNA accumulation (3.45- to 3.8-fold). These findings indicate that LmSIDER2 could downregulate mRNA abundance.

To determine the relative contribution of mRNA abundance to the observed LUC activity, we evaluated the level of LUC protein expression derived from the LmSIDER2-containing 3′UTRs by western blotting (Figure 10D). In the case of LUC-3′UTR3810 and LUC-SIDER3810 transfectants, the amount of LUC mRNA dictates the amount of LUC protein. A linear correlation was also observed between LUC-ΔSIDER3810 mRNA accumulation and LUC-ΔSIDER3810 protein levels (Figure 10C and 10D). These findings establish that LmSIDER2–3810 does not alter translational regulation in L. major promastigotes, but rather confers lower mRNA levels. However, although LmSIDER2–1270 clearly contributes to lower steady-state RNA levels, the decrease in mRNA (2.7-fold to 4.54-fold) does not perfectly correlate with LUC protein levels (1.6-fold to 1.8-fold decrease), and LUC activity remained practically unchanged between LUC-3′UTR1270 and LUC-SIDER1270 recombinant parasites in comparison to the LUC control (Figure 10B–10D). These data suggest that in the context of LmjF08.1270, other sequences might compensate for the downregulation effect of LmSIDER2 on mRNA abundance, probably by increasing translation rates.

### LmSIDER2 Are Involved in mRNA Destabilization

**Figure 11:**
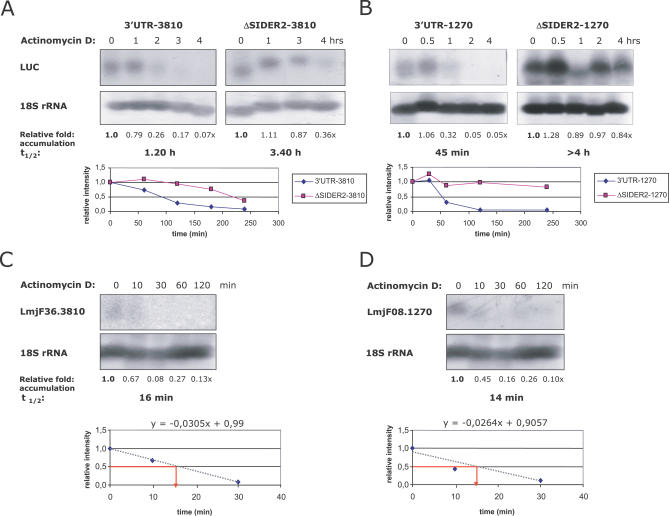
LmSIDER2 Is Involved in mRNA Destabilization (A and B) Comparison of mRNA half-lives (t_1/2_) in L. major promastigotes between LmSIDER2-containing LUC chimeric mRNAs, 3′UTR-3810 (A) and 3′UTR-1270 (B), and LUC mRNAs lacking the LmSIDER2 element, ΔSIDER2–3810 (A) and ΔSIDER2–1270 (B). Approximately 10^7^ L. major recombinant promastigotes/ml were treated with 10 μg/ml of actinomycin D, an inhibitor of de novo transcription, and RNA was isolated at the time points shown and analyzed by northern blotting. Transcript levels were normalized with respect to the amount of the rRNA loaded using the 18S rRNA probe as an internal control. The levels of mRNAs were assessed using phosphorimaging. The values shown below the blots represent the LUC mRNA fold accumulation with respect to its abundance prior to the addition of actinomycin D (0). This is a representative experiment out of two that showed very similar results. (C and D) The decay of the endogenous LmjF36.3810 (C) and LmjF08.1270 (D) transcripts was also assessed in L. major promastigotes using actinomycin D (using northern blotting). The half-lives of LmSIDER2-containing LUC chimeric mRNAs and LmjF36.3810 and LmjF08.1270 endogenous transcripts were estimated based on hybridization intensities with gene-specific probes normalized with the 18S rRNA probe with respect to different time points of actinomycin D treatment. Experiments shown here are representative of two that showed very similar results.

As regulation of gene expression in *Leishmania* is known not to occur at the transcriptional level, and as there is virtually no evidence for differential splicing [10], the most likely mechanism for lower abundance of LmSIDER2 mRNAs is through altered mRNA stability. To examine whether lower accumulation of LmSIDER2–3810- and LmSIDER2–1270-containing LUC chimeric transcripts in L. major promastigotes could be due to mRNA destabilization, we measured half-lives of the LUC transcripts that bear or lack LmSIDER2 using actinomycin D treatment to block de novo transcription and northern blot hybridization to visualize mRNAs. Analysis of the data revealed that LUC-3′UTR1270 and LUC-3′UTR3810 transcripts have half-lives of 45 min and 80 min, respectively (Figure 11A and 11B). LmSIDER2 deletion resulted in a marked increase of the half-life of the LUC transcript by 3.0- to 5.5-fold, respectively (Figure 11A and 11B). We also evaluated the half-lives of the single copy endogenous LmjF36.3810 and LmjF08.1270 mRNAs, which are very short (∼16 and 14 min, respectively) (Figure 11C and 11D). The differences in the half-lives observed between the endogenous and the episomal LmSIDER2-containing transcripts can be explained by the higher copy number (∼35) of the latter compared to that of the former.

## Discussion

The discovery of transposable elements in trypanosomatid genomes was recently advanced by the completion of the T. brucei, T. cruzi, and L. major genomic sequence [2–4]. Here, we describe a newly discovered family of extinct retroposons in *Leishmania*, named LmSIDER2 (1,073 copies), that are predominantly located in the 3′UTR of mRNAs, and show that members within this family play a role in the regulation of gene expression.

### Evolution of Autonomous/Non-Autonomous Retroposon Pairs in Trypanosomatids

The genomes of higher eukaryotes contain pairs of autonomous/non-autonomous retroposons composed of small noncoding elements, which use for their own mobility the retrotransposition machinery encoded by autonomous elements (for review see [48]). This is exemplified by the retroposon pairs described in human (LINE1/Alu, LINE2/MIR, and LINE2/Ther-1) [24,34,37], fish (UnaL2/UnaSINE1) [36], reptiles (CR1-like LINE/SINE) [49], and plants (Bali1/S1) [35]. In these examples, the small noncoding elements, called small interspersed elements (SINEs), are tRNA-, 5S RNA–, or 7SL RNA–related sequences [50–52]. In contrast, the small noncoding partners (RIME and NARTc) of the trypanosome *ingi*/RIME (T. brucei) and L1Tc/NARTc (T. cruzi) pairs are derived from the autonomous retroposons (*ingi* and L1Tc) by deletion of the coding sequence [27–31]. The truncated RIME and NARTc elements became fixed in the trypanosome genome with copy numbers equivalent to that of the autonomous *ingi* and L1Tc retroposons (see Table 1) [32,33]. In addition, all trypanosomatid genomes analyzed so far contain degenerated retroposons related to *ingi* and L1Tc (DIRE) [38]. The majority of LmDIRE sequences identified in the L. major genome (90%) overlaps with a subset of LmSIDER, suggesting that the latter are derived from the former by deletion. This indicates the existence of an LmDIRE/LmSIDER pair comparable to the trypanosome *ingi*/RIME and L1Tc/NARTc pairs.

The trypanosome *ingi*/RIME and L1Tc/NARTc pairs are considered active, since the very low level of sequence divergence observed is consistent with recent retrotransposition activities. The T. brucei and T. cruzi genomes contain several potentially active *ingi*/L1Tc, which encode a single long and conserved protein [3,32,33]. This contrasts with the L. major LmDIRE/LmSIDER pair, which has lost its retrotransposition activity. Indeed, the only reverse transcriptase domains identified in the completed L. major genome belong to LmDIREs, which have accumulated numerous point mutations after their extinction [38]. In the absence of functional retroposons, the noncoding LmSIDER families can be considered extinct as well, since their members need enzymes produced in *trans* by autonomous retroposons for their mobilization. Consequently, the LmSIDER and LmDIRE families probably became extinct simultaneously, when the last active LmDIRE disappeared from the L. major genome, as proposed for the extinct human LINE2/MIR and rodent LINE1/B1 pairs [24,53]. The simultaneous extinction of the human autonomous LINE2 and non-autonomous MIR retroposons is illustrated by their similar nucleotide substitution level [24]. However, this comparative analysis cannot be done for the LmDIRE/LmSIDER pair because of the inappropriately low number of LmDIRE sequences available for such a statistical analysis [38]. The high level of divergence (12%) between the consensus and the most conserved LmSIDER2 sequence suggests that LmSIDER became extinct a long time ago. The rise and fall of TE families has been well documented in several genomes [19,24]. For example, it was estimated that the human LINE2 retroposons, which show at least 18% divergence with the consensus LINE2 sequence, became extinct 50–100 million years ago [19]. In the absence of trypanosomatid fossil records and thus of a molecular clock, the date of LmSIDER extinction cannot be estimated with accuracy. It probably occurred after the speciation of the *Trypanosoma* and *Leishmania* genus 200–500 million years ago [54], since trypanosomes still contain putative active elements [38].

### Exaptation of LmSIDERs by L. major and Their Role in Modulating Gene Expression

The recent completion and comparative analysis of eukaryotic genomes provides evidence that several superfamilies of short non-autonomous retroposons (e.g., SINE) have been conserved and distributed among a wide range of species [55–57]. These conservations suggest that numerous extinct retroposons were domesticated hundreds of million years ago and are still functional in several species. While superfamilies of retroposons are conserved and shown to be functional, exaptation of a TE family to the extent described here has not been reported so far. Here, we provide evidence that Leishmania spp. have recycled a whole family of short retroposons (LmSIDER2), which have evolved to fulfill important biological pathways such as the regulation of gene expression, whereas its close relative T. brucei developed other approaches to maintain similar cellular functions. Retroposon-mediated regulation at transcriptional or post-transcriptional levels [23,48,58–61] remains a relatively rare event in other eukaryotes and is not thought to be an intrinsic function of retroposons. Most LmSIDERs (95.4%) are located within intergenic regions of DGCs, mainly in 3′UTRs, while 95.5% of the TbSIDERs are located outside DGCs; the retroposon density in DGCs being ∼50 times higher in L. major than T. brucei. This contrasting SIDER distribution can also be correlated with the difference in the average size of intergenic regions between L. major and T. brucei (1,432 bp versus 721 bp) [40], in part due to the presence of LmSIDERs in the 3′UTRs.

We have previously identified a conserved 450–550-bp element located in the 3′UTR of several *Leishmania* amastigote–specific transcripts that is implicated in stage-specific translational control [16,17]. Interestingly, this element belongs to the LmSIDER1 subfamily of retroposons, which comprises at least 785 sequences across the *Leishmania* genome (A. Rochette, M. Smith, P. Padmanbhan, B. Papadopoulou, unpublished data). In this study, we presented several lines of evidence showing that LmSIDER2 promotes mRNA destabilization. This conclusion stems from a comprehensive microarray analysis, from northern blotting data, and from a more direct reporter gene analysis of selected mRNAs. The functional distinction between LmSIDER1 and LmSIDER2 is consistent with the way they clustered in a phylogenetic tree.

The ability of LmSIDER2 to destabilize mRNA seems to be intrinsic and context independent, since it can be functional at different distances from the poly(A) tail and even outside the context of the endogenous 3′UTRs (see Figure 10). LmSIDER2-containing mRNAs are generally expressed at lower levels compared to non-SIDER2-bearing transcripts and are short-lived (half-lives of ∼15 min). Taken together, these observations suggest that LmSIDER2 are *cis*-acting components of a regulatory pathway that generally downregulates gene expression to ensure rapid turnover of a specific subset of *Leishmania* mRNAs. Throughout its complex life cycle, *Leishmania* is subjected to a variety of rapidly changing environmental conditions, and rapid mRNA turnover can permit the parasite to adapt its pattern of protein synthesis to continuously changing physiological needs. We hypothesize that the mRNA-destabilizing function of LmSIDER2 can be enhanced or blocked as needed due to their particular sequence or structure (LmSIDER2 elements are highly heterogeneous), and/or the presence of other elements in the 3′UTR of *Leishmania* transcripts. This is in agreement with our preliminary results in L. infantum amastigotes, where the 36.3810 SIDER2 becomes inactive due to the presence of a downstream element (M. Müller, B. Papadopoulou, unpublished data), and with the observation that none of the highly expressed housekeeping genes harbor LmSIDER2 (unpublished data).

In the case of LmjF36.3810 and LmjF08.1270 transcripts, which are both constitutively expressed in L. major, the LmSIDER2 destabilizing element works as efficiently in amastigotes as it does in promastigotes (unpublished data). Our microarray data on 38 LmSIDER2-containing transcripts are also consistent with these observations. However, other stages of the parasite, irrespective of whether they are morphologically distinct (e.g., metacyclics) or not, exist where the function of these elements might be more crucial. Indeed, we also found that several transcripts reported to be upregulated in the metacyclic stage of L. major [62] contain LmSIDER2 (unpublished data). Likewise, the role of these elements might be more evident as the parasite experiences a specific environmental challenge, particularly in the rather dynamic ecological niche inside its insect host. Indeed, a number of short-lived mRNAs are known to be responsive to specific extracellular environmental stimuli in other systems where expression is regulated by sequences in 3′UTRs (e.g., the AU-rich elements of inflammatory cytokines and growth factors) [63,64]. Alternatively, the role of LmSIDER2 might be to negatively modulate gene expression and thereby check that mRNAs, stage-specific or constitutively expressed, are maintained at nontoxic levels (for instance, mRNAs encoding structural proteins are generally expected to be more abundant than those encoding regulatory proteins).

Comparison of the L. major and T. brucei genomes showed that SIDERs are ∼70 times more abundant in L. major compared to T. brucei [38]. Considering that the majority of LmSIDERs is co-transcribed with coding genes and that members of the LmSIDER families are shown to play a role in the regulation of gene expression, whereas most of the very few TbSIDERs are distributed in the relatively silent subtelomeric regions, it is tempting to propose that *Leishmania*, but not trypanosomes, have exapted and expanded the SIDER retroposons. The reasons behind this extraordinary LmSIDER expansion are currently unknown. The widespread genomic distribution of LmSIDER2 and our functional data on both LmSIDER1 and LmSIDER2 members raises the interesting possibility that numerous *Leishmania* transcripts encoding a wide repertoire of functionally diverse proteins may be regulated by a similar mechanism in response to specific environmental stimuli and/or growth conditions. The involvement of TE in coordinated expression of genes was already proposed in the seventies [65].

We propose that *Leishmania*, an organism with no known control at the level of transcription initiation, has acquired the ability to post-transcriptionally coordinate gene regulation via short retroposons (LmSIDERs) in the 3′UTR. This is consistent with the prevailing notion that retroelements likely emerged as genomic parasites and gradually invaded the genomes of most eukaryotic cells, but later became an integral part of their genome and were used for the benefit of these organisms.

## Materials and Methods

### Identification of LmSIDERs.

A BLASTN search of the L. major genome with the first 79 residues of the *T. brucei ingi*/RIME (“79 bp signatures”) revealed 108 homologous sequences, corresponding to the 5′-extremity of degenerated retroposons, subsequently called LmSIDERs. A multiple alignment (ClustalW [66]) of the sequences located downstream from these 108 “79 bp signatures” (1 kb) was then done to define six groups of related but very heterogeneous sequences, ranging from 450 to 790 bp in length. The 3′-extremity of most of these relatively conserved sequences was composed of an adenosine-rich stretch, as generally observed for retroposons. In order to identify other LmSIDER in the L. major genome, a second BLASTN search was performed with one representative from each group of sequences. About 1,500 matches were retained. A third BLASTN search conducted with a subset of very divergent LmSIDER identified new sequences. Some of these newly identified LmSIDER were used for a fourth BLASTN search. We stopped this reiterative BLASTN search approach after two additional runs, since no more sequences were detected, with a total of 1,858 identified LmSIDER elements. The BLASTN analysis also revealed that LmSIDER could be separated into two groups composed of 785 (LmSIDER1) and 1,073 (LmSIDER2) sequences (see Figure 2).

### Identification of TbSIDERs.

The first 79 residues of the *T. brucei ingi*/RIME (“79 bp signatures”) were used to perform a BLASTN search of the T. brucei genome database (version 3.0 of The Institute for Genomic Research's [TIGR] T. brucei assembly). For this BLAST analysis, the annotated RIME, *ingi*, and DIRE sequences were masked using Repeat Masker (http://www.repeatmasker.org/). A multiple sequence alignment (ClustalW [66]) of the regions located downstream of 51 identified “79 bp signatures” (1 kb) and defined two groups of related sequences, named TbSIDER1 (ten sequences) and TbSIDER2 (12 sequences), while the other 29 sequences were unique and appeared not to be related to retroposons.

### Multiple alignments and phylogenetic analysis of LmSIDER sequences.

We used ClustalW (http://www.ebi.ac.uk/tools/clustalw/), MUSCLE (http://www.drive5.com/muscle/), and 3DCoffee (http://igs-server.cnrs-mrs.fr/Tcoffee/tcoffee_cgi/index.cgi) programs to perform a multiple sequence alignment of all (1,073 sequences) or different subsets of (from 50 sequences) LmSIDER2. None of these attempts produced in and of themselves a satisfactory alignment, probably because of the high degree of divergence and size polymorphism. The MUSCLE program produced a workable alignment from a selection of 50 full-length and relatively closely related LmSIDER2 sequences. This multiple alignment was manually refined to generate a framework used to manually align, one by one, the LmSIDER2 sequences. The final alignment contained 1,013 LmSIDER2 sequences (Figure S1) The LmSIDER2 core sequence was generated by deleting all positions showing a gap for at least 50% of the aligned sequences, which represents 66.6% of the positions (1,074 positions out of 1,612 in the original alignment) (Figure S2). The statistical and comparative analyses were performed using this LmSIDER2 core sequence.

LmSIDERs were extracted from the most recent L. major genome annotation (http://www.genedb.org/) for phylogenetic analysis. We extracted SIDER (formerly named LmRIME) sequence regions between 400 and 700 nucleotides long using Artemis [67]. An automated multiple sequence alignment was generated by comparing individual sequences to a Hidden Markov Model (HMM) using HMMER 1.8.5 (http://hmmer.janelia.org/). The HMM profile used to align the LmSIDERs was generated using 15 representative sequences selected from the manual alignment shown in Figure S1 (24.0477, 36.1076, 29.0524, 31.0641, 33.0760, 36.1128, 36.1087, 35.1074, 34.0878, 31.0653, 25.0573, 38.0225, 34.0863, 14.0386, 28.0581). Limiting the amount of sequences in the profile minimizes position-specific base composition bias. To facilitate visualization of the subsequent tree, we removed additional LmSIDERs displaying >95% identity to at least one other aligned sequence using an ad-hoc JAVA script (http://java.sun.com/). The final alignment contains 785 LmSIDER sequences (140 LmSIDER1 and 645 LmSIDER2). The 785 resulting LmSIDERs were submitted to a Minimum Evolution phylogenetic analysis based upon the number of differences using the MEGA3 program [68]. Furthermore, only parsimonious informative sites were considered. The phylogenetic tree was displayed using HyperTree JAVA program [69].

### Divergence between members of retroposon families.

TbSIDER1 (ten), TbSIDER2 (12), RIME (70), and NARTc (115) sequences were separately aligned using ClustalW (http://www.ebi.ac.uk/tools/clustalw/), whereas LmSIDER2 sequences (1,013) were manually aligned as described above. The core sequences, deduced from these alignments, were defined as described above for the LmSIDER2 core sequence. 21, 2, 44, and 19 positions were removed from the original TbSIDER2, TbSIDER1, RIME, and NARTc alignments, which corresponds to 4%, 0.4%, 8.1%, and 6.7% of the positions, respectively. The core consensus sequences were reconstituted by considering the most conserved residue at each position of the alignment. Then, the percentage of substitution from the consensus was determined for each sequence aligned by calculating the sequence identity of each sequence with the consensus. The consensus sequence was created with BioEdit (http://www.mbio.ncsu.edu/BioEdit/bioedit.html) using a threshold frequency for inclusion of 26%. Gaps were treated like residues.

### Statistical analysis.

To quantify the degree of conservation at each column in the core sequence multialignments, a chi-square (*χ* ^2^)score was computed comparing the observed distribution of ACGTs in the column to the distribution in the entire genome. The background ACGT distribution for the genome was obtained by counting the occurrences of each base in the set of all assembled chromosomes. Then, in each of the four multiple-alignments at each column, the chi-square score was computed as

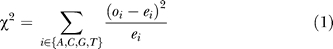
where *o_i_* is the observed number of occurrences of character *i* in the given column, and *e_i_* is the expected number of occurrences of character *i* computed as the proportion of character *i* in all assemblies multiplied by the number of sequences in that column of the multialignment. Using three degrees of freedom, a *χ* ^2^ value of 16.3 corresponds to a significance level of *p* < 0.001.

### Determination of mRNA processing sites.

The chromosomes and genomic coordinates of all L. major coding sequences were retrieved from version 4.0 of the assembly and annotation database hosted at TIGR. Using the predictive algorithm developed by Benz et al. [9], we scanned all L. major chromosomes to locate the putative polypyrimidine tract and splice acceptor and polydenylation sites for each gene, thus delimiting the coordinates of the putative 5′UTR and 3′UTR. We selected the splice acceptor signal nearest to the start codon. This choice was based on what was observed in T. brucei, where EST mapping validated that 66% of the genes primarily used the closest site [9]. The distance between each LmSIDER element and its closest downstream and upstream gene on each chromosome strand was computed, disregarding the strand on which the element was located. The distance between each LmSIDER and the first methionine codon of the nearest downstream gene was calculated to determine a list of LmSIDERs that overlapped with the in silico*–*predicted 3′UTRs or 5′UTRs. Then, the distance between 3′UTR overlapping LmSIDERs and polypyrimidine and polyadenylation sites of the overlapping gene was calculated.

### *Leishmania* culture.

The L. major LV39 strain used in this study was described previously [70]. Promastigotes were cultured at pH 7.0 and 25 °C in SDM-79 medium supplemented with 10% heat-inactivated FCS (Wisent, http://www.wisent.ca/) and 5 μg/ml hemin. Intracellular L. major amastigotes were isolated from footpad lesions of infected BALB/c mice as previously described [71].

### Plasmid construction and transfections.

The expression vector pSPYNEOαLUC was described previously [16] and is referred to as *LUC*-control in the present study. The *LUC*-chimeric mRNAs transcribed from this vector are processed in *Leishmania* using sequences within the alpha-tubulin intergenic region cloned at the 5′-end. The different *LUC*-chimeric constructs listed in Figure 10 were made as follows. The full-length 3′UTR of LmjF36.3810 and LmjF08.1270 transcripts from the termination codon to 434 bp beyond the poly(A) site in the case of LmjF36.3810, and to 84 bp beyond the poly(A) site in the case of LmjF08.1270, or the LmSIDER2 element or the 3′UTR lacking LmSIDER2, were amplified by PCR using Taq DNA polymerase (Qiagen, http://www.qiagen.com/) and primers with inserted BamHI or PstI restriction sites (see Table S3). PCR products were cloned into vector pCR2.1 (Invitrogen, http://www.invitrogen.com/), digested with BamHI or PstI (New England Biolabs, http://www.neb.com/) and subcloned into the BamHI site downstream of the *LUC* gene in vector pSPYNEOαLUC [16]. All constructs have been verified by sequencing. Purified plasmid vector DNA (10–20 μg, Qiagen) were transfected into *Leishmania* by electroporation as described previously [72]. Stable transfectants were selected with 0.04 mg/ml G-418 (Sigma, http://www.sigmaaldrich.com/).

### LUC assay.

The LUC activity of the recombinant parasites was determined as described previously [17]. Briefly, mid-log-phase promastigotes were diluted 1:100 in SDM-79 supplemented with 10% glycerol and counted in a Neubauer counting chamber. Equivalents of 4 × 10^7^ and 2 × 10^7^ parasites were spun, the pellet resuspended in 5× luciferase lysis (Promega, http://www.promega.com/) buffer and frozen at −80°C. Twenty μl of each lysate was then mixed with an assay buffer (Promega) containing D-luciferin potassium salt, and LUC activity was measured in a luminometer (Dynex MLX, http://www.dynextechnologies.com/).

### RNA and protein manipulations.

Total RNA of L. major promastigotes was isolated using the TRIzol reagent (Gibco BRL, http://www.invitrogen.com/) following manufacturer instructions. Northern blot hybridizations were performed following standard procedures [73]. To prepare soluble protein lysates, *Leishmania* cells were harvested by centrifugation, washed with ice-cold phosphate-buffered saline (PBS), resuspended in Laemmli buffer, and syringed with a microsyringe (ten times). Proteins were quantified using Amido Black 10B (Bio-Rad, http://www.bio-rad.com/), and 50 μg of total protein extracts were loaded onto 10% SDS-PAGE gels. The gels were transferred on a polyvinylidene difluoride membrane (Immobilon-P; Millipore, http://www.millipore.com/) and the membranes were incubated for 90 min in blocking buffer (PBS with 0.1% Tween 20 and 5% nonfat dry milk). The first antibody, a goat anti-luciferase pAB (Promega) diluted 1:10,000 in blocking buffer, was incubated with the membrane for 90 min with agitation. Following three washes with PBST (PBS supplemented with 0.1% Tween 20), a second antibody, a donkey anti-goat (Santa Cruz Biotechnology, http://www.scbt.com/) diluted 1:10,000 in blocking buffer, was incubated for 45 min with the membrane. After additional washes, the blot was visualized by chemiluminescence with a Renaissance kit (New Life Science Products, http://las.perkinelmer.com/). RNA and protein levels were estimated by densitometric analyses using a PhosphorImager with ImageQuant 5.2 software.

### RNA stability assays*.*

To determine the half-life of LmSIDER2-containing transcripts, mid-log phase L. major promastigote cultures were incubated with 10 μg/mL of actinomycin D (Sigma), an inhibitor of de novo transcription. At specific times post-addition of the drug, 10-ml culture aliquots were pelleted by centrifugation, washed once with Hepes-NaCl buffer, and lysed in 1 ml TRIzol reagent (Gibco BRL). Total RNA was extracted from these samples and subjected to northern blot hybridization. Quantitation of the different transcripts was done by densitometric analysis using a PhosphorImager with the ImageQuant 5.2 software.

### DNA microarray analysis and quantitative real-time RT-PCR.

Thirty-eight L. major genes predicted to harbor LmSIDER2 in their 3′UTR were chosen for DNA microarray analysis, as part of a previously described 70-mer oligonucleotide array comprising a total of 154 selected genes [47]. Total RNA from L. major promastigotes and lesion amastigotes isolated from infected BALB/c mice was prepared using the TRIzol reagent (Gibco BRL) and purified using the RNAeasy kit (Qiagen). Quality and quantity of the RNA was assessed by RNA 6000 Nano Assay Chips (Agilent Technologies, http://www.home.agilent.com/) and a Bioanalyzer (Agilent Technologies). Probes for microarray hybridization were prepared using the indirect Micromax TSA labeling and detection kit (Perkin Elmer, http://las.perkinelmer.com/). For each labeling reaction, 2 μg of purified RNA was spiked with two exogenous mRNAs (*NAC1* and *CAB1* from Arabidopsis thaliana at 2.5 pg/μl; Stratagene, http://www.stratagene.com/) to adjust for variations in the incorporation efficiency of the modified nucleotides and differences in first-strand cDNA synthesis reactions. Hybridization, washes, and detection of fluorescence were done as described previously [47]. Four independent microarray experiments including dye swapping were scanned, and signal intensities for each spot were exported into GeneSpring software (Agilent) for further analysis. Local background was subtracted from each spot on the array, and intensity-dependent normalization was carried out within arrays. Cy5/Cy3 ratio for each spot was normalized with Cy5/Cy3 ratio for the *A. thaliana NAC1* spike. Genes were only considered as statistically different in their expression if they satisfied a *p*-value cutoff of 0.05. Expression ratios of three LmSIDER2-containing genes (LmjF31.1890, LmjF33.2550, LmjF08.1270) and one non-LmSIDER2 gene (LmjF16.1430) were confirmed by quantitative real-time RT-PCR as described previously [47]. These ratios were normalized using the *GAPDH* ratio to give a fold difference of expression. To exclude eventual amplification of mouse transcripts, cDNA from mouse macrophages served as negative control in each experiment.

## Supporting Information

Figure S1 Alignment of 1,013 LmSIDER2 The alignment, saved under the Philip format, was performed as described in Materials and Methods with the introduction of gaps (-) to maximize the alignments. The LmSIDER names indicate the chromosomal localization followed by the model number.
(1.6 MB DOC)

Figure S2 Alignment of the Core Sequence of 1,013 LmSIDER2 This alignment was generated from the one presented in Figure S1 by deleting all positions showing a gap for at least 50% of the aligned sequences.
(553 KB DOC)

Figure S3 Distribution of Genes and Retroposons on the 36 L. major Chromosomes The central scale bars showing the size of the chromosomes (kb) separate features located on different strands. The position of protein-encoding genes and retroposons is indicated by vertical bars with the color code shown on the right margin. Protein-encoding genes and DIREs are shown on both central panels, while the upper or lower part of the schematic chromosomes display the position of LmSIDER1 and LmSIDER2.
(888 KB DOC)

Figure S4 Distribution of Genes and Retroposons on the 11 T. brucei Megachromosomes The central scale bars showing the size of the chromosomes (kb) separate features located on different strands. The position of protein-encoding genes and retroposons is indicated by vertical bars with the color code shown in the right margin. Protein-encoding genes and *ingi* and DIRE retroposons are shown in both central panels, while the upper or lower part of the schematic chromosomes indicate the position of RIME and TbSIDER retroposons.
(856 KB DOC)

Table S1 LmSIDER Sequences Annotated in the L. major Genome The chromosome localization (“chr”), genomic coordinates (“start” and “end”), strand localization (“str”), family (“fam”), and name (“name”) of the annotated LmSIDERs are indicated. The first column (“ID”) shows the name of each LmSIDER annotated in the database (version 4.0 of the assembly) hosted at The Institute for Genomic Research. The last column (“chr_size”) indicates the size of the chromosomes.
(194 KB PDF)

Table S2 Differential Gene Expression of L. major SIDER2-Containing Transcripts Analyzed by DNA Microarrays (77 KB PDF)

Table S3 Primers Used for the Generation of the LUC-Expressing Vectors (59 KB PDF)

## Abbreviations

DGC: directional gene cluster
DIRE: degenerated *ingi*-related element
LINE: long interespersed element
LTR: long-terminal repeat
LUC: luciferase
RIME: ribosomal mobile element
SIDER: short interspersed degenerated retroposon
SINE: short interspersed element
TE: transposable element
TSD: target site duplication
UTR: untranslated region
