# Supplementary material for: Members of a Large Retroposon Family Are Determinants of Post-Transcriptional Gene Expression in Leishmania
Source: PLoS Pathog. 2007 Sep 28;3(9):e136. doi: 10.1371/journal.ppat.0030136 (PMC2323293; doi:10.1371/journal.ppat.0030136)
Supplement: Figure S3 — The central scale bars showing the size of the chromosomes (kb) separate features located on different strands. The position of protein-encoding genes and retroposons is indicated by vertical bars with the color code shown on the right margin. Protein-encoding genes and DIREs are shown on both central panels, while the upper or lower part of the schematic chromosomes display the position of LmSIDER1 and LmSIDER2. (888 KB DOC) [file ppat.0030136.sg003.pdf]

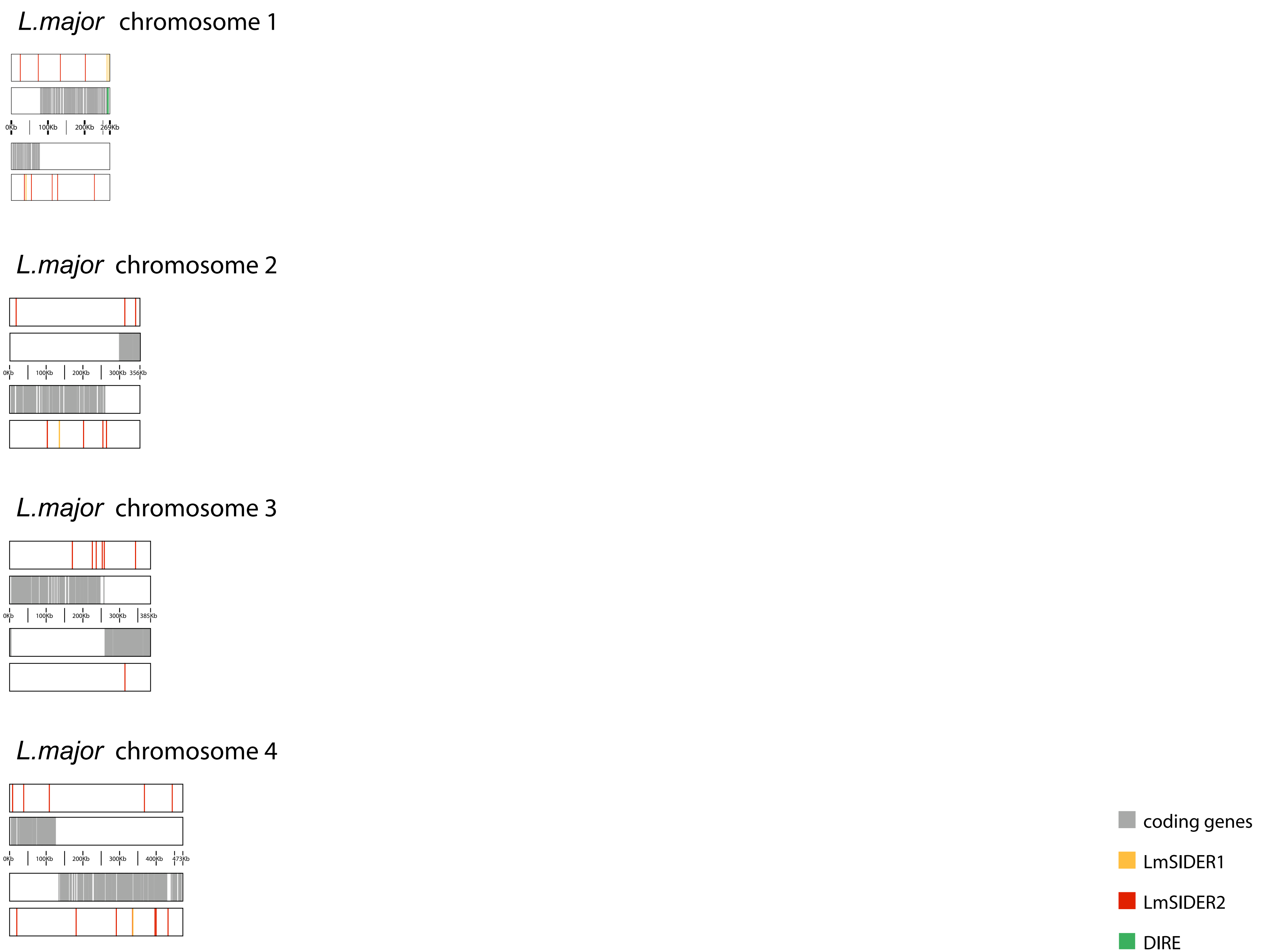
L.major chromosome 1
0Kb 100Kb 200Kb 269Kb
L.major chromosome 2
0Kb 100Kb 200Kb 300Kb 356Kb
L.major chromosome 3
0Kb 100Kb 200Kb 300Kb 385Kb
L.major chromosome 4
0Kb 100Kb 200Kb 300Kb 400Kb 473Kb
coding genes
LmSIDER1
LmSIDER2
DIRE

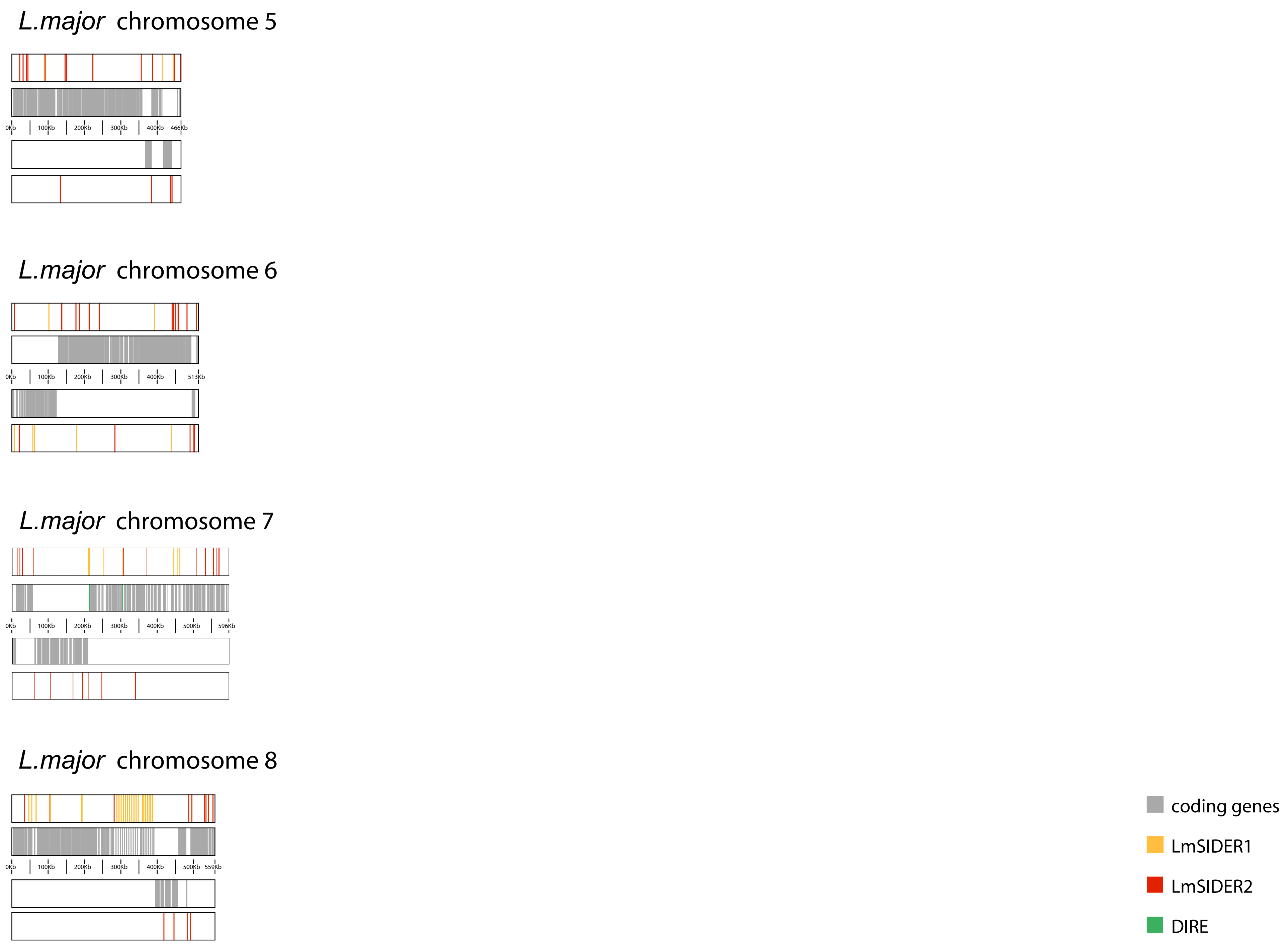

L.major chromosome 5
0Kb
100Kb
200Kb
300Kb
400Kb
466Kb
L.major chromosome 6
0Kb
100Kb
200Kb
300Kb
400Kb
513Kb
L.major chromosome 7
0Kb
100Kb
200Kb
300Kb
400Kb
500Kb
596Kb
L.major chromosome 8
0Kb
100Kb
200Kb
300Kb
400Kb
500Kb
559Kb
coding genes
LmSIDER1
LmSIDER2
DIRE

L.major chromosome 9

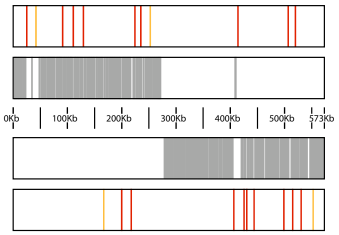

L.major chromosome 10

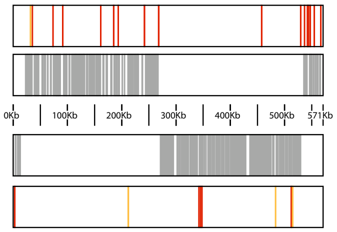

L.major chromosome 11

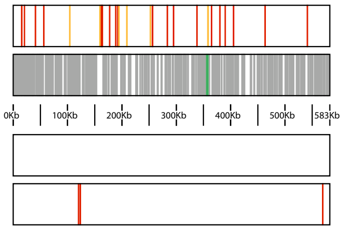

L.major chromosome 12

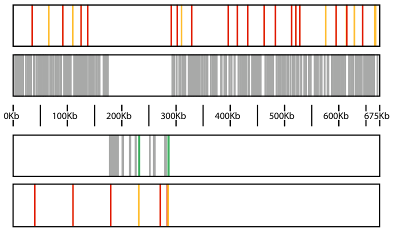

coding genes
LmSIDER1
LmSIDER2
DIRE

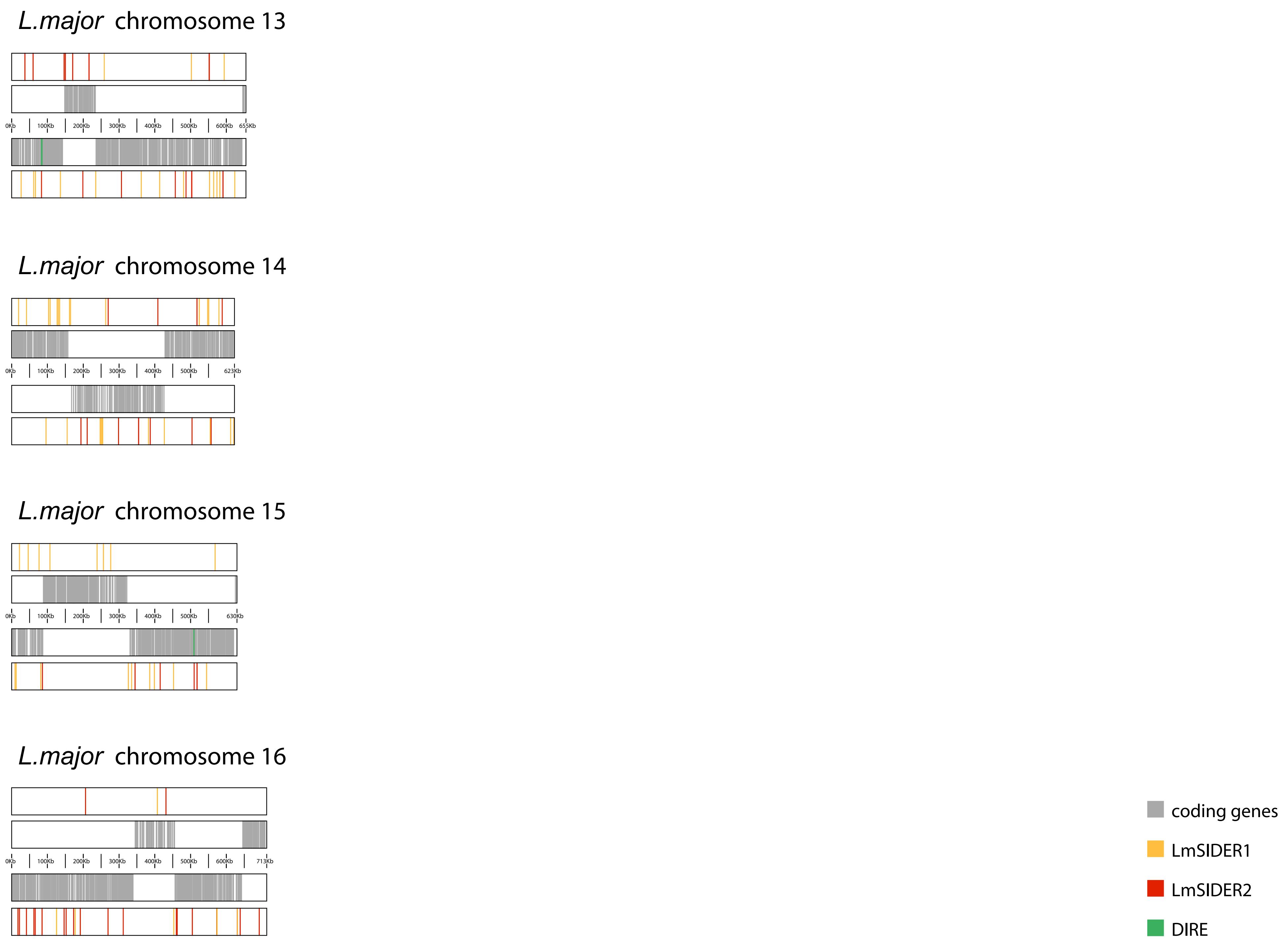
L.major chromosome 13
0Kb 100Kb 200Kb 300Kb 400Kb 500Kb 600Kb 655Kb
L.major chromosome 14
0Kb 100Kb 200Kb 300Kb 400Kb 500Kb 623Kb
L.major chromosome 15
0Kb 100Kb 200Kb 300Kb 400Kb 500Kb 630Kb
L.major chromosome 16
0Kb 100Kb 200Kb 300Kb 400Kb 500Kb 600Kb 713Kb
coding genes
LmSIDER1
LmSIDER2
DIRE

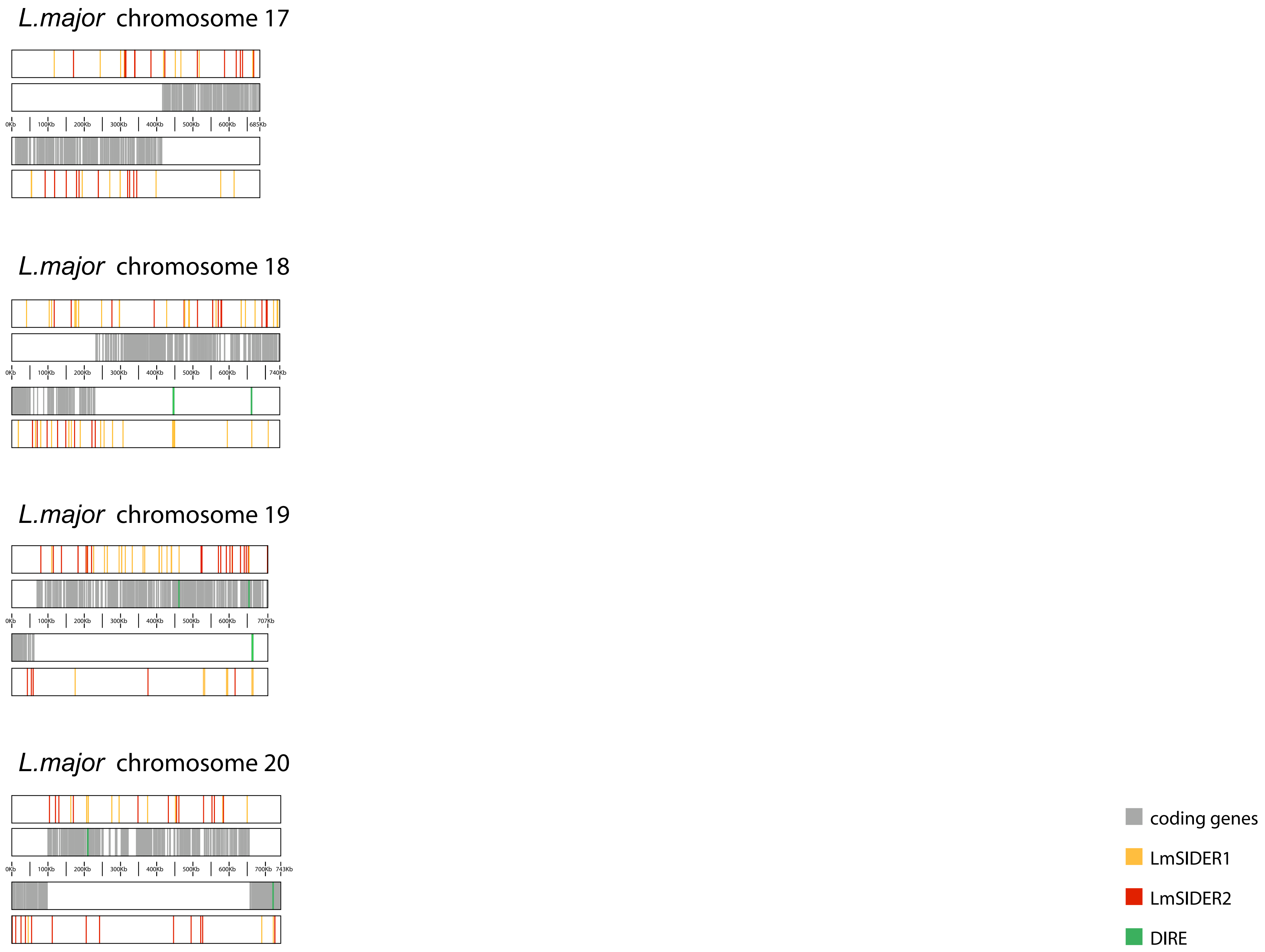

L.major chromosome 17
0Kb 100Kb 200Kb 300Kb 400Kb 500Kb 600Kb 685Kb
L.major chromosome 18
0Kb 100Kb 200Kb 300Kb 400Kb 500Kb 600Kb 740Kb
L.major chromosome 19
0Kb 100Kb 200Kb 300Kb 400Kb 500Kb 600Kb 707Kb
L.major chromosome 20
0Kb 100Kb 200Kb 300Kb 400Kb 500Kb 600Kb 700Kb 743Kb
coding genes
LmSIDER1
LmSIDER2
DIRE

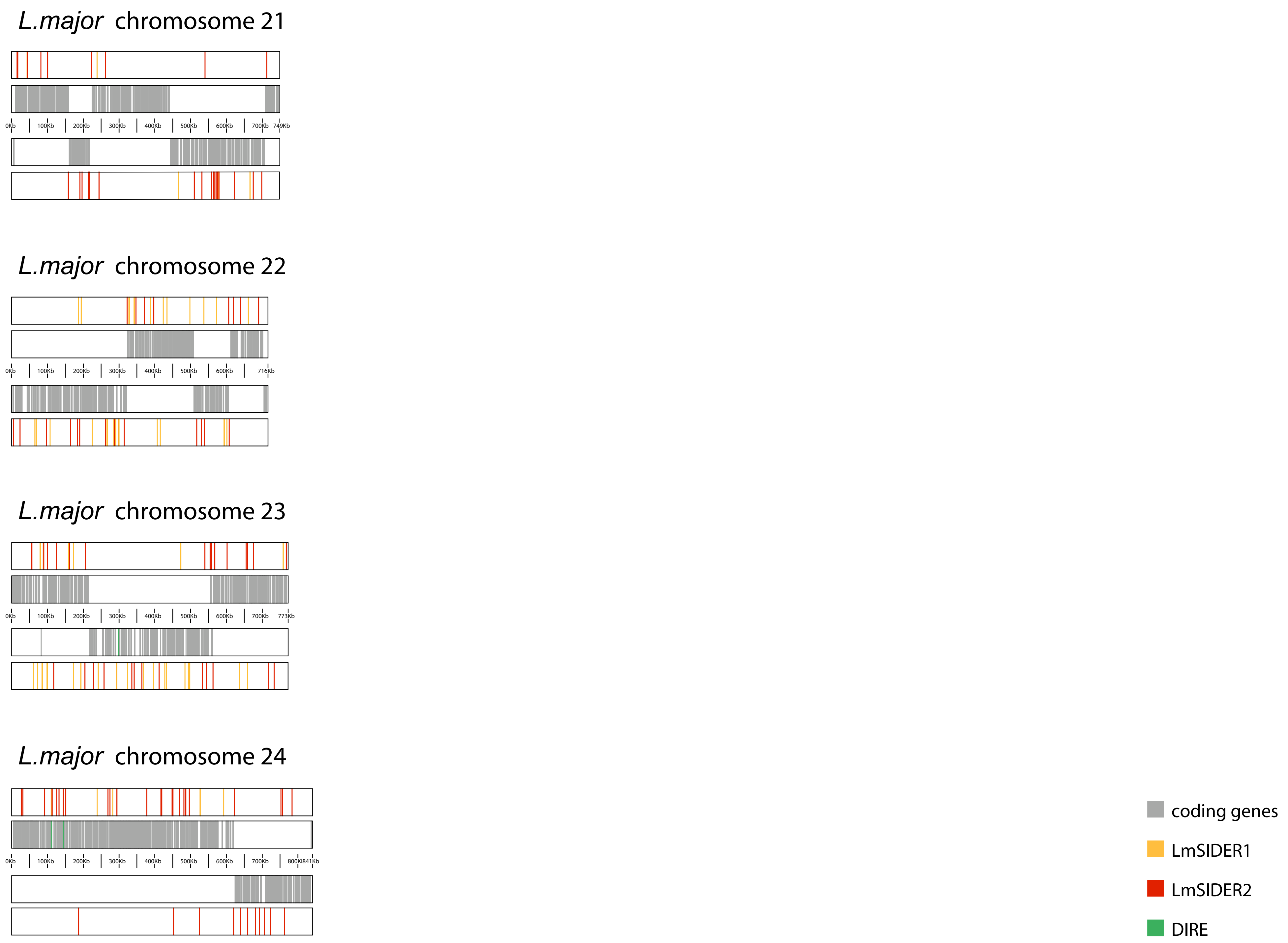
L.major chromosome 21
0Kb
100Kb
200Kb
300Kb
400Kb
500Kb
600Kb
700Kb
749Kb
L.major chromosome 22
0Kb
100Kb
200Kb
300Kb
400Kb
500Kb
600Kb
716Kb
L.major chromosome 23
0Kb
100Kb
200Kb
300Kb
400Kb
500Kb
600Kb
700Kb
773Kb
L.major chromosome 24
0Kb
100Kb
200Kb
300Kb
400Kb
500Kb
600Kb
700Kb
800Kb
841Kb
coding genes
LmSIDER1
LmSIDER2
DIRE

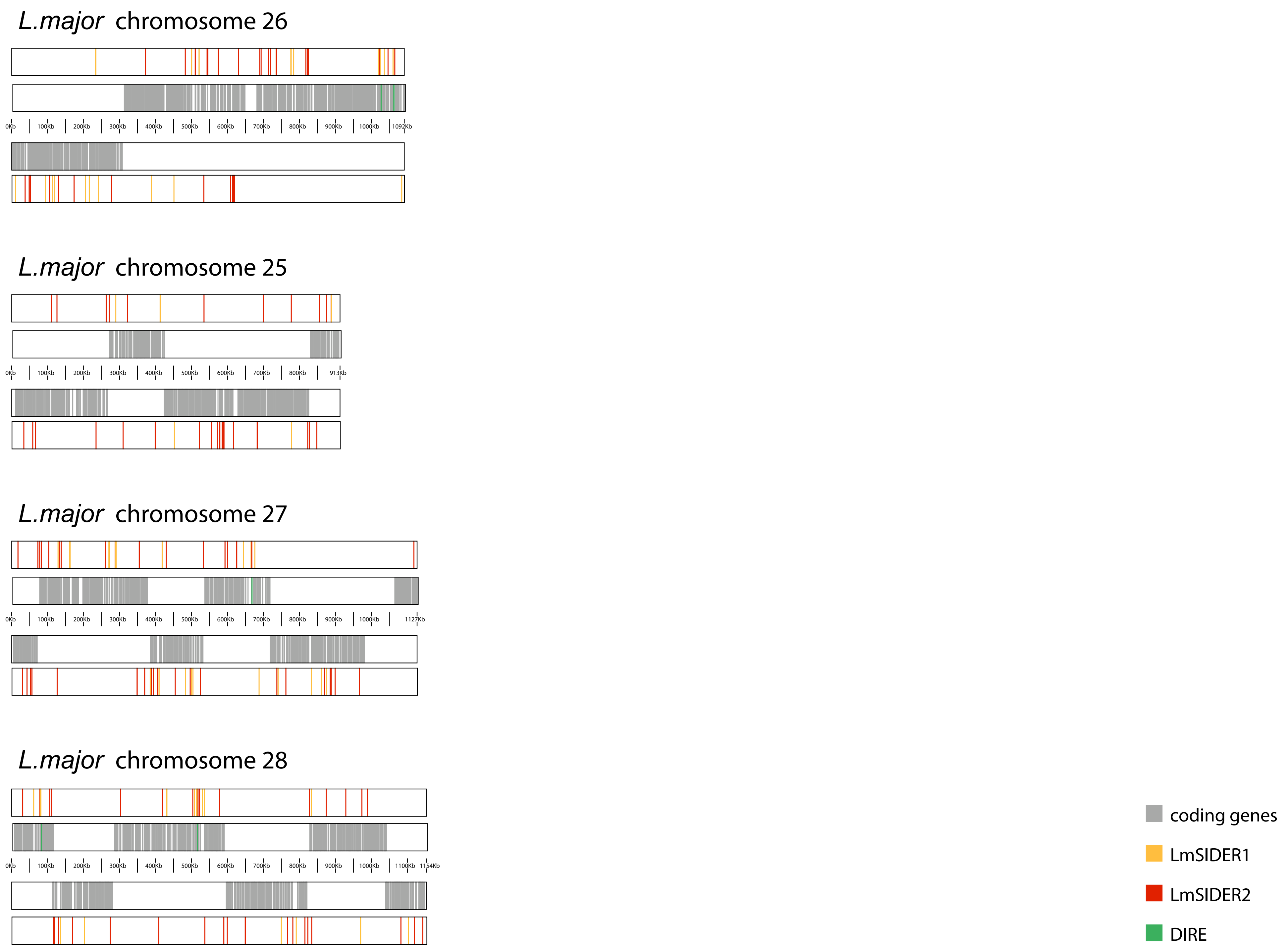
L.major chromosome 26
0Kb
100Kb
200Kb
300Kb
400Kb
500Kb
600Kb
700Kb
800Kb
900Kb
1000Kb
1092Kb
L.major chromosome 25
0Kb
100Kb
200Kb
300Kb
400Kb
500Kb
600Kb
700Kb
800Kb
913Kb
L.major chromosome 27
0Kb
100Kb
200Kb
300Kb
400Kb
500Kb
600Kb
700Kb
800Kb
900Kb
1000Kb
1127Kb
L.major chromosome 28
0Kb
100Kb
200Kb
300Kb
400Kb
500Kb
600Kb
700Kb
800Kb
900Kb
1000Kb
1100Kb
1154Kb
coding genes
LmSIDER1
LmSIDER2
DIRE

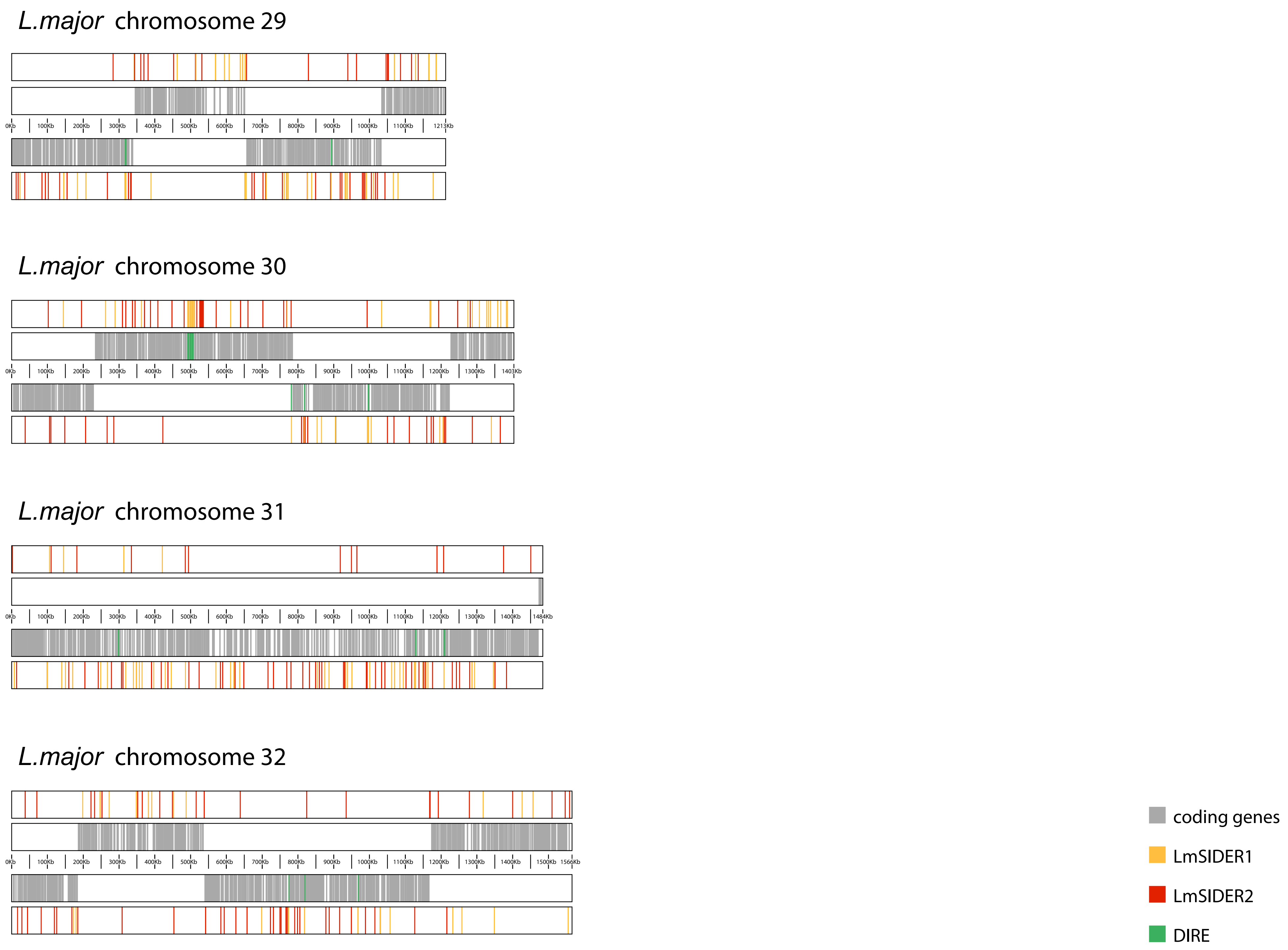

L.major chromosome 29
0Kb 100Kb 200Kb 300Kb 400Kb 500Kb 600Kb 700Kb 800Kb 900Kb 1000Kb 1100Kb 1213Kb
L.major chromosome 30
0Kb 100Kb 200Kb 300Kb 400Kb 500Kb 600Kb 700Kb 800Kb 900Kb 1000Kb 1100Kb 1200Kb 1300Kb 1403Kb
L.major chromosome 31
0Kb 100Kb 200Kb 300Kb 400Kb 500Kb 600Kb 700Kb 800Kb 900Kb 1000Kb 1100Kb 1200Kb 1300Kb 1400Kb 1484Kb
L.major chromosome 32
0Kb 100Kb 200Kb 300Kb 400Kb 500Kb 600Kb 700Kb 800Kb 900Kb 1000Kb 1100Kb 1200Kb 1300Kb 1400Kb 1500Kb 1566Kb
coding genes
LmSIDER1
LmSIDER2
DIRE

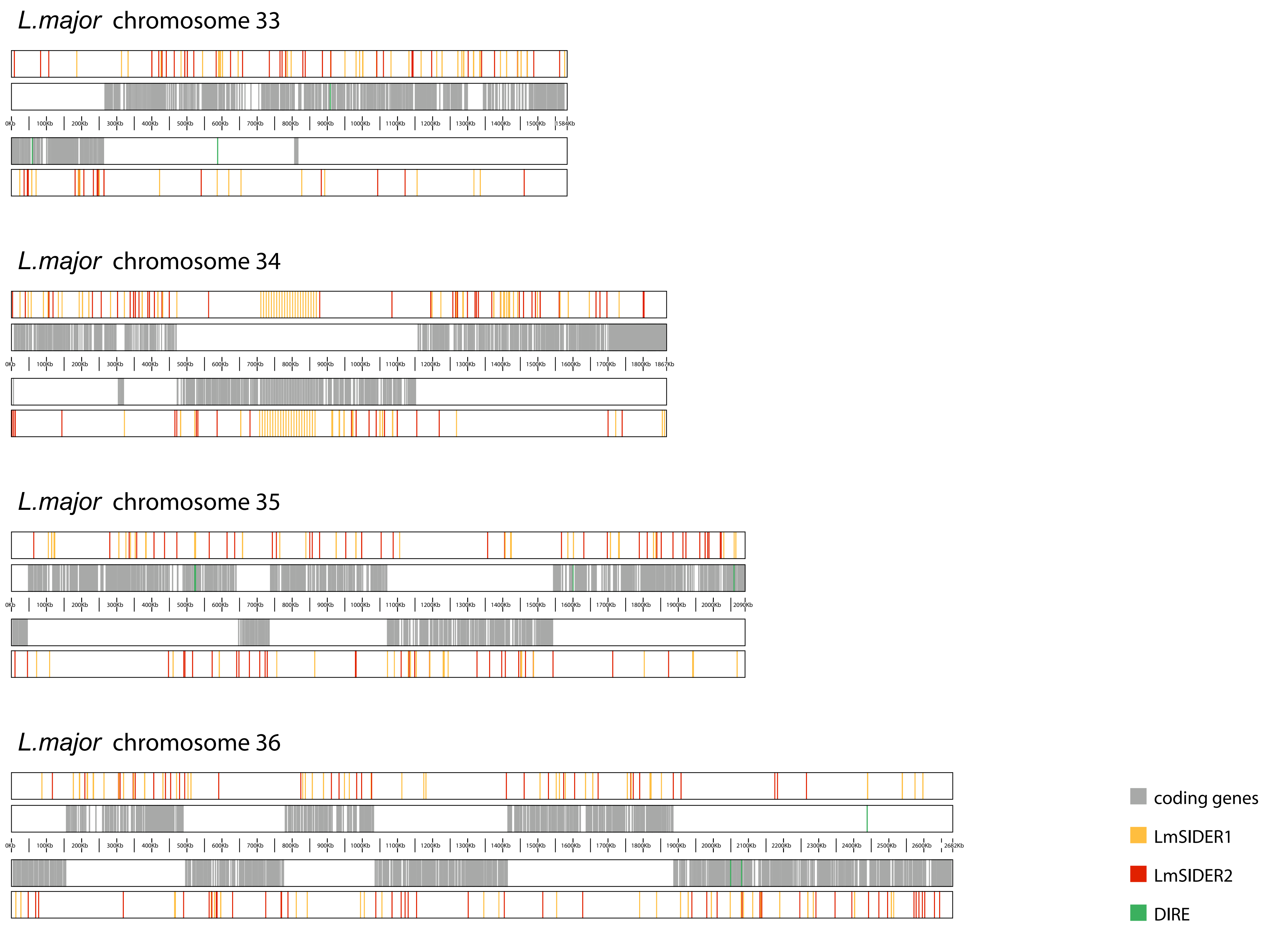

L.major chromosome 33
L.major chromosome 34
L.major chromosome 35
L.major chromosome 36
coding genes
LmSIDER1
LmSIDER2
DIRE
