# Supplementary material for: Members of a Large Retroposon Family Are Determinants of Post-Transcriptional Gene Expression in Leishmania
Source: PLoS Pathog. 2007 Sep 28;3(9):e136. doi: 10.1371/journal.ppat.0030136 (PMC2323293; doi:10.1371/journal.ppat.0030136)

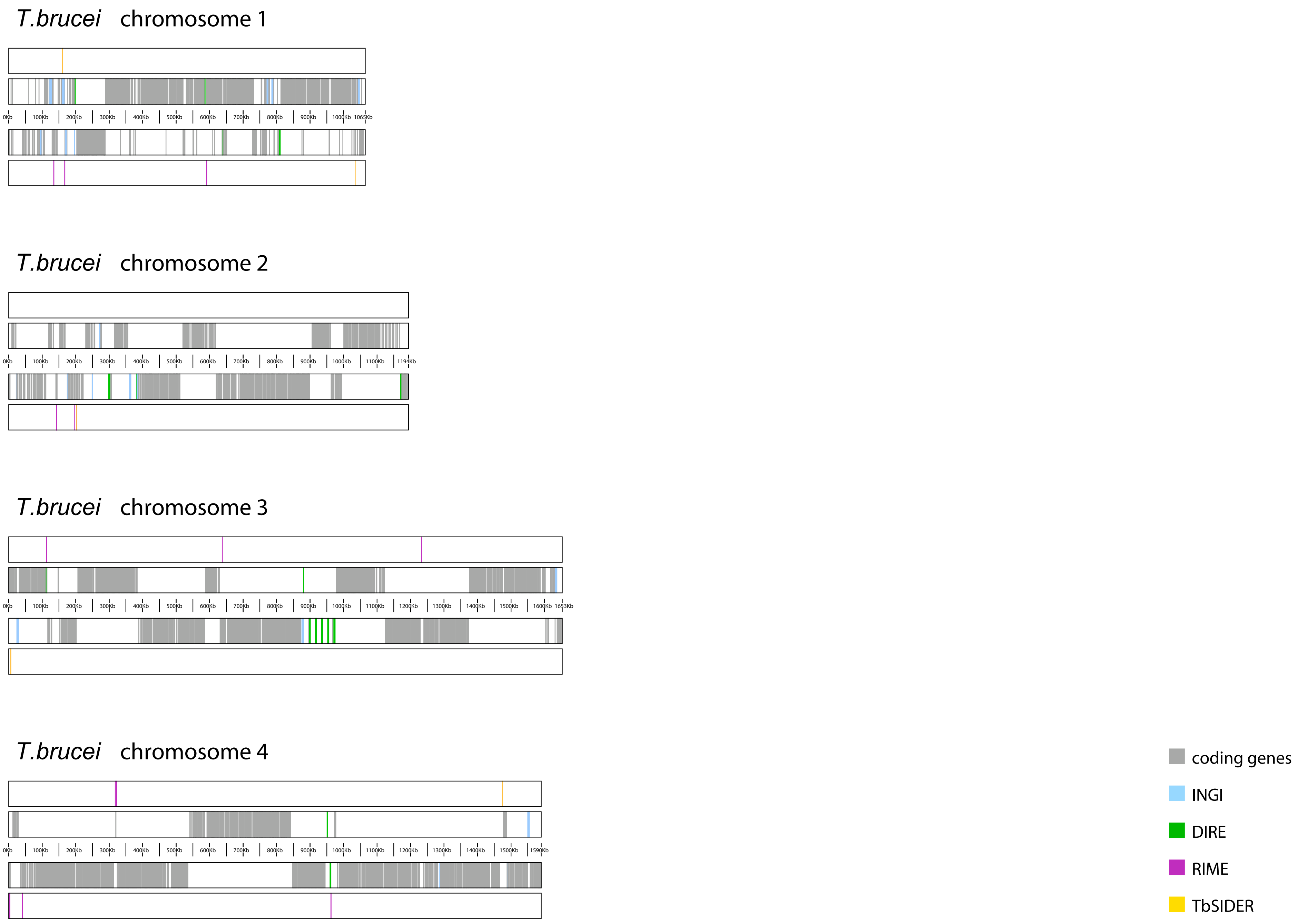
T.brucei chromosome 1
0Kb 100Kb 200Kb 300Kb 400Kb 500Kb 600Kb 700Kb 800Kb 900Kb 1000Kb 1065Kb
T.brucei chromosome 2
0Kb 100Kb 200Kb 300Kb 400Kb 500Kb 600Kb 700Kb 800Kb 900Kb 1000Kb 1100Kb 1194Kb
T.brucei chromosome 3
0Kb 100Kb 200Kb 300Kb 400Kb 500Kb 600Kb 700Kb 800Kb 900Kb 1000Kb 1100Kb 1200Kb 1300Kb 1400Kb 1500Kb 1600Kb 1653Kb
T.brucei chromosome 4
0Kb 100Kb 200Kb 300Kb 400Kb 500Kb 600Kb 700Kb 800Kb 900Kb 1000Kb 1100Kb 1200Kb 1300Kb 1400Kb 1500Kb 1590Kb
coding genes
INGI
DIRE
RIME
TbSIDER

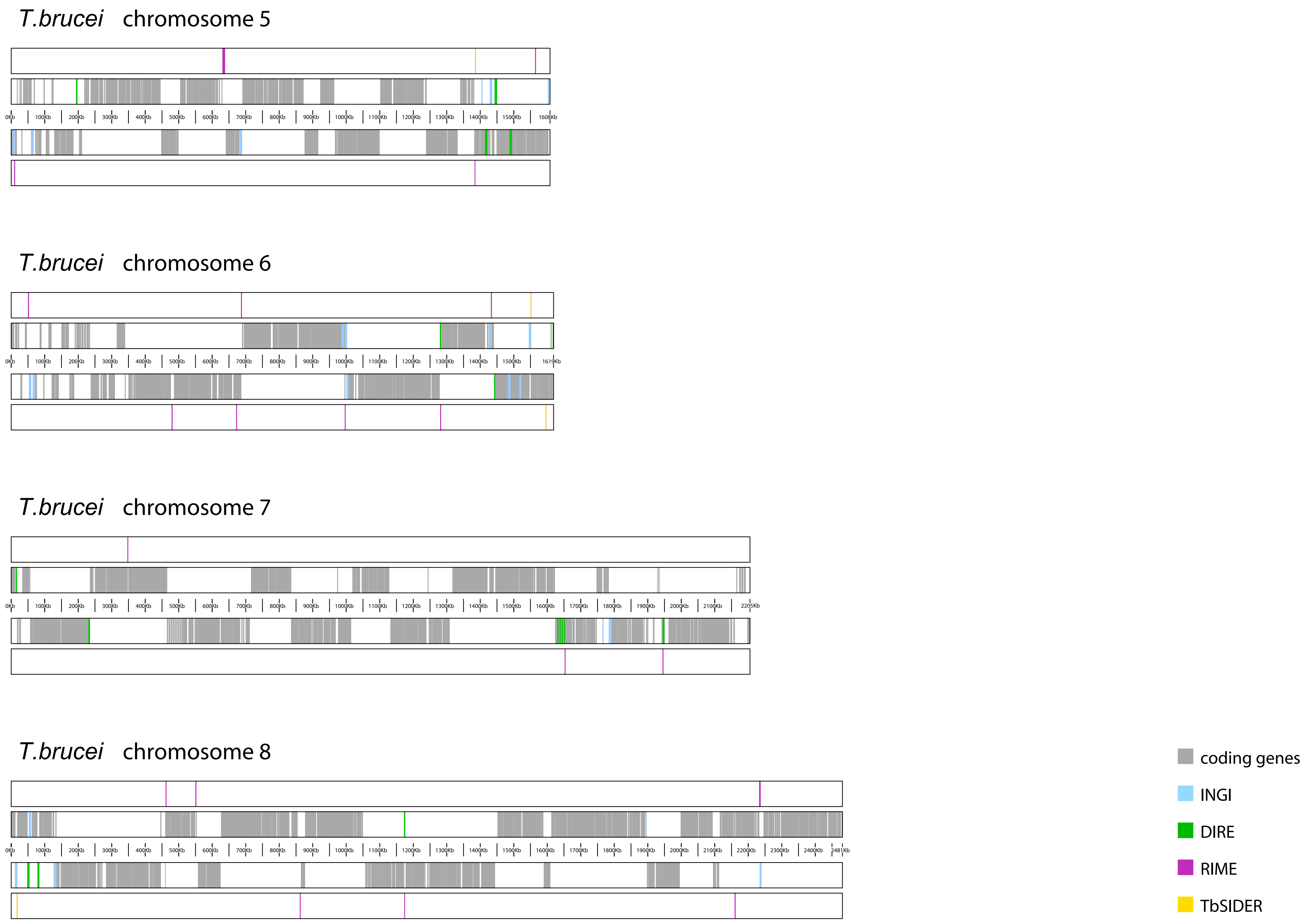
T.brucei chromosome 5
T.brucei chromosome 6
T.brucei chromosome 7
T.brucei chromosome 8
coding genes
INGI
DIRE
RIME
TbSIDER

T.brucei chromosome 9

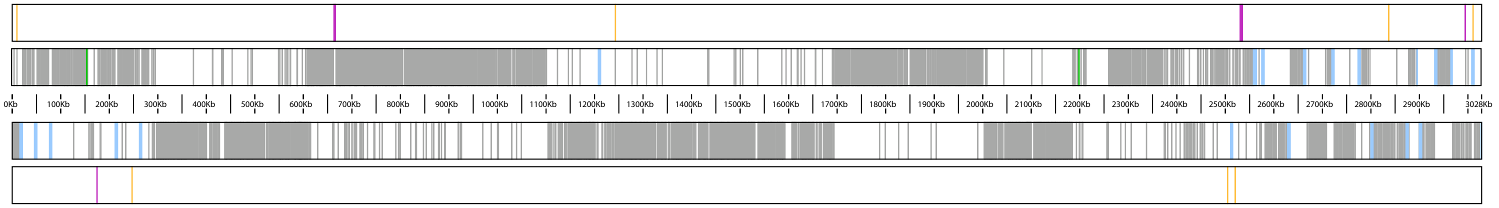

T.brucei chromosome 10

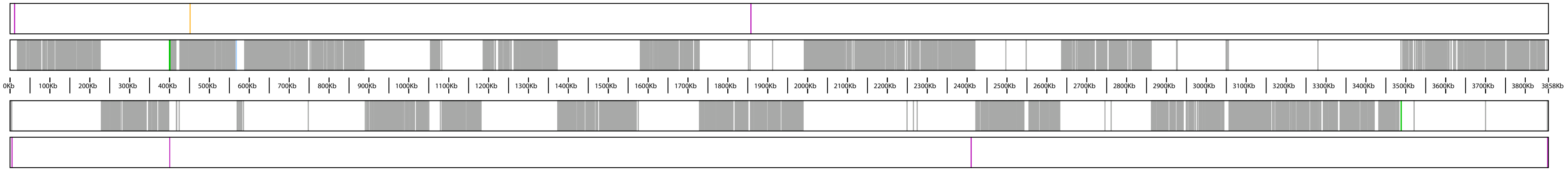

T.brucei chromosome 11 part 1

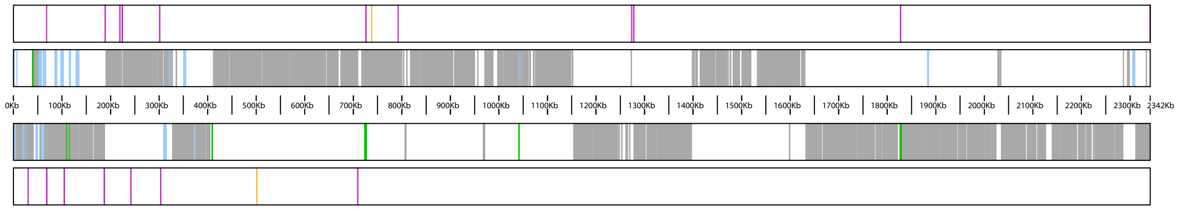

T.brucei chromosome 11 part 2

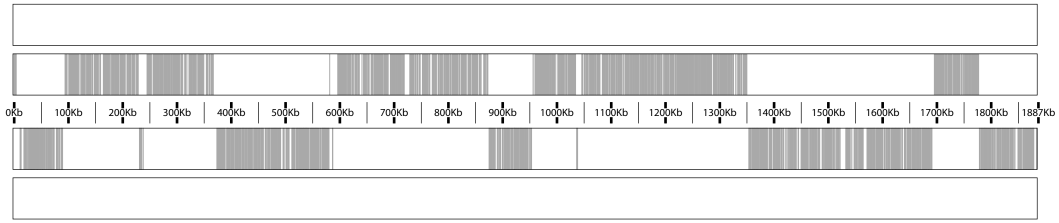

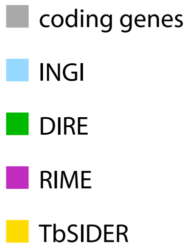

T.brucei chromosome 11 part 3

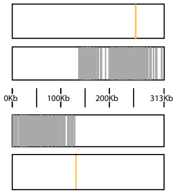

T.brucei chromosome 11 part 4

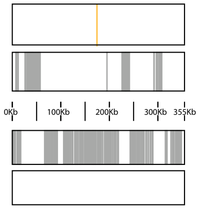

T.brucei chromosome 11 part 5

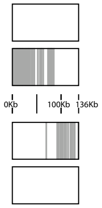

T.brucei chromosome 11 part 6

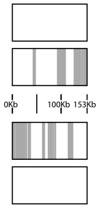

coding genes
INGI
DIRE
RIME
TbSIDER

T.brucei chromosome 11 part 7

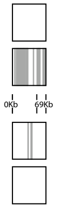

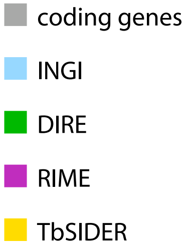

Supplement: Figure S4 — The central scale bars showing the size of the chromosomes (kb) separate features located on different strands. The position of protein-encoding genes and retroposons is indicated by vertical bars with the color code shown in the right margin. Protein-encoding genes and ingi and DIRE retroposons are shown in both central panels, while the upper or lower part of the schematic chromosomes indicate the position of RIME and TbSIDER retroposons. (856 KB DOC) [file ppat.0030136.sg004.pdf]
