# Supplementary material for: Members of a Large Retroposon Family Are Determinants of Post-Transcriptional Gene Expression in Leishmania
Source: PLoS Pathog. 2007 Sep 28;3(9):e136. doi: 10.1371/journal.ppat.0030136 (PMC2323293; doi:10.1371/journal.ppat.0030136)
Supplement: Table S1 — The chromosome localization (“chr”), genomic coordinates (“start” and “end”), strand localization (“str”), family (“fam”), and name (“name”) of the annotated LmSIDERs are indicated. The first column (“ID”) shows the name of each LmSIDER annotated in the database (version 4.0 of the assembly) hosted at The Institute for Genomic Research. The last column (“chr_size”) indicates the size of the chromosomes. (194 KB PDF) [file ppat.0030136.st001.pdf]

ID	chr	name	fam	str	start	end	chr_size
51.te00022	1	chr1_37.00281	1	-	39705	40532	268984
51.te00019	1	chr1_37.00289	1	+	260993	261261	268984
51.te00020	1	chr1_37.00290	1	+	266162	266340	268984
51.te00018	1	chr1_37.00279	2	+	24174	24821	268984
51.te00021	1	chr1_37.00280	2	-	35328	36036	268984
51.te00023	1	chr1_37.00282	2	-	54542	55178	268984
51.te00024	1	chr1_37.00283	2	+	73279	73917	268984
51.te00013	1	chr1_37.00284	2	-	111380	111734	268984
51.te00014	1	chr1_37.00285	2	-	126000	126649	268984
51.te00015	1	chr1_37.00286	2	+	133523	134013	268984
51.te00016	1	chr1_37.00287	2	+	201661	202234	268984
51.te00017	1	chr1_37.00288	2	-	226657	226940	268984
52.te00002	2	chr2_38.00226	1	-	135382	136457	355714
52.te00003	2	chr2_38.00224	2	+	17598	18236	355714
52.te00001	2	chr2_38.00225	2	-	102691	103323	355714
52.te00004	2	chr2_38.00227	2	-	201537	202251	355714
52.te00005	2	chr2_38.00228	2	-	254428	255004	355714
52.te00006	2	chr2_38.00229	2	-	264195	264734	355714
52.te00007	2	chr2_38.00230	2	+	314330	314887	355714
52.te00008	2	chr2_38.00231	2	+	343641	344192	355714
53.te00001	3	chr3_39.00295	2	+	171068	171643	384518
53.te00002	3	chr3_39.00296	2	+	225566	226067	384518
53.te00003	3	chr3_39.00297	2	+	236166	236600	384518
53.te00004	3	chr3_39.00298	2	+	252516	253083	384518
53.te00005	3	chr3_39.00299	2	+	258067	258658	384518
53.te00006	3	chr3_39.00300	2	-	314596	314966	384518
53.te00007	3	chr3_39.00301	2	+	343386	343807	384518
54.te00006	4	chr4_4.00423	1	-	336119	337040	472856
54.te00013	4	chr4_4.00416	2	+	7946	8423	472856
54.te00003	4	chr4_4.00417	2	-	19425	19898	472856
54.te00008	4	chr4_4.00418	2	+	38172	38588	472856
54.te00001	4	chr4_4.00419	2	+	108122	108581	472856
54.te00002	4	chr4_4.00420	2	-	181541	181927	472856
54.te00004	4	chr4_4.00421	2	-	290986	291500	472856
54.te00005	4	chr4_4.00422	2	-	335367	335810	472856
54.te00007	4	chr4_4.00424	2	+	367761	368203	472856
54.te00009	4	chr4_4.00425	2	-	396450	396896	472856
54.te00010	4	chr4_4.00426	2	-	399589	400014	472856
54.te00011	4	chr4_4.00427	2	-	432185	432800	472856
54.te00012	4	chr4_4.00428	2	+	443516	443993	472856
55.te00018	5	chr5_5.00339	1	+	90841	91072	465823
55.te00011	5	chr5_5.00348	1	+	413942	414427	465823
55.te00015	5	chr5_5.00351	1	+	444412	445160	465823
55.te00004	5	chr5_5.00335	2	+	21407	22009	465823
55.te00006	5	chr5_5.00336	2	+	30666	31304	465823
55.te00010	5	chr5_5.00337	2	+	40438	40917	465823
55.te00013	5	chr5_5.00338	2	+	44064	44598	465823
55.te00019	5	chr5_5.00340	2	+	91501	91943	465823
55.te00001	5	chr5_5.00341	2	-	133215	133682	465823
55.te00002	5	chr5_5.00342	2	+	146145	146639	465823
55.te00003	5	chr5_5.00343	2	+	150839	151245	465823

55.te00005	5	chr5_5.00344	2	+	222900	223499	465823
55.te00007	5	chr5_5.00345	2	+	355962	356336	465823
55.te00008	5	chr5_5.00346	2	-	384475	384939	465823
55.te00009	5	chr5_5.00347	2	+	386816	387303	465823
55.te00012	5	chr5_5.00349	2	-	437364	437911	465823
55.te00014	5	chr5_5.00350	2	-	440590	441136	465823
55.te00016	5	chr5_5.00352	2	+	446988	447532	465823
55.te00017	5	chr5_5.00353	2	+	464001	464509	465823
56.te00023	6	chr6_6.00295	1	-	6327	6767	513422
56.te00025	6	chr6_6.00297	1	-	7380	7589	513422
56.te00021	6	chr6_6.00299	1	-	57422	57917	513422
56.te00022	6	chr6_6.00300	1	-	61644	62048	513422
56.te00001	6	chr6_6.00301	1	+	101650	102456	513422
56.te00004	6	chr6_6.00304	1	-	178220	178503	513422
56.te00010	6	chr6_6.00309	1	+	392147	392407	513422
56.te00011	6	chr6_6.00310	1	-	438460	439271	513422
56.te00024	6	chr6_6.00296	2	+	6768	7379	513422
56.te00006	6	chr6_6.00298	2	-	19889	20502	513422
56.te00002	6	chr6_6.00302	2	+	137182	137798	513422
56.te00003	6	chr6_6.00303	2	+	176245	176890	513422
56.te00005	6	chr6_6.00305	2	+	185670	186275	513422
56.te00007	6	chr6_6.00306	2	+	212563	213170	513422
56.te00008	6	chr6_6.00307	2	+	240216	240827	513422
56.te00009	6	chr6_6.00308	2	-	283607	284031	513422
56.te00012	6	chr6_6.00311	2	+	440883	441360	513422
56.te00013	6	chr6_6.00312	2	+	445495	445986	513422
56.te00014	6	chr6_6.00313	2	+	450932	451439	513422
56.te00015	6	chr6_6.00314	2	+	457548	457967	513422
56.te00016	6	chr6_6.00315	2	+	482072	482591	513422
56.te00017	6	chr6_6.00316	2	-	490377	490884	513422
56.te00018	6	chr6_6.00317	2	-	501044	501619	513422
56.te00019	6	chr6_6.00318	2	-	502874	503338	513422
56.te00020	6	chr6_6.00319	2	+	507884	508405	513422
57.te00023	7	chr7_7.00415	1	+	209666	210003	596348
57.te00024	7	chr7_7.00416	1	+	212757	213679	596348
57.te00026	7	chr7_7.00418	1	+	251846	252218	596348
57.te00028	7	chr7_7.00419	1	+	304190	305151	596348
57.te00033	7	chr7_7.00423	1	+	444162	445147	596348
57.te00034	7	chr7_7.00424	1	+	453886	454444	596348
57.te00035	7	chr7_7.00425	1	+	460775	461791	596348
57.te00018	7	chr7_7.00406	2	+	13394	13844	596348
57.te00021	7	chr7_7.00407	2	+	20667	21214	596348
57.te00027	7	chr7_7.00408	2	+	27810	28293	596348
57.te00042	7	chr7_7.00409	2	+	59017	59505	596348
57.te00043	7	chr7_7.00410	2	-	60181	60831	596348
57.te00017	7	chr7_7.00411	2	-	105542	106179	596348
57.te00019	7	chr7_7.00412	2	-	167012	167515	596348
57.te00020	7	chr7_7.00413	2	-	193627	194180	596348
57.te00022	7	chr7_7.00414	2	-	208722	209299	596348
57.te00025	7	chr7_7.00417	2	-	246241	246922	596348
57.te00029	7	chr7_7.00420	2	+	305571	306180	596348
57.te00031	7	chr7_7.00421	2	-	339201	339725	596348

57.te00032	7	chr7_7.00422	2	+	370324	370865	596348
57.te00036	7	chr7_7.00426	2	+	506114	506704	596348
57.te00037	7	chr7_7.00427	2	+	531398	532034	596348
57.te00038	7	chr7_7.00428	2	+	553606	554095	596348
57.te00039	7	chr7_7.00429	2	+	562873	563495	596348
57.te00040	7	chr7_7.00430	2	+	566451	566952	596348
57.te00041	7	chr7_7.00431	2	+	571060	571650	596348
58.te00028	8	chr8_8.00369	1	+	46578	47000	559245
58.te00036	8	chr8_8.00370	1	+	54807	55176	559245
58.te00038	8	chr8_8.00371	1	+	66418	67263	559245
58.te00002	8	chr8_8.00373	1	+	104930	105246	559245
58.te00003	8	chr8_8.00374	1	+	106628	106929	559245
58.te00004	8	chr8_8.00375	1	+	192520	192788	559245
58.te00005	8	chr8_8.00376	1	+	192998	193635	559245
58.te00007	8	chr8_8.00378	1	+	288961	289511	559245
58.te00008	8	chr8_8.00379	1	+	294840	295389	559245
58.te00009	8	chr8_8.00380	1	+	300727	301211	559245
58.te00010	8	chr8_8.00381	1	+	306596	307146	559245
58.te00011	8	chr8_8.00382	1	+	312477	313067	559245
58.te00012	8	chr8_8.00383	1	+	318342	318892	559245
58.te00013	8	chr8_8.00384	1	+	324223	324773	559245
58.te00014	8	chr8_8.00385	1	+	330087	330637	559245
58.te00015	8	chr8_8.00386	1	+	335968	336518	559245
58.te00016	8	chr8_8.00387	1	+	341829	342379	559245
58.te00018	8	chr8_8.00388	1	+	347697	348247	559245
58.te00019	8	chr8_8.00389	1	+	359093	359412	559245
58.te00020	8	chr8_8.00390	1	+	363200	363589	559245
58.te00021	8	chr8_8.00391	1	+	368020	368409	559245
58.te00022	8	chr8_8.00392	1	+	372836	373225	559245
58.te00023	8	chr8_8.00393	1	+	377653	378042	559245
58.te00024	8	chr8_8.00394	1	+	382469	382858	559245
58.te00025	8	chr8_8.00395	1	+	387287	387676	559245
58.te00017	8	chr8_8.00368	2	+	34679	35263	559245
58.te00001	8	chr8_8.00372	2	+	104390	104921	559245
58.te00006	8	chr8_8.00377	2	+	281447	281905	559245
58.te00026	8	chr8_8.00396	2	-	418475	418943	559245
58.te00027	8	chr8_8.00397	2	-	446115	446693	559245
58.te00029	8	chr8_8.00398	2	-	483498	484064	559245
58.te00030	8	chr8_8.00399	2	+	486608	487167	559245
58.te00031	8	chr8_8.00400	2	-	491788	492360	559245
58.te00032	8	chr8_8.00401	2	+	495316	495867	559245
58.te00033	8	chr8_8.00402	2	+	530516	530969	559245
58.te00034	8	chr8_8.00403	2	+	534274	534713	559245
58.te00035	8	chr8_8.00404	2	+	541360	541889	559245
58.te00037	8	chr8_8.00405	2	+	553356	553919	559245
59.te00012	9	chr9_9.00352	1	+	41555	42555	573441
59.te00003	9	chr9_9.00356	1	-	166853	167203	573441
59.te00009	9	chr9_9.00361	1	+	252269	252848	573441
59.te00021	9	chr9_9.00372	1	-	552394	552842	573441
59.te00008	9	chr9_9.00351	2	+	24862	25362	573441
59.te00022	9	chr9_9.00353	2	+	90828	91404	573441
59.te00001	9	chr9_9.00354	2	+	110413	110990	573441

59.te00002	9	chr9_9.00355	2	+	129099	129512	573441
59.te00004	9	chr9_9.00357	2	-	199680	200237	573441
59.te00005	9	chr9_9.00358	2	-	217295	217687	573441
59.te00006	9	chr9_9.00359	2	+	224126	224524	573441
59.te00007	9	chr9_9.00360	2	+	234934	235158	573441
59.te00010	9	chr9_9.00362	2	-	406642	406881	573441
59.te00011	9	chr9_9.00363	2	+	413803	414373	573441
59.te00013	9	chr9_9.00364	2	-	425129	425732	573441
59.te00014	9	chr9_9.00365	2	-	430061	430634	573441
59.te00015	9	chr9_9.00366	2	-	443487	444062	573441
59.te00016	9	chr9_9.00367	2	-	498591	499063	573441
59.te00017	9	chr9_9.00368	2	+	506279	506606	573441
59.te00018	9	chr9_9.00369	2	-	514561	515018	573441
59.te00019	9	chr9_9.00370	2	+	519783	520193	573441
59.te00020	9	chr9_9.00371	2	-	530166	530646	573441
60.te00007	10	chr10_10.00446	1	+	31489	32354	570864
60.te00004	10	chr10_10.00453	1	-	211539	212466	570864
60.te00014	10	chr10_10.00460	1	-	483320	483648	570864
60.te00016	10	chr10_10.00462	1	-	514361	515209	570864
60.te00008	10	chr10_10.00445	2	-	2738	3314	570864
60.te00012	10	chr10_10.00447	2	+	35222	35746	570864
60.te00024	10	chr10_10.00448	2	+	72999	73626	570864
60.te00025	10	chr10_10.00449	2	+	91105	91729	570864
60.te00001	10	chr10_10.00450	2	+	161146	161602	570864
60.te00002	10	chr10_10.00451	2	+	184806	185324	570864
60.te00003	10	chr10_10.00452	2	+	193220	193696	570864
60.te00005	10	chr10_10.00454	2	+	241517	242154	570864
60.te00006	10	chr10_10.00455	2	+	267766	268422	570864
60.te00009	10	chr10_10.00456	2	-	341889	342537	570864
60.te00010	10	chr10_10.00457	2	-	344732	345368	570864
60.te00011	10	chr10_10.00458	2	-	347563	348199	570864
60.te00013	10	chr10_10.00459	2	+	457452	458075	570864
60.te00015	10	chr10_10.00461	2	-	511911	512475	570864
60.te00017	10	chr10_10.00463	2	+	529527	530109	570864
60.te00018	10	chr10_10.00464	2	+	536509	537091	570864
60.te00019	10	chr10_10.00465	2	+	541770	542337	570864
60.te00020	10	chr10_10.00466	2	+	543991	544553	570864
60.te00021	10	chr10_10.00467	2	+	547215	547781	570864
60.te00022	10	chr10_10.00468	2	+	554605	555179	570864
60.te00023	10	chr10_10.00469	2	+	566480	567052	570864
61.te00002	11	chr11_11.00331	1	+	103849	104197	582575
61.te00006	11	chr11_11.00334	1	+	159134	160051	582575
61.te00012	11	chr11_11.00340	1	+	194294	194777	582575
61.te00014	11	chr11_11.00341	1	+	208610	209624	582575
61.te00015	11	chr11_11.00342	1	+	252041	252571	582575
61.te00020	11	chr11_11.00347	1	+	357921	358884	582575
61.te00005	11	chr11_11.00327	2	+	15300	15608	582575
61.te00013	11	chr11_11.00328	2	+	20530	20838	582575
61.te00025	11	chr11_11.00329	2	+	40675	40984	582575
61.te00028	11	chr11_11.00330	2	+	55892	56460	582575
61.te00003	11	chr11_11.00332	2	-	120055	120617	582575
61.te00004	11	chr11_11.00333	2	-	123050	123575	582575

61.te00007	11	chr11_11.00335	2	+	162121	162664	582575
61.te00008	11	chr11_11.00336	2	+	163729	164248	582575
61.te00009	11	chr11_11.00337	2	+	177074	177616	582575
61.te00010	11	chr11_11.00338	2	+	188266	188797	582575
61.te00011	11	chr11_11.00339	2	+	192111	192658	582575
61.te00016	11	chr11_11.00343	2	+	255908	256604	582575
61.te00017	11	chr11_11.00344	2	+	283166	283807	582575
61.te00018	11	chr11_11.00345	2	+	294782	295409	582575
61.te00019	11	chr11_11.00346	2	+	337654	338230	582575
61.te00021	11	chr11_11.00348	2	+	364824	365283	582575
61.te00022	11	chr11_11.00349	2	+	380045	380502	582575
61.te00023	11	chr11_11.00350	2	+	389833	390296	582575
61.te00024	11	chr11_11.00351	2	+	404985	405444	582575
61.te00026	11	chr11_11.00352	2	+	463050	463441	582575
61.te00027	11	chr11_11.00353	2	+	540775	541410	582575
61.te00029	11	chr11_11.00354	2	-	569201	569824	582575
62.te00032	12	chr12_12.00387	1	+	65155	66023	675238
62.te00003	12	chr12_12.00389	1	+	109534	110215	675238
62.te00008	12	chr12_12.00394	1	-	230027	231087	675238
62.te00011	12	chr12_12.00397	1	-	284030	285093	675238
62.te00014	12	chr12_12.00400	1	+	308877	309358	675238
62.te00026	12	chr12_12.00410	1	+	565524	565993	675238
62.te00028	12	chr12_12.00412	1	+	603686	604571	675238
62.te00030	12	chr12_12.00414	1	+	618285	619143	675238
62.te00033	12	chr12_12.00416	1	+	655809	656358	675238
62.te00034	12	chr12_12.00417	1	+	657335	658190	675238
62.te00016	12	chr12_12.00385	2	+	34647	35191	675238
62.te00018	12	chr12_12.00386	2	-	39742	40325	675238
62.te00035	12	chr12_12.00388	2	+	91309	91788	675238
62.te00004	12	chr12_12.00390	2	-	110068	110637	675238
62.te00005	12	chr12_12.00391	2	+	124803	125312	675238
62.te00006	12	chr12_12.00392	2	+	137039	137603	675238
62.te00007	12	chr12_12.00393	2	-	179374	179949	675238
62.te00009	12	chr12_12.00395	2	-	269877	270472	675238
62.te00010	12	chr12_12.00396	2	-	282968	283286	675238
62.te00012	12	chr12_12.00398	2	+	289904	290381	675238
62.te00013	12	chr12_12.00399	2	+	301008	301372	675238
62.te00015	12	chr12_12.00401	2	+	327525	328105	675238
62.te00017	12	chr12_12.00402	2	+	386605	387165	675238
62.te00019	12	chr12_12.00403	2	+	394538	395099	675238
62.te00020	12	chr12_12.00404	2	+	407661	408232	675238
62.te00021	12	chr12_12.00405	2	+	447391	447951	675238
62.te00022	12	chr12_12.00406	2	+	462769	463329	675238
62.te00023	12	chr12_12.00407	2	+	491331	491891	675238
62.te00024	12	chr12_12.00408	2	+	526727	527287	675238
62.te00025	12	chr12_12.00409	2	+	535463	536023	675238
62.te00027	12	chr12_12.00411	2	+	584733	585309	675238
62.te00029	12	chr12_12.00413	2	+	604576	605141	675238
62.te00031	12	chr12_12.00415	2	+	633595	634106	675238
63.te00013	13	chr13_13.00427	1	+	36815	37116	654604
63.te00027	13	chr13_13.00428	1	+	59512	59826	654604
63.te00031	13	chr13_13.00431	1	-	82998	83696	654604

63.te00003	13	chr13_13.00433	1	+	146183	146877	654604
63.te00004	13	chr13_13.00434	1	+	149851	150291	654604
63.te00005	13	chr13_13.00435	1	+	170269	170968	654604
63.te00006	13	chr13_13.00436	1	-	198659	199070	654604
63.te00007	13	chr13_13.00437	1	+	215659	216533	654604
63.te00011	13	chr13_13.00440	1	-	306886	307244	654604
63.te00015	13	chr13_13.00443	1	-	457288	457593	654604
63.te00017	13	chr13_13.00445	1	-	486917	487319	654604
63.te00019	13	chr13_13.00447	1	-	502638	503702	654604
63.te00020	13	chr13_13.00448	1	+	551603	552664	654604
63.te00025	13	chr13_13.00453	1	-	590271	591151	654604
63.te00010	13	chr13_13.00426	2	-	26150	26702	654604
63.te00028	13	chr13_13.00429	2	-	61205	61738	654604
63.te00030	13	chr13_13.00430	2	-	66060	66523	654604
63.te00002	13	chr13_13.00432	2	-	135964	136513	654604
63.te00008	13	chr13_13.00438	2	-	234680	235210	654604
63.te00009	13	chr13_13.00439	2	+	258400	258883	654604
63.te00012	13	chr13_13.00441	2	-	361914	362564	654604
63.te00014	13	chr13_13.00442	2	-	413237	413782	654604
63.te00016	13	chr13_13.00444	2	-	480172	480786	654604
63.te00018	13	chr13_13.00446	2	+	502030	502626	654604
63.te00021	13	chr13_13.00449	2	-	552676	553272	654604
63.te00022	13	chr13_13.00450	2	-	564245	564844	654604
63.te00023	13	chr13_13.00451	2	-	573422	574052	654604
63.te00024	13	chr13_13.00452	2	-	581440	582026	654604
63.te00026	13	chr13_13.00454	2	+	593756	594334	654604
63.te00029	13	chr13_13.00455	2	-	623099	623715	654604
64.te00010	14	chr14_14.00368	1	-	193413	194045	622648
64.te00011	14	chr14_14.00369	1	-	211249	211462	622648
64.te00017	14	chr14_14.00375	1	+	269551	269981	622648
64.te00018	14	chr14_14.00376	1	-	298548	298976	622648
64.te00019	14	chr14_14.00377	1	-	354325	355123	622648
64.te00021	14	chr14_14.00379	1	-	387369	387650	622648
64.te00022	14	chr14_14.00380	1	+	408207	408660	622648
64.te00025	14	chr14_14.00382	1	-	504007	504497	622648
64.te00026	14	chr14_14.00383	1	+	517698	518134	622648
64.te00031	14	chr14_14.00388	1	-	557470	557778	622648
64.te00033	14	chr14_14.00390	1	+	588046	588444	622648
64.te00009	14	chr14_14.00357	2	+	19249	19731	622648
64.te00023	14	chr14_14.00358	2	+	41476	41955	622648
64.te00036	14	chr14_14.00359	2	-	95887	96425	622648
64.te00001	14	chr14_14.00360	2	+	102801	103366	622648
64.te00002	14	chr14_14.00361	2	+	107301	107837	622648
64.te00003	14	chr14_14.00362	2	+	126809	127372	622648
64.te00004	14	chr14_14.00363	2	+	130262	130820	622648
64.te00005	14	chr14_14.00364	2	+	133710	134259	622648
64.te00006	14	chr14_14.00365	2	-	154925	155447	622648
64.te00007	14	chr14_14.00366	2	+	161405	161772	622648
64.te00008	14	chr14_14.00367	2	+	164089	164625	622648
64.te00012	14	chr14_14.00370	2	-	247019	247613	622648
64.te00013	14	chr14_14.00371	2	-	249008	249602	622648
64.te00014	14	chr14_14.00372	2	-	251982	252578	622648

64.te00015	14	chr14_14.00373	2	-	253973	254541	622648
64.te00016	14	chr14_14.00374	2	+	262560	263145	622648
64.te00020	14	chr14_14.00378	2	-	382002	382620	622648
64.te00024	14	chr14_14.00381	2	-	426472	426942	622648
64.te00027	14	chr14_14.00384	2	+	523956	524546	622648
64.te00028	14	chr14_14.00385	2	+	547284	547845	622648
64.te00029	14	chr14_14.00386	2	+	550365	550952	622648
64.te00030	14	chr14_14.00387	2	-	554546	555140	622648
64.te00032	14	chr14_14.00389	2	+	578958	579466	622648
64.te00034	14	chr14_14.00391	2	-	611654	612233	622648
64.te00035	14	chr14_14.00392	2	-	620230	620799	622648
65.te00022	15	chr15_15.00419	1	-	85998	86373	629514
65.te00010	15	chr15_15.00426	1	-	344918	345130	629514
65.te00013	15	chr15_15.00429	1	-	414872	415252	629514
65.te00016	15	chr15_15.00431	1	-	509894	510253	629514
65.te00017	15	chr15_15.00432	1	-	517507	518416	629514
65.te00023	15	chr15_15.00413	2	-	9098	9693	629514
65.te00003	15	chr15_15.00414	2	-	12378	12983	629514
65.te00004	15	chr15_15.00415	2	+	21497	22088	629514
65.te00015	15	chr15_15.00416	2	+	46101	46649	629514
65.te00020	15	chr15_15.00417	2	+	76454	77032	629514
65.te00021	15	chr15_15.00418	2	-	81012	81590	629514
65.te00002	15	chr15_15.00420	2	+	106685	107238	629514
65.te00005	15	chr15_15.00421	2	+	238388	238964	629514
65.te00006	15	chr15_15.00422	2	+	256259	256750	629514
65.te00007	15	chr15_15.00423	2	+	276550	277130	629514
65.te00008	15	chr15_15.00424	2	-	325991	326265	629514
65.te00009	15	chr15_15.00425	2	-	335122	335408	629514
65.te00011	15	chr15_15.00427	2	-	385507	386114	629514
65.te00012	15	chr15_15.00428	2	-	398393	399010	629514
65.te00014	15	chr15_15.00430	2	-	452092	452696	629514
65.te00018	15	chr15_15.00433	2	-	544503	545137	629514
65.te00019	15	chr15_15.00434	2	+	568322	568955	629514
66.te00001	16	chr16_16.00326	1	-	125441	126249	712836
66.te00005	16	chr16_16.00330	1	-	177805	178269	712836
66.te00012	16	chr16_16.00335	1	+	390926	391573	712836
66.te00015	16	chr16_16.00337	1	-	436659	437333	712836
66.te00020	16	chr16_16.00342	1	-	557489	558355	712836
66.te00023	16	chr16_16.00344	1	-	610054	610497	712836
66.te00006	16	chr16_16.00320	2	-	17433	18097	712836
66.te00009	16	chr16_16.00321	2	-	21621	22251	712836
66.te00013	16	chr16_16.00322	2	-	41098	41751	712836
66.te00021	16	chr16_16.00323	2	-	61972	62583	712836
66.te00025	16	chr16_16.00324	2	-	65701	66341	712836
66.te00027	16	chr16_16.00325	2	-	85101	85779	712836
66.te00002	16	chr16_16.00327	2	-	146657	147214	712836
66.te00003	16	chr16_16.00328	2	-	152713	153043	712836
66.te00004	16	chr16_16.00329	2	-	173286	173766	712836
66.te00007	16	chr16_16.00331	2	-	192207	192555	712836
66.te00008	16	chr16_16.00332	2	+	205806	206388	712836
66.te00010	16	chr16_16.00333	2	-	268585	269168	712836
66.te00011	16	chr16_16.00334	2	-	311080	311615	712836

66.te00014	16	chr16_16.00336	2	+	415000	415533	712836
66.te00016	16	chr16_16.00338	2	-	443854	444341	712836
66.te00017	16	chr16_16.00339	2	-	446420	446764	712836
66.te00018	16	chr16_16.00340	2	-	488538	488902	712836
66.te00019	16	chr16_16.00341	2	-	556901	557467	712836
66.te00022	16	chr16_16.00343	2	-	609510	610043	712836
66.te00024	16	chr16_16.00345	2	-	617580	618167	712836
66.te00026	16	chr16_16.00346	2	-	670679	671267	712836
67.te00030	17	chr17_17.00370	1	-	29574	29686	684831
67.te00031	17	chr17_17.00371	1	-	29698	30377	684831
67.te00032	17	chr17_17.00372	1	-	30398	30626	684831
67.te00001	17	chr17_17.00374	1	+	66603	66891	684831
67.te00007	17	chr17_17.00380	1	-	175378	175990	684831
67.te00009	17	chr17_17.00382	1	+	225154	225555	684831
67.te00010	17	chr17_17.00383	1	-	251897	252086	684831
67.te00011	17	chr17_17.00384	1	-	280067	280971	684831
67.te00012	17	chr17_17.00385	1	+	281621	281861	684831
67.te00013	17	chr17_17.00386	1	+	291610	291860	684831
67.te00023	17	chr17_17.00396	1	-	375393	375617	684831
67.te00024	17	chr17_17.00397	1	+	395850	396680	684831
67.te00026	17	chr17_17.00399	1	+	428204	428665	684831
67.te00027	17	chr17_17.00400	1	+	443642	444080	684831
67.te00029	17	chr17_17.00402	1	+	494279	494621	684831
67.te00033	17	chr17_17.00403	1	-	553745	554048	684831
67.te00035	17	chr17_17.00405	1	-	590565	591033	684831
67.te00039	17	chr17_17.00409	1	+	642116	642577	684831
67.te00041	17	chr17_17.00373	2	-	41210	41915	684831
67.te00002	17	chr17_17.00375	2	-	67465	68122	684831
67.te00003	17	chr17_17.00376	2	-	131381	132033	684831
67.te00004	17	chr17_17.00377	2	+	151403	152085	684831
67.te00005	17	chr17_17.00378	2	-	159660	160329	684831
67.te00006	17	chr17_17.00379	2	-	166702	167362	684831
67.te00008	17	chr17_17.00381	2	-	219882	220569	684831
67.te00014	17	chr17_17.00387	2	+	293266	293770	684831
67.te00015	17	chr17_17.00388	2	+	296060	296575	684831
67.te00016	17	chr17_17.00389	2	+	332671	333258	684831
67.te00017	17	chr17_17.00390	2	+	344006	344582	684831
67.te00018	17	chr17_17.00391	2	+	349525	350100	684831
67.te00019	17	chr17_17.00392	2	-	354435	355018	684831
67.te00020	17	chr17_17.00393	2	+	356934	357536	684831
67.te00021	17	chr17_17.00394	2	+	357922	358491	684831
67.te00022	17	chr17_17.00395	2	-	362703	363272	684831
67.te00025	17	chr17_17.00398	2	+	399317	399891	684831
67.te00028	17	chr17_17.00401	2	+	488944	489535	684831
67.te00034	17	chr17_17.00404	2	+	564118	564681	684831
67.te00036	17	chr17_17.00406	2	+	596393	597050	684831
67.te00037	17	chr17_17.00407	2	+	607674	608225	684831
67.te00038	17	chr17_17.00408	2	+	613777	614383	684831
67.te00040	17	chr17_17.00410	2	+	644686	645249	684831
68.te00016	18	chr18_18.00475	1	-	19558	20417	739751
68.te00030	18	chr18_18.00476	1	+	42557	43000	739751
68.te00050	18	chr18_18.00478	1	-	67010	67489	739751

68.te00061	18	chr18_18.00480	1	-	81313	82172	739751
68.te00003	18	chr18_18.00482	1	+	105000	105306	739751
68.te00005	18	chr18_18.00484	1	-	111764	112026	739751
68.te00004	18	chr18_18.00483	1	+	111793	112012	739751
68.te00009	18	chr18_18.00488	1	-	159216	159620	739751
68.te00011	18	chr18_18.00490	1	-	166287	166718	739751
68.te00013	18	chr18_18.00492	1	+	175838	176072	739751
68.te00014	18	chr18_18.00493	1	+	176075	177060	739751
68.te00015	18	chr18_18.00494	1	+	177063	178036	739751
68.te00017	18	chr18_18.00495	1	-	181889	182091	739751
68.te00020	18	chr18_18.00498	1	-	238068	238586	739751
68.te00021	18	chr18_18.00499	1	+	240836	241305	739751
68.te00022	18	chr18_18.00500	1	-	247595	248007	739751
68.te00024	18	chr18_18.00502	1	-	270762	271349	739751
68.te00025	18	chr18_18.00503	1	-	273170	273838	739751
68.te00026	18	chr18_18.00504	1	-	273984	274174	739751
68.te00027	18	chr18_18.00505	1	+	292415	293357	739751
68.te00028	18	chr18_18.00506	1	-	302577	302846	739751
68.te00031	18	chr18_18.00508	1	+	422952	423163	739751
68.te00032	18	chr18_18.00509	1	-	439147	439927	739751
68.te00033	18	chr18_18.00510	1	-	439956	440523	739751
68.te00034	18	chr18_18.00511	1	-	443110	443479	739751
68.te00037	18	chr18_18.00514	1	+	472518	473458	739751
68.te00038	18	chr18_18.00515	1	+	483898	484270	739751
68.te00039	18	chr18_18.00516	1	+	485415	486150	739751
68.te00042	18	chr18_18.00519	1	+	550979	551431	739751
68.te00047	18	chr18_18.00523	1	-	582153	582412	739751
68.te00048	18	chr18_18.00524	1	+	620243	621164	739751
68.te00049	18	chr18_18.00525	1	+	632017	632484	739751
68.te00051	18	chr18_18.00526	1	-	649838	650196	739751
68.te00052	18	chr18_18.00527	1	+	658682	659362	739751
68.te00057	18	chr18_18.00531	1	-	695048	695504	739751
68.te00058	18	chr18_18.00532	1	+	709866	710322	739751
68.te00059	18	chr18_18.00533	1	+	719589	719949	739751
68.te00060	18	chr18_18.00534	1	+	721405	722121	739751
68.te00035	18	chr18_18.00512	1	-	444955	445252	739751
68.te00044	18	chr18_18.00477	2	-	58725	59293	739751
68.te00055	18	chr18_18.00479	2	-	71794	72352	739751
68.te00062	18	chr18_18.00481	2	-	99080	99640	739751
68.te00006	18	chr18_18.00485	2	+	118822	119380	739751
68.te00007	18	chr18_18.00486	2	-	128318	128598	739751
68.te00008	18	chr18_18.00487	2	-	150605	151221	739751
68.te00010	18	chr18_18.00489	2	+	165725	166279	739751
68.te00012	18	chr18_18.00491	2	-	175103	175421	739751
68.te00018	18	chr18_18.00496	2	-	214008	214562	739751
68.te00019	18	chr18_18.00497	2	-	223035	223584	739751
68.te00023	18	chr18_18.00501	2	+	269213	269747	739751
68.te00029	18	chr18_18.00507	2	+	388438	388768	739751
68.te00036	18	chr18_18.00513	2	+	470876	471461	739751
68.te00040	18	chr18_18.00517	2	+	507957	508551	739751
68.te00041	18	chr18_18.00518	2	+	541606	542115	739751
68.te00043	18	chr18_18.00520	2	+	557091	557705	739751

68.te00045	18	chr18_18.00521	2	+	564595	565191	739751
68.te00046	18	chr18_18.00522	2	+	566376	566910	739751
68.te00053	18	chr18_18.00528	2	+	677710	678253	739751
68.te00054	18	chr18_18.00529	2	+	689554	690104	739751
68.te00056	18	chr18_18.00530	2	+	692097	692700	739751
69.te00053	19	chr19_40.00369	1	+	109911	110931	706761
69.te00056	19	chr19_40.00372	1	-	174593	175517	706761
69.te00059	19	chr19_40.00375	1	+	207429	208643	706761
69.te00062	19	chr19_40.00378	1	+	225545	226414	706761
69.te00063	19	chr19_40.00379	1	+	255613	255818	706761
69.te00064	19	chr19_40.00380	1	+	263309	264016	706761
69.te00066	19	chr19_40.00382	1	+	302798	303784	706761
69.te00068	19	chr19_40.00384	1	+	332235	332514	706761
69.te00072	19	chr19_40.00388	1	+	406234	406636	706761
69.te00073	19	chr19_40.00389	1	+	406930	407217	706761
69.te00074	19	chr19_40.00390	1	+	413791	414244	706761
69.te00075	19	chr19_40.00391	1	+	428194	428477	706761
69.te00077	19	chr19_40.00392	1	+	439916	440318	706761
69.te00078	19	chr19_40.00393	1	+	441012	441693	706761
69.te00079	19	chr19_40.00394	1	+	461582	462327	706761
69.te00082	19	chr19_40.00397	1	-	529104	529416	706761
69.te00083	19	chr19_40.00398	1	-	532416	532767	706761
69.te00089	19	chr19_40.00402	1	-	592557	593386	706761
69.te00090	19	chr19_40.00403	1	-	595998	596225	706761
69.te00092	19	chr19_40.00405	1	+	607727	608543	706761
69.te00099	19	chr19_40.00412	1	+	654772	655588	706761
69.te00100	19	chr19_40.00413	1	-	662556	663371	706761
69.te00101	19	chr19_40.00414	1	-	665750	666192	706761
69.te00065	19	chr19_40.00381	1	+	296066	296465	706761
69.te00067	19	chr19_40.00383	1	+	313019	313410	706761
69.te00069	19	chr19_40.00385	1	+	362146	362537	706761
69.te00070	19	chr19_40.00386	1	+	366714	367103	706761
69.te00076	19	chr19_40.00365	2	-	42751	43331	706761
69.te00084	19	chr19_40.00366	2	-	53647	54225	706761
69.te00087	19	chr19_40.00367	2	-	58607	59190	706761
69.te00103	19	chr19_40.00368	2	+	79903	80475	706761
69.te00054	19	chr19_40.00370	2	+	114401	114994	706761
69.te00055	19	chr19_40.00371	2	+	136805	137406	706761
69.te00057	19	chr19_40.00373	2	+	182584	183193	706761
69.te00058	19	chr19_40.00374	2	+	204783	205286	706761
69.te00060	19	chr19_40.00376	2	+	208660	209227	706761
69.te00061	19	chr19_40.00377	2	+	219990	220486	706761
69.te00071	19	chr19_40.00387	2	-	375779	376219	706761
69.te00080	19	chr19_40.00395	2	+	522239	522591	706761
69.te00081	19	chr19_40.00396	2	+	524770	525079	706761
69.te00085	19	chr19_40.00399	2	+	570293	570570	706761
69.te00086	19	chr19_40.00400	2	+	576587	576946	706761
69.te00088	19	chr19_40.00401	2	+	591917	592521	706761
69.te00091	19	chr19_40.00404	2	+	602067	602633	706761
69.te00093	19	chr19_40.00406	2	+	608565	609148	706761
69.te00094	19	chr19_40.00407	2	-	616010	616684	706761
69.te00095	19	chr19_40.00408	2	+	631009	631597	706761

69.te00096	19	chr19_40.00409	2	+	641365	641950	706761
69.te00097	19	chr19_40.00410	2	+	646975	647530	706761
69.te00098	19	chr19_40.00411	2	+	652582	653163	706761
69.te00102	19	chr19_40.00415	2	+	705334	705859	706761
70.te00027	20	chr20_20.00403	1	-	46251	46704	742551
70.te00010	20	chr20_20.00409	1	+	157351	157867	742551
70.te00014	20	chr20_20.00412	1	+	201292	201762	742551
70.te00013	20	chr20_20.00413	1	+	205458	206309	742551
70.te00015	20	chr20_20.00415	1	+	268800	269651	742551
70.te00018	20	chr20_20.00416	1	+	289265	289735	742551
70.te00019	20	chr20_20.00417	1	+	293425	294298	742551
70.te00021	20	chr20_20.00419	1	+	359654	359898	742551
70.te00025	20	chr20_20.00422	1	+	436474	437308	742551
70.te00036	20	chr20_20.00431	1	+	561827	562229	742551
70.te00038	20	chr20_20.00433	1	+	622286	623153	742551
70.te00039	20	chr20_20.00434	1	-	662627	663297	742551
70.te00040	20	chr20_20.00435	1	-	664452	664827	742551
70.te00009	20	chr20_20.00399	2	-	2155	2728	742551
70.te00005	20	chr20_20.00400	2	-	11739	12316	742551
70.te00017	20	chr20_20.00401	2	-	26374	26959	742551
70.te00022	20	chr20_20.00402	2	-	38486	39069	742551
70.te00033	20	chr20_20.00404	2	-	55971	56180	742551
70.te00004	20	chr20_20.00405	2	+	104874	105475	742551
70.te00006	20	chr20_20.00406	2	-	112621	113218	742551
70.te00007	20	chr20_20.00407	2	+	121465	122050	742551
70.te00008	20	chr20_20.00408	2	+	130915	131467	742551
70.te00011	20	chr20_20.00410	2	+	164449	164995	742551
70.te00012	20	chr20_20.00411	2	-	199885	200314	742551
70.te00016	20	chr20_20.00414	2	-	235047	235639	742551
70.te00020	20	chr20_20.00418	2	+	333260	333653	742551
70.te00023	20	chr20_20.00420	2	+	416941	417433	742551
70.te00024	20	chr20_20.00421	2	-	431267	431864	742551
70.te00026	20	chr20_20.00423	2	+	439605	440167	742551
70.te00028	20	chr20_20.00424	2	+	445982	446560	742551
70.te00029	20	chr20_20.00425	2	-	479883	480362	742551
70.te00030	20	chr20_20.00426	2	-	506573	506919	742551
70.te00031	20	chr20_20.00427	2	-	511598	512160	742551
70.te00032	20	chr20_20.00428	2	+	514178	514691	742551
70.te00034	20	chr20_20.00429	2	+	532066	532532	742551
70.te00035	20	chr20_20.00430	2	+	538873	539340	742551
70.te00037	20	chr20_20.00432	2	+	563911	564433	742551
70.te00042	20	chr20_20.00437	2	-	668761	669319	742551
70.te00041	20	chr20_20.00436	2	-	668766	668989	742551
71.te00013	21	chr21_21.00347	1	+	42985	43731	749309
71.te00015	21	chr21_21.00363	1	-	394101	395047	749309
71.te00024	21	chr21_21.00372	1	-	506318	506778	749309
71.te00027	21	chr21_21.00375	1	-	566908	567805	749309
71.te00010	21	chr21_21.00357	1	+	238844	239435	749309
71.te00002	21	chr21_21.00345	2	+	15089	15577	749309
71.te00004	21	chr21_21.00346	2	+	17049	17442	749309
71.te00014	21	chr21_21.00348	2	+	43754	44266	749309
71.te00031	21	chr21_21.00349	2	+	81613	81836	749309

71.te00001	21	chr21_21.00350	2	+	100469	101065	749309
71.te00003	21	chr21_21.00351	2	-	159460	160039	749309
71.te00005	21	chr21_21.00352	2	-	191908	192445	749309
71.te00006	21	chr21_21.00353	2	-	197875	198382	749309
71.te00007	21	chr21_21.00354	2	-	215161	215434	749309
71.te00008	21	chr21_21.00355	2	-	219070	219602	749309
71.te00009	21	chr21_21.00356	2	+	223027	223557	749309
71.te00011	21	chr21_21.00358	2	-	245183	245792	749309
71.te00012	21	chr21_21.00359	2	+	262503	263177	749309
71.te00016	21	chr21_21.00360	2	+	277871	278405	749309
71.te00017	21	chr21_21.00361	2	-	301173	301740	749309
71.te00018	21	chr21_21.00362	2	+	302330	302784	749309
71.te00019	21	chr21_21.00364	2	-	438091	438722	749309
71.te00020	21	chr21_21.00365	2	-	459551	460062	749309
71.te00021	21	chr21_21.00366	2	+	467263	467764	749309
71.te00022	21	chr21_21.00367	2	-	487019	487587	749309
71.te00023	21	chr21_21.00368	2	-	492347	492937	749309
71.te00025	21	chr21_21.00369	2	-	495674	496186	749309
71.te00026	21	chr21_21.00370	2	-	499315	499730	749309
71.te00028	21	chr21_21.00371	2	-	502767	503270	749309
71.te00029	21	chr21_21.00373	2	-	507040	507683	749309
71.te00030	21	chr21_21.00374	2	-	550130	550839	749309
72.te00037	21	chr21_21.00376	2	-	576042	576651	749309
72.te00008	21	chr21_21.00377	2	-	599894	600447	749309
72.te00048	21	chr21_21.00378	2	+	613034	613593	749309
72.te00045	22	chr22_22.00421	1	-	64770	65469	716396
72.te00049	22	chr22_22.00423	1	-	69384	69970	716396
72.te00001	22	chr22_22.00425	1	-	107309	107797	716396
72.te00004	22	chr22_22.00428	1	+	186508	186858	716396
72.te00006	22	chr22_22.00430	1	+	194324	194569	716396
72.te00007	22	chr22_22.00431	1	-	225446	225908	716396
72.te00010	22	chr22_22.00433	1	-	266045	266524	716396
72.te00011	22	chr22_22.00434	1	-	267077	267986	716396
72.te00012	22	chr22_22.00435	1	-	285345	286323	716396
72.te00014	22	chr22_22.00437	1	-	290086	291063	716396
72.te00016	22	chr22_22.00439	1	-	299444	300432	716396
72.te00019	22	chr22_22.00442	1	+	327613	328077	716396
72.te00020	22	chr22_22.00443	1	+	329543	330005	716396
72.te00021	22	chr22_22.00444	1	+	342062	342955	716396
72.te00022	22	chr22_22.00445	1	+	343521	344093	716396
72.te00025	22	chr22_22.00448	1	+	387892	388610	716396
72.te00027	22	chr22_22.00450	1	-	407106	407278	716396
72.te00028	22	chr22_22.00451	1	-	415272	415661	716396
72.te00029	22	chr22_22.00452	1	+	423583	423913	716396
72.te00030	22	chr22_22.00453	1	+	434142	434391	716396
72.te00031	22	chr22_22.00454	1	+	498125	498388	716396
72.te00034	22	chr22_22.00457	1	+	537292	537743	716396
72.te00036	22	chr22_22.00459	1	+	572164	572406	716396
72.te00038	22	chr22_22.00460	1	-	593317	594162	716396
72.te00039	22	chr22_22.00461	1	-	594310	594551	716396
72.te00040	22	chr22_22.00462	1	-	601320	601764	716396
72.te00046	22	chr22_22.00467	1	+	661356	662231	716396

72.te00050	22	chr22_22.00419	2	-	5469	5991	716396
72.te00002	22	chr22_22.00420	2	-	23296	23866	716396
72.te00003	22	chr22_22.00422	2	-	68911	69374	716396
72.te00005	22	chr22_22.00424	2	-	97321	97922	716396
72.te00009	22	chr22_22.00426	2	-	164599	165040	716396
72.te00013	22	chr22_22.00427	2	-	183666	184278	716396
72.te00015	22	chr22_22.00429	2	-	190017	190612	716396
72.te00017	22	chr22_22.00432	2	-	262104	262675	716396
72.te00018	22	chr22_22.00436	2	-	287580	288152	716396
72.te00023	22	chr22_22.00438	2	-	298059	298655	716396
72.te00024	22	chr22_22.00440	2	-	314595	315136	716396
72.te00026	22	chr22_22.00441	2	+	322697	323240	716396
72.te00032	22	chr22_22.00446	2	+	347366	347960	716396
72.te00033	22	chr22_22.00447	2	+	370754	370964	716396
72.te00035	22	chr22_22.00449	2	+	397279	397766	716396
72.te00041	22	chr22_22.00455	2	-	517103	517676	716396
72.te00042	22	chr22_22.00456	2	-	529999	530621	716396
72.te00043	22	chr22_22.00458	2	-	538289	538904	716396
72.te00044	22	chr22_22.00463	2	+	606571	607008	716396
72.te00047	22	chr22_22.00464	2	-	607890	608213	716396
73.te00059	22	chr22_22.00465	2	+	620165	620612	716396
73.te00079	22	chr22_22.00466	2	+	639645	639949	716396
73.te00024	22	chr22_22.00468	2	+	690180	690751	716396
73.te00063	23	chr23_23.00324	1	-	61067	61566	772562
73.te00070	23	chr23_23.00325	1	-	72192	72429	772562
73.te00074	23	chr23_23.00326	1	+	79077	79334	772562
73.te00075	23	chr23_23.00327	1	+	79834	80684	772562
73.te00076	23	chr23_23.00328	1	-	84716	85540	772562
73.te00077	23	chr23_23.00329	1	-	86061	86318	772562
73.te00078	23	chr23_23.00330	1	+	87874	88031	772562
73.te00080	23	chr23_23.00332	1	-	98678	99532	772562
73.te00025	23	chr23_23.00334	1	-	100029	100212	772562
73.te00028	23	chr23_23.00337	1	+	158260	158407	772562
73.te00031	23	chr23_23.00340	1	-	173001	173500	772562
73.te00032	23	chr23_23.00341	1	-	193006	193829	772562
73.te00033	23	chr23_23.00342	1	-	193903	194087	772562
73.te00037	23	chr23_23.00343	1	-	199734	200550	772562
73.te00040	23	chr23_23.00346	1	-	251409	252159	772562
73.te00041	23	chr23_23.00347	1	-	281255	282086	772562
73.te00045	23	chr23_23.00351	1	-	325642	326465	772562
73.te00050	23	chr23_23.00356	1	+	430428	430868	772562
73.te00052	23	chr23_23.00358	1	-	451694	451990	772562
73.te00053	23	chr23_23.00359	1	-	457828	458124	772562
73.te00064	23	chr23_23.00368	1	-	577350	577968	772562
73.te00067	23	chr23_23.00371	1	-	601208	601620	772562
73.te00072	23	chr23_23.00375	1	+	666926	667910	772562
73.te00030	23	chr23_23.00339	1	+	172240	172590	772562
73.te00046	23	chr23_23.00352	1	-	354623	355141	772562
73.te00048	23	chr23_23.00354	1	-	385727	386283	772562
73.te00049	23	chr23_23.00355	1	-	390740	391299	772562
73.te00051	23	chr23_23.00357	1	-	442038	442570	772562
73.te00026	23	chr23_23.00323	2	+	56186	56791	772562

73.te00027	23	chr23_23.00331	2	+	89519	90051	772562
73.te00029	23	chr23_23.00333	2	+	100172	100701	772562
73.te00034	23	chr23_23.00335	2	-	117324	117920	772562
73.te00035	23	chr23_23.00336	2	+	124342	124924	772562
73.te00036	23	chr23_23.00338	2	+	161360	161928	772562
73.te00038	23	chr23_23.00344	2	-	215862	216500	772562
73.te00039	23	chr23_23.00345	2	-	249917	250427	772562
73.te00042	23	chr23_23.00348	2	-	293556	294152	772562
73.te00043	23	chr23_23.00349	2	-	300012	300633	772562
73.te00044	23	chr23_23.00350	2	-	321659	322237	772562
73.te00047	23	chr23_23.00353	2	-	369780	370338	772562
73.te00054	23	chr23_23.00360	2	-	472891	473480	772562
73.te00055	23	chr23_23.00361	2	+	480099	480534	772562
73.te00056	23	chr23_23.00362	2	-	484821	485393	772562
73.te00057	23	chr23_23.00363	2	+	494625	495201	772562
73.te00058	23	chr23_23.00364	2	+	498383	498817	772562
73.te00060	23	chr23_23.00365	2	-	504034	504603	772562
73.te00061	23	chr23_23.00366	2	+	508961	509437	772562
73.te00062	23	chr23_23.00367	2	+	543192	543769	772562
73.te00065	23	chr23_23.00369	2	+	596497	597082	772562
73.te00066	23	chr23_23.00370	2	+	600619	601200	772562
73.te00068	23	chr23_23.00372	2	+	617250	617803	772562
73.te00069	23	chr23_23.00373	2	-	627044	627625	772562
73.te00071	23	chr23_23.00374	2	-	642001	642584	772562
73.te00073	23	chr23_23.00376	2	+	675721	676287	772562
74.te00011	24	chr24_24.00471	1	+	110126	110812	840950
74.te00015	24	chr24_24.00475	1	+	143846	144172	840950
74.te00019	24	chr24_24.00479	1	+	238753	239008	840950
74.te00023	24	chr24_24.00482	1	+	282124	282381	840950
74.te00037	24	chr24_24.00495	1	+	526280	526683	840950
74.te00038	24	chr24_24.00496	1	+	526761	527419	840950
74.te00039	24	chr24_24.00497	1	+	591753	592168	840950
74.te00040	24	chr24_24.00498	1	+	592177	592903	840950
74.te00020	24	chr24_24.00468	2	+	26522	27071	840950
74.te00025	24	chr24_24.00469	2	+	30986	31534	840950
74.te00053	24	chr24_24.00470	2	+	92090	92551	840950
74.te00012	24	chr24_24.00472	2	+	112729	113337	840950
74.te00013	24	chr24_24.00473	2	+	125637	126194	840950
74.te00014	24	chr24_24.00474	2	+	132095	132549	840950
74.te00016	24	chr24_24.00476	2	+	144748	145186	840950
74.te00017	24	chr24_24.00477	2	+	150915	151326	840950
74.te00018	24	chr24_24.00478	2	-	186799	187326	840950
74.te00021	24	chr24_24.00480	2	+	269010	269498	840950
74.te00022	24	chr24_24.00481	2	+	274618	275143	840950
74.te00024	24	chr24_24.00483	2	+	293910	294561	840950
74.te00026	24	chr24_24.00484	2	+	377312	377910	840950
74.te00027	24	chr24_24.00485	2	+	416823	417316	840950
74.te00028	24	chr24_24.00486	2	+	419118	419746	840950
74.te00029	24	chr24_24.00487	2	+	448082	448605	840950
74.te00030	24	chr24_24.00488	2	+	450522	451041	840950
74.te00031	24	chr24_24.00489	2	-	452228	452814	840950
74.te00032	24	chr24_24.00490	2	+	469351	469819	840950

74.te00033	24	chr24_24.00491	2	+	481168	481707	840950
74.te00034	24	chr24_24.00492	2	+	486269	486813	840950
74.te00035	24	chr24_24.00493	2	+	496012	496624	840950
74.te00036	24	chr24_24.00494	2	-	524737	525394	840950
74.te00041	24	chr24_24.00499	2	-	619930	620558	840950
74.te00042	24	chr24_24.00500	2	+	622039	622536	840950
74.te00043	24	chr24_24.00501	2	-	639088	639749	840950
74.te00044	24	chr24_24.00502	2	-	659569	660077	840950
74.te00045	24	chr24_24.00503	2	-	681344	681923	840950
74.te00046	24	chr24_24.00504	2	-	692190	692744	840950
74.te00047	24	chr24_24.00505	2	-	706382	706928	840950
74.te00048	24	chr24_24.00506	2	-	723671	724244	840950
74.te00049	24	chr24_24.00507	2	+	752116	752672	840950
74.te00050	24	chr24_24.00508	2	+	756482	757052	840950
74.te00051	24	chr24_24.00509	2	-	762444	763011	840950
74.te00052	24	chr24_24.00510	2	+	783205	783829	840950
75.te00006	25	chr25_25.00550	1	+	289383	289611	912849
75.te00012	25	chr25_25.00554	1	+	412377	412954	912849
75.te00013	25	chr25_25.00555	1	-	451337	451769	912849
75.te00028	25	chr25_25.00568	1	-	777499	777947	912849
75.te00036	25	chr25_25.00575	1	+	889299	889766	912849
75.te00009	25	chr25_25.00542	2	-	32565	33145	912849
75.te00019	25	chr25_25.00543	2	-	57543	58121	912849
75.te00024	25	chr25_25.00544	2	-	65538	66118	912849
75.te00001	25	chr25_25.00545	2	+	109701	110256	912849
75.te00002	25	chr25_25.00546	2	+	125700	125982	912849
75.te00003	25	chr25_25.00547	2	-	233524	234097	912849
75.te00004	25	chr25_25.00548	2	+	262199	262762	912849
75.te00005	25	chr25_25.00549	2	+	270781	271285	912849
75.te00007	25	chr25_25.00551	2	-	308712	309295	912849
75.te00008	25	chr25_25.00552	2	+	321696	322299	912849
75.te00011	25	chr25_25.00553	2	-	398055	398583	912849
75.te00014	25	chr25_25.00556	2	-	520930	521535	912849
75.te00015	25	chr25_25.00557	2	+	534409	534839	912849
75.te00016	25	chr25_25.00558	2	-	554083	554691	912849
75.te00017	25	chr25_25.00559	2	-	570841	571407	912849
75.te00018	25	chr25_25.00560	2	-	577720	578313	912849
75.te00020	25	chr25_25.00561	2	-	584160	584747	912849
75.te00021	25	chr25_25.00562	2	-	586538	587102	912849
75.te00022	25	chr25_25.00563	2	-	589152	589720	912849
75.te00023	25	chr25_25.00564	2	-	615870	616470	912849
75.te00025	25	chr25_25.00565	2	-	681627	682004	912849
75.te00026	25	chr25_25.00566	2	+	699382	699752	912849
75.te00027	25	chr25_25.00567	2	+	776916	777484	912849
75.te00029	25	chr25_25.00569	2	-	822046	822625	912849
75.te00030	25	chr25_25.00570	2	-	827036	827255	912849
75.te00031	25	chr25_25.00571	2	-	847590	848198	912849
75.te00032	25	chr25_25.00572	2	+	855093	855699	912849
75.te00034	25	chr25_25.00573	2	+	875552	876172	912849
75.te00035	25	chr25_25.00574	2	+	887796	888416	912849
76.te00056	26	chr26_26.00515	1	-	9549	10013	1091579
76.te00111	26	chr26_26.00519	1	-	93040	93289	1091579

76.te00066	26	chr26_26.00521	1	-	112411	113020	1091579
76.te00067	26	chr26_26.00522	1	-	118791	118991	1091579
76.te00070	26	chr26_26.00525	1	-	204180	204417	1091579
76.te00073	26	chr26_26.00528	1	+	233711	234257	1091579
76.te00078	26	chr26_26.00532	1	-	387783	388154	1091579
76.te00079	26	chr26_26.00533	1	-	450333	450577	1091579
76.te00082	26	chr26_26.00535	1	+	500293	500732	1091579
76.te00084	26	chr26_26.00537	1	+	520607	521312	1091579
76.te00090	26	chr26_26.00542	1	+	574037	574502	1091579
76.te00101	26	chr26_26.00553	1	+	734771	734990	1091579
76.te00105	26	chr26_26.00557	1	+	775846	776072	1091579
76.te00106	26	chr26_26.00558	1	+	776440	777178	1091579
76.te00107	26	chr26_26.00559	1	+	783995	784136	1091579
76.te00057	26	chr26_26.00563	1	+	1019165	1019608	1091579
76.te00059	26	chr26_26.00565	1	+	1024261	1025024	1091579
76.te00060	26	chr26_26.00566	1	+	1035808	1036557	1091579
76.te00063	26	chr26_26.00568	1	+	1060081	1060859	1091579
76.te00065	26	chr26_26.00570	1	-	1083956	1084152	1091579
76.te00071	26	chr26_26.00526	1	-	214571	215234	1091579
76.te00072	26	chr26_26.00527	1	+	232689	233153	1091579
76.te00074	26	chr26_26.00529	1	-	240295	240702	1091579
76.te00076	26	chr26_26.00516	2	-	36182	36861	1091579
76.te00080	26	chr26_26.00517	2	-	47540	48216	1091579
76.te00085	26	chr26_26.00518	2	-	51644	52284	1091579
76.te00062	26	chr26_26.00520	2	-	104237	104830	1091579
76.te00068	26	chr26_26.00523	2	-	129712	130345	1091579
76.te00069	26	chr26_26.00524	2	-	172460	173037	1091579
76.te00075	26	chr26_26.00530	2	-	276370	276964	1091579
76.te00077	26	chr26_26.00531	2	+	372038	372655	1091579
76.te00081	26	chr26_26.00534	2	+	482333	482927	1091579
76.te00083	26	chr26_26.00536	2	+	510016	510614	1091579
76.te00086	26	chr26_26.00538	2	-	533205	533809	1091579
76.te00087	26	chr26_26.00539	2	+	542965	543551	1091579
76.te00088	26	chr26_26.00540	2	+	543960	544546	1091579
76.te00089	26	chr26_26.00541	2	+	544955	545540	1091579
76.te00091	26	chr26_26.00543	2	+	575213	575723	1091579
76.te00092	26	chr26_26.00544	2	-	607384	607859	1091579
76.te00093	26	chr26_26.00545	2	-	613267	613819	1091579
76.te00094	26	chr26_26.00546	2	-	615748	616300	1091579
76.te00095	26	chr26_26.00547	2	-	618229	618781	1091579
76.te00096	26	chr26_26.00548	2	+	630950	631459	1091579
76.te00097	26	chr26_26.00549	2	+	689562	690024	1091579
76.te00098	26	chr26_26.00550	2	+	693155	693570	1091579
76.te00099	26	chr26_26.00551	2	+	713813	714394	1091579
76.te00100	26	chr26_26.00552	2	+	719852	720426	1091579
76.te00102	26	chr26_26.00554	2	+	735343	735966	1091579
76.te00103	26	chr26_26.00555	2	+	736028	736426	1091579
76.te00104	26	chr26_26.00556	2	+	736587	736985	1091579
76.te00108	26	chr26_26.00560	2	+	817772	818374	1091579
76.te00109	26	chr26_26.00561	2	+	822213	822644	1091579
76.te00110	26	chr26_26.00562	2	+	824231	824503	1091579
76.te00058	26	chr26_26.00564	2	+	1022469	1023061	1091579

76.te00061	26	chr26_26.00567	2	+	1046028	1046589	1091579
76.te00064	26	chr26_26.00569	2	+	1065157	1065669	1091579
77.te00047	27	chr27_27.00499	1	+	128635	128913	1127087
77.te00050	27	chr27_27.00502	1	+	161319	161449	1127087
77.te00051	27	chr27_27.00503	1	+	161891	162620	1127087
77.te00054	27	chr27_27.00505	1	+	270031	270260	1127087
77.te00055	27	chr27_27.00506	1	+	272029	272624	1127087
77.te00057	27	chr27_27.00508	1	+	288073	288240	1127087
77.te00058	27	chr27_27.00509	1	+	290205	290727	1127087
77.te00063	27	chr27_27.00513	1	-	384129	384990	1127087
77.te00067	27	chr27_27.00517	1	-	408504	409366	1127087
77.te00068	27	chr27_27.00518	1	+	417877	418765	1127087
77.te00072	27	chr27_27.00521	1	-	482166	482615	1127087
77.te00074	27	chr27_27.00523	1	-	498650	499211	1127087
77.te00075	27	chr27_27.00524	1	-	502967	503418	1127087
77.te00083	27	chr27_27.00530	1	+	643720	644671	1127087
77.te00084	27	chr27_27.00531	1	+	665288	665623	1127087
77.te00085	27	chr27_27.00532	1	+	665799	666749	1127087
77.te00087	27	chr27_27.00534	1	+	675763	676263	1127087
77.te00088	27	chr27_27.00535	1	-	686797	687308	1127087
77.te00091	27	chr27_27.00537	1	-	739718	740165	1127087
77.te00095	27	chr27_27.00539	1	-	831826	832493	1127087
77.te00096	27	chr27_27.00540	1	-	860069	861001	1127087
77.te00098	27	chr27_27.00542	1	-	874714	875119	1127087
77.te00052	27	chr27_27.00489	2	+	16948	17548	1127087
77.te00059	27	chr27_27.00490	2	-	29424	30003	1127087
77.te00069	27	chr27_27.00491	2	-	41413	41998	1127087
77.te00076	27	chr27_27.00492	2	-	50871	51454	1127087
77.te00079	27	chr27_27.00493	2	-	55123	55692	1127087
77.te00089	27	chr27_27.00494	2	+	72540	73092	1127087
77.te00093	27	chr27_27.00495	2	+	77552	78119	1127087
77.te00094	27	chr27_27.00496	2	+	82664	83249	1127087
77.te00044	27	chr27_27.00497	2	+	102548	103133	1127087
77.te00046	27	chr27_27.00498	2	-	125368	125955	1127087
77.te00048	27	chr27_27.00500	2	+	132152	132745	1127087
77.te00049	27	chr27_27.00501	2	+	137387	137981	1127087
77.te00053	27	chr27_27.00504	2	+	259943	260543	1127087
77.te00056	27	chr27_27.00507	2	+	287506	288063	1127087
77.te00060	27	chr27_27.00510	2	-	347655	348281	1127087
77.te00061	27	chr27_27.00511	2	+	354161	354777	1127087
77.te00062	27	chr27_27.00512	2	-	368641	369263	1127087
77.te00064	27	chr27_27.00514	2	-	386930	387544	1127087
77.te00065	27	chr27_27.00515	2	-	392691	393305	1127087
77.te00066	27	chr27_27.00516	2	-	403952	404532	1127087
77.te00070	27	chr27_27.00519	2	+	429421	430027	1127087
77.te00071	27	chr27_27.00520	2	-	453657	454275	1127087
77.te00073	27	chr27_27.00522	2	-	495095	495734	1127087
77.te00077	27	chr27_27.00525	2	-	523789	524344	1127087
77.te00078	27	chr27_27.00526	2	+	533057	533446	1127087
77.te00080	27	chr27_27.00527	2	+	592862	593504	1127087
77.te00081	27	chr27_27.00528	2	+	600140	600777	1127087
77.te00082	27	chr27_27.00529	2	+	625513	626016	1127087

77.te00086	27	chr27_27.00533	2	+	667438	668030	1127087
77.te00090	27	chr27_27.00536	2	-	736319	736854	1127087
77.te00092	27	chr27_27.00538	2	-	761526	762107	1127087
77.te00097	27	chr27_27.00541	2	-	869595	870037	1127087
77.te00099	27	chr27_27.00543	2	-	884555	885143	1127087
77.te00100	27	chr27_27.00544	2	-	887429	887944	1127087
77.te00101	27	chr27_27.00545	2	-	898479	898861	1127087
77.te00102	27	chr27_27.00546	2	-	965955	966539	1127087
77.te00045	27	chr27_27.00547	2	+	1118098	1118625	1127087
78.te00053	28	chr28_28.00573	1	+	62691	62987	1153766
78.te00059	28	chr28_28.00575	1	+	80475	80726	1153766
78.te00061	28	chr28_28.00576	1	+	82493	82801	1153766
78.te00032	28	chr28_28.00582	1	-	136104	136558	1153766
78.te00034	28	chr28_28.00584	1	-	202632	203299	1153766
78.te00040	28	chr28_28.00589	1	+	432803	433577	1153766
78.te00042	28	chr28_28.00591	1	+	509249	509572	1153766
78.te00043	28	chr28_28.00592	1	+	515873	516184	1153766
78.te00044	28	chr28_28.00593	1	+	518877	519359	1153766
78.te00047	28	chr28_28.00596	1	+	531946	532390	1153766
78.te00048	28	chr28_28.00597	1	+	537554	538198	1153766
78.te00055	28	chr28_28.00603	1	-	750547	750951	1153766
78.te00060	28	chr28_28.00606	1	-	792725	793119	1153766
78.te00065	28	chr28_28.00610	1	+	835356	835745	1153766
78.te00025	28	chr28_28.00618	1	-	1095506	1096356	1153766
78.te00069	28	chr28_28.00614	1	-	964371	964834	1153766
78.te00037	28	chr28_28.00572	2	+	32007	32586	1153766
78.te00057	28	chr28_28.00574	2	+	79423	79998	1153766
78.te00023	28	chr28_28.00577	2	+	107173	107652	1153766
78.te00026	28	chr28_28.00578	2	+	112409	112975	1153766
78.te00029	28	chr28_28.00579	2	-	116057	116711	1153766
78.te00030	28	chr28_28.00580	2	-	119585	120151	1153766
78.te00031	28	chr28_28.00581	2	-	131204	131644	1153766
78.te00033	28	chr28_28.00583	2	-	170019	170664	1153766
78.te00035	28	chr28_28.00585	2	-	274936	275544	1153766
78.te00036	28	chr28_28.00586	2	+	303850	304426	1153766
78.te00038	28	chr28_28.00587	2	-	409732	410335	1153766
78.te00039	28	chr28_28.00588	2	+	421333	421974	1153766
78.te00041	28	chr28_28.00590	2	+	505079	505484	1153766
78.te00045	28	chr28_28.00594	2	+	519565	520160	1153766
78.te00046	28	chr28_28.00595	2	+	524031	524531	1153766
78.te00049	28	chr28_28.00598	2	-	538203	538312	1153766
78.te00050	28	chr28_28.00599	2	+	579547	580128	1153766
78.te00051	28	chr28_28.00600	2	-	590683	591203	1153766
78.te00052	28	chr28_28.00601	2	-	600188	600706	1153766
78.te00054	28	chr28_28.00602	2	-	650045	650752	1153766
78.te00056	28	chr28_28.00604	2	-	768043	768555	1153766
78.te00058	28	chr28_28.00605	2	-	783292	783823	1153766
78.te00062	28	chr28_28.00607	2	-	816888	817270	1153766
78.te00063	28	chr28_28.00608	2	-	824937	825284	1153766
78.te00064	28	chr28_28.00609	2	+	830514	830894	1153766
78.te00066	28	chr28_28.00611	2	-	835749	836167	1153766
78.te00067	28	chr28_28.00612	2	+	875903	876319	1153766

78.te00068	28	chr28_28.00613	2	+	924141	924688	1153766
78.te00070	28	chr28_28.00615	2	+	968433	969056	1153766
78.te00071	28	chr28_28.00616	2	+	984134	984752	1153766
78.te00024	28	chr28_28.00617	2	-	1074594	1075176	1153766
78.te00027	28	chr28_28.00619	2	-	1112644	1113222	1153766
78.te00028	28	chr28_28.00620	2	-	1135242	1135798	1153766
79.te00090	29	chr29_29.00526	1	-	22735	23642	1212674
79.te00085	29	chr29_29.00532	1	-	146486	147656	1212674
79.te00087	29	chr29_29.00534	1	-	185158	185435	1212674
79.te00089	29	chr29_29.00535	1	-	208571	209444	1212674
79.te00093	29	chr29_29.00538	1	-	317437	318635	1212674
79.te00098	29	chr29_29.00543	1	+	343122	344301	1212674
79.te00104	29	chr29_29.00548	1	-	390655	391009	1212674
79.te00106	29	chr29_29.00550	1	+	463666	464010	1212674
79.te00108	29	chr29_29.00552	1	+	515885	516226	1212674
79.te00110	29	chr29_29.00554	1	+	570315	570867	1212674
79.te00111	29	chr29_29.00555	1	+	570912	571832	1212674
79.te00112	29	chr29_29.00556	1	+	594614	595528	1212674
79.te00113	29	chr29_29.00557	1	+	607812	608542	1212674
79.te00114	29	chr29_29.00558	1	+	638779	639654	1212674
79.te00115	29	chr29_29.00559	1	+	645520	645569	1212674
79.te00116	29	chr29_29.00560	1	+	645888	646538	1212674
79.te00117	29	chr29_29.00561	1	-	652037	652400	1212674
79.te00118	29	chr29_29.00562	1	+	652666	652916	1212674
79.te00119	29	chr29_29.00563	1	-	652860	653353	1212674
79.te00120	29	chr29_29.00564	1	-	656137	656432	1212674
79.te00126	29	chr29_29.00570	1	-	712113	712354	1212674
79.te00128	29	chr29_29.00572	1	-	761735	762461	1212674
79.te00129	29	chr29_29.00573	1	-	767917	768372	1212674
79.te00130	29	chr29_29.00574	1	-	769716	769970	1212674
79.te00131	29	chr29_29.00575	1	-	772905	773652	1212674
79.te00133	29	chr29_29.00577	1	-	827395	827560	1212674
79.te00135	29	chr29_29.00579	1	-	838636	839010	1212674
79.te00139	29	chr29_29.00582	1	-	892802	893723	1212674
79.te00142	29	chr29_29.00585	1	-	931824	932265	1212674
79.te00143	29	chr29_29.00586	1	-	934876	935317	1212674
79.te00144	29	chr29_29.00587	1	-	938903	939342	1212674
79.te00152	29	chr29_29.00594	1	-	991378	992439	1212674
79.te00071	29	chr29_29.00603	1	-	1066763	1067487	1212674
79.te00072	29	chr29_29.00604	1	+	1069555	1070615	1212674
79.te00073	29	chr29_29.00605	1	-	1080048	1080277	1212674
79.te00076	29	chr29_29.00608	1	+	1128955	1129235	1212674
79.te00078	29	chr29_29.00610	1	+	1165407	1166098	1212674
79.te00079	29	chr29_29.00611	1	+	1166252	1167103	1212674
79.te00080	29	chr29_29.00612	1	-	1178109	1179014	1212674
79.te00081	29	chr29_29.00613	1	+	1185799	1186478	1212674
79.te00082	29	chr29_29.00614	1	+	1186632	1187485	1212674
79.te00094	29	chr29_29.00539	1	-	320399	321061	1212674
79.te00063	29	chr29_29.00596	1	-	1011124	1011691	1212674
79.te00083	29	chr29_29.00524	2	-	12312	12863	1212674
79.te00088	29	chr29_29.00525	2	-	17892	18440	1212674
79.te00101	29	chr29_29.00527	2	-	37663	38214	1212674

79.te00137	29	chr29_29.00528	2	-	85762	86372	1212674
79.te00146	29	chr29_29.00529	2	-	95240	95834	1212674
79.te00066	29	chr29_29.00530	2	-	103737	104098	1212674
79.te00084	29	chr29_29.00531	2	-	135233	135788	1212674
79.te00086	29	chr29_29.00533	2	-	156051	156387	1212674
79.te00091	29	chr29_29.00536	2	-	268700	269294	1212674
79.te00092	29	chr29_29.00537	2	+	284421	285016	1212674
79.te00095	29	chr29_29.00540	2	-	327576	328145	1212674
79.te00096	29	chr29_29.00541	2	-	333492	334053	1212674
79.te00097	29	chr29_29.00542	2	-	335234	335806	1212674
79.te00099	29	chr29_29.00544	2	+	344614	345185	1212674
79.te00100	29	chr29_29.00545	2	+	361775	362347	1212674
79.te00102	29	chr29_29.00546	2	+	370619	371224	1212674
79.te00103	29	chr29_29.00547	2	+	382180	382751	1212674
79.te00105	29	chr29_29.00549	2	+	453666	454290	1212674
79.te00107	29	chr29_29.00551	2	+	514601	514959	1212674
79.te00109	29	chr29_29.00553	2	+	532441	533049	1212674
79.te00121	29	chr29_29.00565	2	+	656433	657013	1212674
79.te00122	29	chr29_29.00566	2	-	671715	672275	1212674
79.te00123	29	chr29_29.00567	2	-	678174	678747	1212674
79.te00124	29	chr29_29.00568	2	-	702688	703207	1212674
79.te00125	29	chr29_29.00569	2	-	710059	710588	1212674
79.te00127	29	chr29_29.00571	2	-	756805	757396	1212674
79.te00132	29	chr29_29.00576	2	-	826427	827075	1212674
79.te00134	29	chr29_29.00578	2	+	829316	829910	1212674
79.te00136	29	chr29_29.00580	2	-	849516	850071	1212674
79.te00138	29	chr29_29.00581	2	-	891384	892004	1212674
79.te00140	29	chr29_29.00583	2	-	918251	918869	1212674
79.te00141	29	chr29_29.00584	2	-	923029	923642	1212674
79.te00145	29	chr29_29.00588	2	+	939439	940067	1212674
79.te00147	29	chr29_29.00589	2	-	945756	946275	1212674
79.te00148	29	chr29_29.00590	2	+	963645	964204	1212674
79.te00149	29	chr29_29.00591	2	-	980267	980861	1212674
79.te00150	29	chr29_29.00592	2	-	983797	984406	1212674
79.te00151	29	chr29_29.00593	2	-	987303	987884	1212674
79.te00062	29	chr29_29.00595	2	-	1005370	1005955	1212674
79.te00064	29	chr29_29.00597	2	-	1016929	1017515	1212674
79.te00065	29	chr29_29.00598	2	-	1022377	1022965	1212674
79.te00067	29	chr29_29.00599	2	-	1043476	1044054	1212674
79.te00068	29	chr29_29.00600	2	+	1046352	1046813	1212674
79.te00069	29	chr29_29.00601	2	+	1050298	1050899	1212674
79.te00070	29	chr29_29.00602	2	+	1052900	1053496	1212674
79.te00074	29	chr29_29.00606	2	+	1086672	1087252	1212674
79.te00075	29	chr29_29.00607	2	+	1117523	1118100	1212674
79.te00077	29	chr29_29.00609	2	+	1136121	1136707	1212674
80.te00100	30	chr30_30.00560	1	+	144176	144472	1403454
80.te00104	30	chr30_30.00564	1	+	262069	262478	1403454
80.te00107	30	chr30_30.00567	1	+	288571	289449	1403454
80.te00112	30	chr30_30.00572	1	+	362275	362727	1403454
80.te00120	30	chr30_30.00579	1	+	491942	492374	1403454
80.te00121	30	chr30_30.00580	1	+	494743	495603	1403454
80.te00122	30	chr30_30.00581	1	+	498312	498972	1403454

80.te00123	30	chr30_30.00582	1	+	501112	501972	1403454
80.te00124	30	chr30_30.00583	1	+	504668	505192	1403454
80.te00125	30	chr30_30.00584	1	+	507468	508333	1403454
80.te00126	30	chr30_30.00585	1	+	510902	511226	1403454
80.te00142	30	chr30_30.00601	1	+	769261	769454	1403454
80.te00144	30	chr30_30.00603	1	-	781691	782055	1403454
80.te00147	30	chr30_30.00606	1	-	816678	817707	1403454
80.te00150	30	chr30_30.00609	1	-	853357	853780	1403454
80.te00151	30	chr30_30.00610	1	-	865375	865861	1403454
80.te00152	30	chr30_30.00611	1	-	904417	904816	1403454
80.te00155	30	chr30_30.00614	1	-	994066	994287	1403454
80.te00156	30	chr30_30.00615	1	-	994972	995868	1403454
80.te00157	30	chr30_30.00616	1	-	997075	997420	1403454
80.te00067	30	chr30_30.00617	1	-	1004533	1004916	1403454
80.te00069	30	chr30_30.00618	1	+	1033515	1033981	1403454
80.te00076	30	chr30_30.00623	1	+	1168476	1168825	1403454
80.te00077	30	chr30_30.00624	1	+	1171078	1171725	1403454
80.te00081	30	chr30_30.00628	1	-	1196107	1196349	1403454
80.te00082	30	chr30_30.00629	1	-	1205138	1205768	1403454
80.te00086	30	chr30_30.00633	1	+	1274205	1274974	1403454
80.te00088	30	chr30_30.00635	1	+	1286866	1287085	1403454
80.te00090	30	chr30_30.00637	1	+	1306612	1306846	1403454
80.te00091	30	chr30_30.00638	1	+	1327198	1327841	1403454
80.te00092	30	chr30_30.00639	1	+	1332494	1332978	1403454
80.te00093	30	chr30_30.00640	1	+	1337632	1338108	1403454
80.te00094	30	chr30_30.00641	1	-	1339957	1340608	1403454
80.te00095	30	chr30_30.00642	1	+	1357803	1358594	1403454
80.te00097	30	chr30_30.00644	1	+	1366276	1366730	1403454
80.te00098	30	chr30_30.00645	1	+	1382151	1382498	1403454
80.te00099	30	chr30_30.00646	1	+	1384752	1385457	1403454
80.te00136	30	chr30_30.00595	1	+	611772	612262	1403454
80.te00114	30	chr30_30.00556	2	-	37474	38083	1403454
80.te00068	30	chr30_30.00557	2	+	101877	102304	1403454
80.te00071	30	chr30_30.00558	2	-	105613	106191	1403454
80.te00073	30	chr30_30.00559	2	-	109200	109778	1403454
80.te00101	30	chr30_30.00561	2	-	148660	148807	1403454
80.te00102	30	chr30_30.00562	2	+	194917	195520	1403454
80.te00103	30	chr30_30.00563	2	-	206224	206817	1403454
80.te00105	30	chr30_30.00565	2	-	266439	267060	1403454
80.te00106	30	chr30_30.00566	2	-	284947	285568	1403454
80.te00108	30	chr30_30.00568	2	+	309208	309817	1403454
80.te00109	30	chr30_30.00569	2	+	318694	318906	1403454
80.te00110	30	chr30_30.00570	2	+	337323	337963	1403454
80.te00111	30	chr30_30.00571	2	+	344565	345157	1403454
80.te00113	30	chr30_30.00573	2	+	370953	371606	1403454
80.te00115	30	chr30_30.00574	2	+	387625	388268	1403454
80.te00116	30	chr30_30.00575	2	+	408258	408904	1403454
80.te00117	30	chr30_30.00576	2	-	422220	422840	1403454
80.te00118	30	chr30_30.00577	2	+	447619	448212	1403454
80.te00119	30	chr30_30.00578	2	+	481572	482076	1403454
80.te00127	30	chr30_30.00586	2	+	517070	517587	1403454
80.te00128	30	chr30_30.00587	2	+	525200	525759	1403454

80.te00129	30	chr30_30.00588	2	+	526521	527079	1403454
80.te00130	30	chr30_30.00589	2	+	527841	528399	1403454
80.te00131	30	chr30_30.00590	2	+	529163	529721	1403454
80.te00132	30	chr30_30.00591	2	+	530481	531031	1403454
80.te00133	30	chr30_30.00592	2	+	532968	533520	1403454
80.te00134	30	chr30_30.00593	2	+	535455	536013	1403454
80.te00135	30	chr30_30.00594	2	+	571384	571871	1403454
80.te00137	30	chr30_30.00596	2	+	639477	640057	1403454
80.te00138	30	chr30_30.00597	2	+	659940	660496	1403454
80.te00139	30	chr30_30.00598	2	+	701564	702131	1403454
80.te00140	30	chr30_30.00599	2	+	759914	760370	1403454
80.te00141	30	chr30_30.00600	2	+	768055	768487	1403454
80.te00143	30	chr30_30.00602	2	+	780496	780859	1403454
80.te00145	30	chr30_30.00604	2	-	809796	810376	1403454
80.te00146	30	chr30_30.00605	2	-	815568	816140	1403454
80.te00148	30	chr30_30.00607	2	-	819419	819985	1403454
80.te00149	30	chr30_30.00608	2	-	826863	827362	1403454
80.te00153	30	chr30_30.00612	2	-	905240	905605	1403454
80.te00154	30	chr30_30.00613	2	+	992938	993548	1403454
80.te00070	30	chr30_30.00619	2	-	1049711	1050308	1403454
80.te00072	30	chr30_30.00620	2	-	1067868	1068468	1403454
80.te00074	30	chr30_30.00621	2	-	1111051	1111604	1403454
80.te00075	30	chr30_30.00622	2	-	1159649	1160212	1403454
80.te00078	30	chr30_30.00625	2	-	1172024	1172561	1403454
80.te00079	30	chr30_30.00626	2	-	1178291	1178853	1403454
80.te00080	30	chr30_30.00627	2	+	1192935	1193508	1403454
80.te00083	30	chr30_30.00630	2	-	1208575	1209135	1403454
80.te00084	30	chr30_30.00631	2	-	1212399	1212954	1403454
80.te00085	30	chr30_30.00632	2	+	1246028	1246584	1403454
80.te00087	30	chr30_30.00634	2	+	1280999	1281557	1403454
80.te00089	30	chr30_30.00636	2	-	1287086	1287658	1403454
80.te00096	30	chr30_30.00643	2	-	1365171	1365741	1403454
81.te00182	31	chr31_31.00605	1	-	7164	7584	1484336
81.te00202	31	chr31_31.00607	1	-	98557	99051	1484336
81.te00099	31	chr31_31.00608	1	-	100102	100551	1484336
81.te00103	31	chr31_31.00609	1	+	105469	105964	1484336
81.te00135	31	chr31_31.00611	1	-	139399	140322	1484336
81.te00137	31	chr31_31.00612	1	+	144780	145018	1484336
81.te00139	31	chr31_31.00613	1	-	149981	150469	1484336
81.te00141	31	chr31_31.00615	1	-	170035	170847	1484336
81.te00146	31	chr31_31.00619	1	-	248498	249061	1484336
81.te00147	31	chr31_31.00620	1	-	267612	267878	1484336
81.te00151	31	chr31_31.00624	1	+	313040	313897	1484336
81.te00152	31	chr31_31.00625	1	-	318346	318846	1484336
81.te00154	31	chr31_31.00627	1	-	340017	340540	1484336
81.te00155	31	chr31_31.00628	1	-	347664	348209	1484336
81.te00156	31	chr31_31.00629	1	-	348784	349307	1484336
81.te00157	31	chr31_31.00630	1	-	356444	356975	1484336
81.te00158	31	chr31_31.00631	1	-	364086	364631	1484336
81.te00160	31	chr31_31.00633	1	-	396631	397285	1484336
81.te00162	31	chr31_31.00635	1	+	420660	421165	1484336
81.te00163	31	chr31_31.00636	1	-	428778	429260	1484336

81.te00165	31	chr31_31.00638	1	-	445790	446273	1484336
81.te00167	31	chr31_31.00640	1	-	485437	485907	1484336
81.te00171	31	chr31_31.00644	1	-	570567	570841	1484336
81.te00174	31	chr31_31.00647	1	-	611580	611973	1484336
81.te00175	31	chr31_31.00648	1	-	620158	621128	1484336
81.te00176	31	chr31_31.00649	1	-	622808	623604	1484336
81.te00178	31	chr31_31.00651	1	-	636715	637686	1484336
81.te00188	31	chr31_31.00660	1	-	854311	854674	1484336
81.te00191	31	chr31_31.00663	1	-	874278	875192	1484336
81.te00197	31	chr31_31.00669	1	-	933072	933433	1484336
81.te00198	31	chr31_31.00670	1	-	937977	938767	1484336
81.te00200	31	chr31_31.00672	1	-	950488	951274	1484336
81.te00098	31	chr31_31.00676	1	-	1000283	1001164	1484336
81.te00104	31	chr31_31.00680	1	-	1061525	1062003	1484336
81.te00105	31	chr31_31.00681	1	-	1069536	1070207	1484336
81.te00106	31	chr31_31.00682	1	-	1084681	1085026	1484336
81.te00107	31	chr31_31.00683	1	-	1093785	1094440	1484336
81.te00111	31	chr31_31.00686	1	-	1124991	1125902	1484336
81.te00112	31	chr31_31.00687	1	-	1128194	1128949	1484336
81.te00114	31	chr31_31.00689	1	-	1148348	1148996	1484336
81.te00117	31	chr31_31.00692	1	-	1158333	1158595	1484336
81.te00118	31	chr31_31.00693	1	-	1163239	1163491	1484336
81.te00122	31	chr31_31.00697	1	-	1207823	1208464	1484336
81.te00127	31	chr31_31.00702	1	-	1285808	1286514	1484336
81.te00128	31	chr31_31.00703	1	-	1286627	1286997	1484336
81.te00129	31	chr31_31.00704	1	-	1293003	1293395	1484336
81.te00130	31	chr31_31.00705	1	-	1345848	1346498	1484336
81.te00131	31	chr31_31.00706	1	-	1346495	1346724	1484336
81.te00192	31	chr31_31.00664	1	-	886417	886866	1484336
81.te00143	31	chr31_31.00604	2	+	1900	2467	1484336
81.te00136	31	chr31_31.00606	2	-	13618	14201	1484336
81.te00108	31	chr31_31.00610	2	+	109839	110458	1484336
81.te00140	31	chr31_31.00614	2	-	159518	160123	1484336
81.te00142	31	chr31_31.00616	2	+	181726	182345	1484336
81.te00144	31	chr31_31.00617	2	-	204306	204904	1484336
81.te00145	31	chr31_31.00618	2	-	241840	242249	1484336
81.te00148	31	chr31_31.00621	2	-	278581	279144	1484336
81.te00149	31	chr31_31.00622	2	-	306483	307049	1484336
81.te00150	31	chr31_31.00623	2	-	310545	311133	1484336
81.te00153	31	chr31_31.00626	2	+	334232	334822	1484336
81.te00159	31	chr31_31.00632	2	-	390469	391063	1484336
81.te00161	31	chr31_31.00634	2	-	417644	418220	1484336
81.te00164	31	chr31_31.00637	2	-	436380	436976	1484336
81.te00166	31	chr31_31.00639	2	+	484879	485434	1484336
81.te00168	31	chr31_31.00641	2	+	493915	494430	1484336
81.te00169	31	chr31_31.00642	2	-	495021	495611	1484336
81.te00170	31	chr31_31.00643	2	-	523481	524082	1484336
81.te00172	31	chr31_31.00645	2	-	583047	583649	1484336
81.te00173	31	chr31_31.00646	2	-	589124	589726	1484336
81.te00177	31	chr31_31.00650	2	-	624038	624616	1484336
81.te00179	31	chr31_31.00652	2	-	648495	649089	1484336
81.te00180	31	chr31_31.00653	2	-	715927	716523	1484336

81.te00181	31	chr31_31.00654	2	-	731411	732006	1484336
81.te00183	31	chr31_31.00655	2	-	768411	768917	1484336
81.te00184	31	chr31_31.00656	2	-	780370	780849	1484336
81.te00185	31	chr31_31.00657	2	-	813313	813858	1484336
81.te00186	31	chr31_31.00658	2	-	831990	832570	1484336
81.te00187	31	chr31_31.00659	2	-	848902	849456	1484336
81.te00189	31	chr31_31.00661	2	-	859769	860123	1484336
81.te00190	31	chr31_31.00662	2	-	866416	866835	1484336
81.te00193	31	chr31_31.00665	2	+	917950	918543	1484336
81.te00194	31	chr31_31.00666	2	-	927007	927442	1484336
81.te00195	31	chr31_31.00667	2	-	930114	930388	1484336
81.te00196	31	chr31_31.00668	2	-	932051	932605	1484336
81.te00199	31	chr31_31.00671	2	+	949178	949618	1484336
81.te00201	31	chr31_31.00673	2	+	964549	965121	1484336
81.te00203	31	chr31_31.00674	2	-	990860	991176	1484336
81.te00204	31	chr31_31.00675	2	-	992937	993391	1484336
81.te00100	31	chr31_31.00677	2	-	1016349	1016944	1484336
81.te00101	31	chr31_31.00678	2	-	1033384	1033986	1484336
81.te00102	31	chr31_31.00679	2	-	1042717	1043307	1484336
81.te00109	31	chr31_31.00684	2	-	1101696	1102269	1484336
81.te00110	31	chr31_31.00685	2	-	1117444	1118022	1484336
81.te00113	31	chr31_31.00688	2	-	1137807	1138397	1484336
81.te00115	31	chr31_31.00690	2	-	1150605	1151071	1484336
81.te00116	31	chr31_31.00691	2	-	1155251	1155814	1484336
81.te00119	31	chr31_31.00694	2	-	1175820	1176424	1484336
81.te00120	31	chr31_31.00695	2	+	1188290	1188903	1484336
81.te00121	31	chr31_31.00696	2	+	1206805	1207390	1484336
81.te00123	31	chr31_31.00698	2	-	1231033	1231464	1484336
81.te00124	31	chr31_31.00699	2	-	1242579	1242935	1484336
81.te00125	31	chr31_31.00700	2	-	1251533	1251892	1484336
81.te00126	31	chr31_31.00701	2	-	1279703	1280267	1484336
81.te00132	31	chr31_31.00707	2	-	1350330	1350875	1484336
81.te00133	31	chr31_31.00708	2	+	1374149	1374882	1484336
81.te00134	31	chr31_31.00709	2	-	1382534	1383132	1484336
81.te00138	31	chr31_31.00710	2	+	1450368	1450966	1484336
82.te00087	32	chr32_32.00733	1	-	172653	173110	1565925
82.te00088	32	chr32_32.00734	1	-	179507	180309	1565925
82.te00090	32	chr32_32.00736	1	+	198285	198790	1565925
82.te00093	32	chr32_32.00739	1	+	246630	246984	1565925
82.te00094	32	chr32_32.00740	1	+	247074	247811	1565925
82.te00096	32	chr32_32.00742	1	+	272035	272436	1565925
82.te00099	32	chr32_32.00744	1	+	347962	348764	1565925
82.te00103	32	chr32_32.00747	1	+	381631	382496	1565925
82.te00104	32	chr32_32.00748	1	+	391036	391641	1565925
82.te00108	32	chr32_32.00751	1	+	451771	452773	1565925
82.te00110	32	chr32_32.00753	1	+	487217	487673	1565925
82.te00119	32	chr32_32.00762	1	-	698193	698999	1565925
82.te00128	32	chr32_32.00770	1	-	773158	774187	1565925
82.te00132	32	chr32_32.00774	1	-	817931	818960	1565925
82.te00140	32	chr32_32.00781	1	-	967258	968289	1565925
82.te00063	32	chr32_32.00784	1	-	1029132	1030169	1565925
82.te00064	32	chr32_32.00785	1	-	1030490	1030966	1565925

82.te00065	32	chr32_32.00786	1	-	1057200	1057668	1565925
82.te00072	32	chr32_32.00792	1	-	1232409	1232951	1565925
82.te00073	32	chr32_32.00793	1	-	1257930	1258504	1565925
82.te00076	32	chr32_32.00795	1	+	1317330	1317560	1565925
82.te00077	32	chr32_32.00796	1	-	1348658	1348979	1565925
82.te00079	32	chr32_32.00798	1	+	1426155	1426341	1565925
82.te00080	32	chr32_32.00799	1	+	1456576	1457297	1565925
82.te00083	32	chr32_32.00802	1	-	1554744	1555215	1565925
82.te00085	32	chr32_32.00724	2	-	16065	16633	1565925
82.te00097	32	chr32_32.00725	2	-	28339	28924	1565925
82.te00102	32	chr32_32.00726	2	+	37448	37924	1565925
82.te00106	32	chr32_32.00727	2	-	44067	44636	1565925
82.te00120	32	chr32_32.00728	2	+	70076	70677	1565925
82.te00134	32	chr32_32.00729	2	-	82354	82943	1565925
82.te00070	32	chr32_32.00730	2	-	119109	119634	1565925
82.te00074	32	chr32_32.00731	2	-	125627	126166	1565925
82.te00086	32	chr32_32.00732	2	-	168064	168640	1565925
82.te00089	32	chr32_32.00735	2	-	184585	185157	1565925
82.te00091	32	chr32_32.00737	2	+	221304	221929	1565925
82.te00092	32	chr32_32.00738	2	+	231499	232091	1565925
82.te00095	32	chr32_32.00741	2	+	252120	252712	1565925
82.te00098	32	chr32_32.00743	2	-	308213	308799	1565925
82.te00100	32	chr32_32.00745	2	+	351853	352487	1565925
82.te00101	32	chr32_32.00746	2	+	364734	365319	1565925
82.te00105	32	chr32_32.00749	2	+	413608	414197	1565925
82.te00107	32	chr32_32.00750	2	+	448878	449449	1565925
82.te00109	32	chr32_32.00752	2	-	452977	453549	1565925
82.te00111	32	chr32_32.00754	2	+	515289	515964	1565925
82.te00112	32	chr32_32.00755	2	+	537674	538252	1565925
82.te00113	32	chr32_32.00756	2	-	541600	542251	1565925
82.te00114	32	chr32_32.00757	2	-	584082	584684	1565925
82.te00115	32	chr32_32.00758	2	-	593540	594238	1565925
82.te00116	32	chr32_32.00759	2	-	626445	626779	1565925
82.te00117	32	chr32_32.00760	2	+	638411	639049	1565925
82.te00118	32	chr32_32.00761	2	-	657323	657863	1565925
82.te00121	32	chr32_32.00763	2	-	722771	723361	1565925
82.te00122	32	chr32_32.00764	2	-	731135	731747	1565925
82.te00123	32	chr32_32.00765	2	-	749208	749805	1565925
82.te00124	32	chr32_32.00766	2	-	752382	752993	1565925
82.te00125	32	chr32_32.00767	2	-	766173	766733	1565925
82.te00126	32	chr32_32.00768	2	-	769506	769944	1565925
82.te00127	32	chr32_32.00769	2	-	772537	773135	1565925
82.te00129	32	chr32_32.00771	2	-	790521	791119	1565925
82.te00130	32	chr32_32.00772	2	-	798600	799175	1565925
82.te00131	32	chr32_32.00773	2	-	804920	805495	1565925
82.te00133	32	chr32_32.00775	2	+	824130	824733	1565925
82.te00135	32	chr32_32.00776	2	-	877869	878464	1565925
82.te00136	32	chr32_32.00777	2	-	886955	887551	1565925
82.te00137	32	chr32_32.00778	2	-	916223	916817	1565925
82.te00138	32	chr32_32.00779	2	+	934316	934967	1565925
82.te00139	32	chr32_32.00780	2	-	949020	949675	1565925
82.te00141	32	chr32_32.00782	2	-	988455	989068	1565925

82.te00062	32	chr32_32.00783	2	-	1014700	1015339	1565925
82.te00066	32	chr32_32.00787	2	-	1125921	1126488	1565925
82.te00067	32	chr32_32.00788	2	+	1167650	1168106	1565925
82.te00068	32	chr32_32.00789	2	+	1169021	1169463	1565925
82.te00069	32	chr32_32.00790	2	+	1191706	1192265	1565925
82.te00071	32	chr32_32.00791	2	-	1215593	1216214	1565925
82.te00075	32	chr32_32.00794	2	+	1278798	1279379	1565925
82.te00078	32	chr32_32.00797	2	+	1399674	1400241	1565925
82.te00081	32	chr32_32.00800	2	+	1509736	1510260	1565925
82.te00082	32	chr32_32.00801	2	+	1546420	1546813	1565925
82.te00084	32	chr32_32.00803	2	+	1558854	1559396	1565925
83.te00113	33	chr33_33.00715	1	-	23667	24574	1583673
83.te00136	33	chr33_33.00719	1	-	57677	58588	1583673
83.te00147	33	chr33_33.00720	1	-	69997	70690	1583673
83.te00106	33	chr33_33.00724	1	+	186049	186322	1583673
83.te00107	33	chr33_33.00725	1	-	190340	191566	1583673
83.te00109	33	chr33_33.00727	1	-	194588	195588	1583673
83.te00115	33	chr33_33.00732	1	-	250184	250809	1583673
83.te00122	33	chr33_33.00738	1	-	422146	422630	1583673
83.te00128	33	chr33_33.00743	1	+	483000	483335	1583673
83.te00134	33	chr33_33.00748	1	+	544297	545132	1583673
83.te00137	33	chr33_33.00750	1	-	586247	586895	1583673
83.te00138	33	chr33_33.00751	1	+	589716	589927	1583673
83.te00139	33	chr33_33.00752	1	+	592594	593436	1583673
83.te00140	33	chr33_33.00753	1	+	595396	596062	1583673
83.te00141	33	chr33_33.00754	1	+	601015	601970	1583673
83.te00142	33	chr33_33.00755	1	-	619531	619854	1583673
83.te00144	33	chr33_33.00757	1	+	645503	646481	1583673
83.te00145	33	chr33_33.00758	1	-	654479	654698	1583673
83.te00151	33	chr33_33.00763	1	+	780425	781323	1583673
83.te00162	33	chr33_33.00772	1	-	892703	893265	1583673
83.te00163	33	chr33_33.00773	1	+	909100	909834	1583673
83.te00165	33	chr33_33.00775	1	+	950152	950427	1583673
83.te00166	33	chr33_33.00776	1	+	981680	982133	1583673
83.te00167	33	chr33_33.00777	1	+	991961	992460	1583673
83.te00063	33	chr33_33.00778	1	+	1000543	1000632	1583673
83.te00064	33	chr33_33.00779	1	+	1001016	1001931	1583673
83.te00065	33	chr33_33.00780	1	+	1039963	1040856	1583673
83.te00070	33	chr33_33.00784	1	+	1081139	1081536	1583673
83.te00072	33	chr33_33.00786	1	+	1132199	1133118	1583673
83.te00075	33	chr33_33.00789	1	-	1156088	1156420	1583673
83.te00076	33	chr33_33.00790	1	+	1167044	1167516	1583673
83.te00078	33	chr33_33.00792	1	+	1211483	1212116	1583673
83.te00079	33	chr33_33.00793	1	+	1226515	1227296	1583673
83.te00080	33	chr33_33.00794	1	+	1271600	1272442	1583673
83.te00082	33	chr33_33.00796	1	+	1288045	1288456	1583673
83.te00083	33	chr33_33.00797	1	+	1288629	1289451	1583673
83.te00086	33	chr33_33.00800	1	+	1316864	1317777	1583673
83.te00089	33	chr33_33.00803	1	+	1335012	1335913	1583673
83.te00093	33	chr33_33.00807	1	+	1393184	1393404	1583673
83.te00094	33	chr33_33.00808	1	+	1410447	1411415	1583673
83.te00096	33	chr33_33.00810	1	+	1442545	1443401	1583673

83.te00097	33	chr33_33.00811	1	+	1451823	1452664	1583673
83.te00100	33	chr33_33.00814	1	+	1469602	1470024	1583673
83.te00101	33	chr33_33.00815	1	+	1470126	1470536	1583673
83.te00104	33	chr33_33.00818	1	+	1576159	1576559	1583673
83.te00117	33	chr33_33.00734	1	+	313327	313952	1583673
83.te00118	33	chr33_33.00735	1	+	332004	332660	1583673
83.te00123	33	chr33_33.00739	1	+	426046	426686	1583673
83.te00153	33	chr33_33.00765	1	+	786288	786797	1583673
83.te00154	33	chr33_33.00766	1	+	796833	797449	1583673
83.te00156	33	chr33_33.00767	1	-	827055	827618	1583673
83.te00081	33	chr33_33.00795	1	+	1283256	1283650	1583673
83.te00085	33	chr33_33.00799	1	+	1316072	1316469	1583673
83.te00087	33	chr33_33.00801	1	-	1317950	1318368	1583673
83.te00088	33	chr33_33.00802	1	+	1334220	1334617	1583673
83.te00090	33	chr33_33.00804	1	-	1336086	1336485	1583673
83.te00155	33	chr33_33.00714	2	+	8050	8655	1583673
83.te00119	33	chr33_33.00716	2	-	35816	36437	1583673
83.te00126	33	chr33_33.00717	2	-	45258	45871	1583673
83.te00129	33	chr33_33.00718	2	-	47785	48421	1583673
83.te00157	33	chr33_33.00721	2	+	82772	83397	1583673
83.te00069	33	chr33_33.00722	2	+	106017	106640	1583673
83.te00105	33	chr33_33.00723	2	-	181185	181818	1583673
83.te00108	33	chr33_33.00726	2	-	193274	193894	1583673
83.te00110	33	chr33_33.00728	2	-	206202	206828	1583673
83.te00111	33	chr33_33.00729	2	-	233547	234189	1583673
83.te00112	33	chr33_33.00730	2	-	244391	245040	1583673
83.te00114	33	chr33_33.00731	2	-	247358	247991	1583673
83.te00116	33	chr33_33.00733	2	-	263638	264259	1583673
83.te00120	33	chr33_33.00736	2	+	399836	400477	1583673
83.te00121	33	chr33_33.00737	2	+	419515	420062	1583673
83.te00124	33	chr33_33.00740	2	+	428717	429351	1583673
83.te00125	33	chr33_33.00741	2	+	440941	441548	1583673
83.te00127	33	chr33_33.00742	2	+	463763	464399	1583673
83.te00130	33	chr33_33.00744	2	+	493769	494366	1583673
83.te00131	33	chr33_33.00745	2	+	500935	501573	1583673
83.te00132	33	chr33_33.00746	2	+	519357	519958	1583673
83.te00133	33	chr33_33.00747	2	-	540511	541106	1583673
83.te00135	33	chr33_33.00749	2	+	583232	583689	1583673
83.te00143	33	chr33_33.00756	2	+	624159	624718	1583673
83.te00146	33	chr33_33.00759	2	+	658200	658752	1583673
83.te00148	33	chr33_33.00760	2	+	734567	735151	1583673
83.te00149	33	chr33_33.00761	2	+	764827	765405	1583673
83.te00150	33	chr33_33.00762	2	+	770729	771294	1583673
83.te00152	33	chr33_33.00764	2	+	781337	781902	1583673
83.te00158	33	chr33_33.00768	2	+	830010	830578	1583673
83.te00159	33	chr33_33.00769	2	+	836992	837566	1583673
83.te00160	33	chr33_33.00770	2	-	882956	883530	1583673
83.te00161	33	chr33_33.00771	2	+	885679	886268	1583673
83.te00164	33	chr33_33.00774	2	+	909848	910425	1583673
83.te00066	33	chr33_33.00781	2	+	1040894	1041510	1583673
83.te00067	33	chr33_33.00782	2	-	1043384	1043993	1583673
83.te00068	33	chr33_33.00783	2	+	1059729	1060338	1583673

83.te00071	33	chr33_33.00785	2	-	1121607	1122196	1583673
83.te00073	33	chr33_33.00787	2	+	1140982	1141591	1583673
83.te00074	33	chr33_33.00788	2	+	1144059	1144709	1583673
83.te00077	33	chr33_33.00791	2	+	1197051	1197666	1583673
83.te00084	33	chr33_33.00798	2	+	1301276	1301803	1583673
83.te00091	33	chr33_33.00805	2	+	1339431	1339964	1583673
83.te00092	33	chr33_33.00806	2	+	1376336	1376904	1583673
83.te00095	33	chr33_33.00809	2	+	1441896	1442442	1583673
83.te00098	33	chr33_33.00812	2	-	1460883	1461466	1583673
83.te00099	33	chr33_33.00813	2	+	1469018	1469588	1583673
83.te00102	33	chr33_33.00816	2	+	1487913	1488500	1583673
83.te00103	33	chr33_33.00817	2	+	1561692	1562298	1583673
84.te00104	34	chr34_34.00758	1	+	55971	56279	1866754
84.te00155	34	chr34_34.00759	1	+	90809	91046	1866754
84.te00003	34	chr34_34.00760	1	+	104178	104618	1866754
84.te00030	34	chr34_34.00763	1	+	133400	133855	1866754
84.te00043	34	chr34_34.00764	1	+	144030	144227	1866754
84.te00068	34	chr34_34.00766	1	+	192894	193093	1866754
84.te00069	34	chr34_34.00767	1	+	201816	202394	1866754
84.te00070	34	chr34_34.00768	1	+	202464	202939	1866754
84.te00071	34	chr34_34.00769	1	+	220272	221220	1866754
84.te00076	34	chr34_34.00772	1	+	282076	282451	1866754
84.te00079	34	chr34_34.00774	1	+	321626	321945	1866754
84.te00080	34	chr34_34.00775	1	-	321853	322425	1866754
84.te00085	34	chr34_34.00780	1	+	372116	373114	1866754
84.te00090	34	chr34_34.00784	1	+	416853	417441	1866754
84.te00091	34	chr34_34.00785	1	+	417442	417951	1866754
84.te00093	34	chr34_34.00787	1	+	430948	431668	1866754
84.te00096	34	chr34_34.00790	1	+	470958	471526	1866754
84.te00099	34	chr34_34.00792	1	-	481535	482313	1866754
84.te00100	34	chr34_34.00793	1	-	482324	482680	1866754
84.te00101	34	chr34_34.00794	1	-	522357	522723	1866754
84.te00107	34	chr34_34.00799	1	-	653290	653951	1866754
84.te00110	34	chr34_34.00801	1	-	707210	707627	1866754
84.te00111	34	chr34_34.00802	1	+	710541	711084	1866754
84.te00112	34	chr34_34.00803	1	-	714783	715177	1866754
84.te00113	34	chr34_34.00804	1	+	718095	718638	1866754
84.te00114	34	chr34_34.00805	1	-	722325	722719	1866754
84.te00115	34	chr34_34.00806	1	+	725631	726174	1866754
84.te00116	34	chr34_34.00807	1	-	729602	729996	1866754
84.te00117	34	chr34_34.00808	1	+	732901	733444	1866754
84.te00118	34	chr34_34.00809	1	-	737133	737527	1866754
84.te00119	34	chr34_34.00810	1	+	740501	741044	1866754
84.te00120	34	chr34_34.00811	1	-	744731	745125	1866754
84.te00121	34	chr34_34.00812	1	+	748030	748573	1866754
84.te00122	34	chr34_34.00813	1	-	752270	752664	1866754
84.te00123	34	chr34_34.00814	1	+	755569	756112	1866754
84.te00124	34	chr34_34.00815	1	-	759797	760191	1866754
84.te00125	34	chr34_34.00816	1	+	763108	763651	1866754
84.te00126	34	chr34_34.00817	1	-	767340	767734	1866754
84.te00127	34	chr34_34.00818	1	+	770650	771193	1866754
84.te00128	34	chr34_34.00819	1	-	774888	775282	1866754

84.te00129	34	chr34_34.00820	1	+	778184	778727	1866754
84.te00130	34	chr34_34.00821	1	-	782411	782805	1866754
84.te00131	34	chr34_34.00822	1	+	785722	786265	1866754
84.te00132	34	chr34_34.00823	1	-	789964	790358	1866754
84.te00133	34	chr34_34.00824	1	+	793275	793818	1866754
84.te00134	34	chr34_34.00825	1	-	797517	797911	1866754
84.te00135	34	chr34_34.00826	1	+	800817	801360	1866754
84.te00136	34	chr34_34.00827	1	-	805047	805441	1866754
84.te00137	34	chr34_34.00828	1	+	808356	808899	1866754
84.te00138	34	chr34_34.00829	1	-	812594	812988	1866754
84.te00139	34	chr34_34.00830	1	+	815910	816453	1866754
84.te00140	34	chr34_34.00831	1	-	819881	820275	1866754
84.te00141	34	chr34_34.00832	1	+	823179	823722	1866754
84.te00142	34	chr34_34.00833	1	-	827419	827813	1866754
84.te00143	34	chr34_34.00834	1	+	830731	831274	1866754
84.te00144	34	chr34_34.00835	1	-	834957	835351	1866754
84.te00145	34	chr34_34.00836	1	+	838265	838808	1866754
84.te00146	34	chr34_34.00837	1	-	842504	842898	1866754
84.te00147	34	chr34_34.00838	1	+	845811	846354	1866754
84.te00148	34	chr34_34.00839	1	-	850049	850443	1866754
84.te00149	34	chr34_34.00840	1	+	853349	853892	1866754
84.te00150	34	chr34_34.00841	1	-	857581	857975	1866754
84.te00151	34	chr34_34.00842	1	+	860880	861423	1866754
84.te00152	34	chr34_34.00843	1	-	865123	865517	1866754
84.te00153	34	chr34_34.00844	1	+	868421	868964	1866754
84.te00156	34	chr34_34.00846	1	-	913162	913424	1866754
84.te00157	34	chr34_34.00847	1	-	915551	915879	1866754
84.te00158	34	chr34_34.00848	1	-	933680	934762	1866754
84.te00159	34	chr34_34.00849	1	-	935344	935645	1866754
84.te00160	34	chr34_34.00850	1	-	947466	948552	1866754
84.te00162	34	chr34_34.00852	1	-	971630	972700	1866754
84.te00163	34	chr34_34.00853	1	-	973282	973582	1866754
84.te00004	34	chr34_34.00857	1	-	1049869	1050884	1866754
84.te00005	34	chr34_34.00858	1	-	1057122	1058133	1866754
84.te00009	34	chr34_34.00861	1	-	1085732	1086735	1866754
84.te00010	34	chr34_34.00862	1	-	1086861	1087034	1866754
84.te00016	34	chr34_34.00866	1	+	1197887	1198857	1866754
84.te00018	34	chr34_34.00868	1	+	1223511	1223963	1866754
84.te00021	34	chr34_34.00871	1	+	1267819	1268773	1866754
84.te00022	34	chr34_34.00872	1	-	1268425	1268674	1866754
84.te00024	34	chr34_34.00874	1	+	1285931	1286448	1866754
84.te00025	34	chr34_34.00875	1	+	1287002	1287823	1866754
84.te00032	34	chr34_34.00881	1	+	1373904	1374247	1866754
84.te00033	34	chr34_34.00882	1	+	1374657	1374961	1866754
84.te00035	34	chr34_34.00884	1	+	1393774	1394142	1866754
84.te00036	34	chr34_34.00885	1	+	1394529	1394880	1866754
84.te00040	34	chr34_34.00889	1	+	1416545	1417202	1866754
84.te00045	34	chr34_34.00892	1	+	1442454	1442900	1866754
84.te00050	34	chr34_34.00897	1	+	1498691	1499134	1866754
84.te00054	34	chr34_34.00901	1	+	1562963	1563217	1866754
84.te00056	34	chr34_34.00903	1	+	1646293	1647044	1866754
84.te00061	34	chr34_34.00908	1	-	1721906	1722246	1866754

84.te00062	34	chr34_34.00909	1	+	1731308	1731660	1866754
84.te00066	34	chr34_34.00913	1	-	1854318	1855365	1866754
84.te00073	34	chr34_34.00755	1	+	24267	24665	1866754
84.te00098	34	chr34_34.00757	1	+	47449	47801	1866754
84.te00037	34	chr34_34.00886	1	+	1402349	1402892	1866754
84.te00038	34	chr34_34.00887	1	+	1405074	1405555	1866754
84.te00039	34	chr34_34.00888	1	+	1411244	1411741	1866754
84.te00041	34	chr34_34.00890	1	+	1419291	1419806	1866754
84.te00042	34	chr34_34.00891	1	+	1430203	1430732	1866754
84.te00055	34	chr34_34.00902	1	+	1586323	1587117	1866754
84.te00067	34	chr34_34.00914	1	-	1860957	1861562	1866754
84.te00074	34	chr34_34.00751	2	-	2064	2448	1866754
84.te00078	34	chr34_34.00752	2	+	3044	3434	1866754
84.te00109	34	chr34_34.00753	2	-	6458	6862	1866754
84.te00012	34	chr34_34.00754	2	-	11089	11484	1866754
84.te00087	34	chr34_34.00756	2	+	39211	39615	1866754
84.te00007	34	chr34_34.00761	2	+	106531	107142	1866754
84.te00014	34	chr34_34.00762	2	+	118062	118669	1866754
84.te00044	34	chr34_34.00765	2	-	143700	144184	1866754
84.te00072	34	chr34_34.00770	2	+	230485	231032	1866754
84.te00075	34	chr34_34.00771	2	+	255488	256139	1866754
84.te00077	34	chr34_34.00773	2	+	302256	302700	1866754
84.te00081	34	chr34_34.00776	2	+	338072	338669	1866754
84.te00082	34	chr34_34.00777	2	+	347655	348260	1866754
84.te00083	34	chr34_34.00778	2	+	352801	353284	1866754
84.te00084	34	chr34_34.00779	2	+	363078	363681	1866754
84.te00086	34	chr34_34.00781	2	+	388128	388715	1866754
84.te00088	34	chr34_34.00782	2	+	392999	393603	1866754
84.te00089	34	chr34_34.00783	2	+	407135	407749	1866754
84.te00092	34	chr34_34.00786	2	+	428548	429165	1866754
84.te00094	34	chr34_34.00788	2	+	449632	449790	1866754
84.te00095	34	chr34_34.00789	2	-	465689	466327	1866754
84.te00097	34	chr34_34.00791	2	-	471527	471713	1866754
84.te00102	34	chr34_34.00795	2	-	526682	527298	1866754
84.te00103	34	chr34_34.00796	2	-	531101	531717	1866754
84.te00105	34	chr34_34.00797	2	+	561431	561674	1866754
84.te00106	34	chr34_34.00798	2	-	585979	586586	1866754
84.te00108	34	chr34_34.00800	2	-	679632	680131	1866754
84.te00154	34	chr34_34.00845	2	+	878064	878674	1866754
84.te00161	34	chr34_34.00851	2	-	968827	969337	1866754
84.te00164	34	chr34_34.00854	2	-	982290	982880	1866754
84.te00001	34	chr34_34.00855	2	-	1019065	1019680	1866754
84.te00002	34	chr34_34.00856	2	-	1039476	1039945	1866754
84.te00006	34	chr34_34.00859	2	-	1062822	1063426	1866754
84.te00008	34	chr34_34.00860	2	+	1083887	1084522	1866754
84.te00011	34	chr34_34.00863	2	-	1099333	1099983	1866754
84.te00013	34	chr34_34.00864	2	-	1154822	1155395	1866754
84.te00015	34	chr34_34.00865	2	+	1194518	1194749	1866754
84.te00017	34	chr34_34.00867	2	-	1218996	1219623	1866754
84.te00019	34	chr34_34.00869	2	+	1257585	1258138	1866754
84.te00020	34	chr34_34.00870	2	+	1265188	1265807	1866754
84.te00023	34	chr34_34.00873	2	+	1270834	1271394	1866754

84.te00026	34	chr34_34.00876	2	+	1298994	1299585	1866754
84.te00027	34	chr34_34.00877	2	+	1320576	1321166	1866754
84.te00028	34	chr34_34.00878	2	+	1324603	1325187	1866754
84.te00029	34	chr34_34.00879	2	+	1330244	1330854	1866754
84.te00031	34	chr34_34.00880	2	+	1368275	1368887	1866754
84.te00034	34	chr34_34.00883	2	+	1393192	1393757	1866754
84.te00046	34	chr34_34.00893	2	+	1446686	1447210	1866754
84.te00047	34	chr34_34.00894	2	+	1458859	1459482	1866754
84.te00048	34	chr34_34.00895	2	+	1482881	1483546	1866754
84.te00049	34	chr34_34.00896	2	+	1492706	1492993	1866754
84.te00051	34	chr34_34.00898	2	+	1506228	1506757	1866754
84.te00052	34	chr34_34.00899	2	+	1506294	1506499	1866754
84.te00053	34	chr34_34.00900	2	+	1560622	1561204	1866754
84.te00057	34	chr34_34.00904	2	+	1665561	1666149	1866754
84.te00058	34	chr34_34.00905	2	+	1676807	1677354	1866754
84.te00059	34	chr34_34.00906	2	+	1696500	1697120	1866754
84.te00060	34	chr34_34.00907	2	-	1699826	1700447	1866754
84.te00063	34	chr34_34.00910	2	-	1740021	1740622	1866754
84.te00064	34	chr34_34.00911	2	+	1799891	1800508	1866754
84.te00065	34	chr34_34.00912	2	+	1802315	1802925	1866754
85.te00225	35	chr35_35.01068	1	-	71605	72067	2090491
85.te00122	35	chr35_35.01069	1	+	104821	105257	2090491
85.te00134	35	chr35_35.01071	1	+	114205	115373	2090491
85.te00139	35	chr35_35.01072	1	+	121208	121571	2090491
85.te00140	35	chr35_35.01073	1	+	123092	123455	2090491
85.te00194	35	chr35_35.01075	1	+	305762	306405	2090491
85.te00195	35	chr35_35.01076	1	+	325881	326067	2090491
85.te00197	35	chr35_35.01078	1	+	339144	339562	2090491
85.te00198	35	chr35_35.01079	1	+	352508	353110	2090491
85.te00200	35	chr35_35.01081	1	+	382446	382743	2090491
85.te00201	35	chr35_35.01082	1	+	382851	383166	2090491
85.te00202	35	chr35_35.01083	1	+	383616	384175	2090491
85.te00206	35	chr35_35.01087	1	-	460461	461288	2090491
85.te00212	35	chr35_35.01092	1	+	521701	522069	2090491
85.te00213	35	chr35_35.01093	1	+	523876	524959	2090491
85.te00216	35	chr35_35.01096	1	-	591916	592989	2090491
85.te00222	35	chr35_35.01101	1	+	657967	659043	2090491
85.te00230	35	chr35_35.01108	1	-	756464	756499	2090491
85.te00231	35	chr35_35.01109	1	+	764377	764514	2090491
85.te00232	35	chr35_35.01110	1	+	838663	839068	2090491
85.te00235	35	chr35_35.01113	1	-	864337	864729	2090491
85.te00237	35	chr35_35.01115	1	+	924717	925939	2090491
85.te00240	35	chr35_35.01118	1	+	981008	982083	2090491
85.te00125	35	chr35_35.01122	1	-	1071380	1072199	2090491
85.te00127	35	chr35_35.01124	1	-	1091176	1091689	2090491
85.te00129	35	chr35_35.01125	1	+	1105881	1106164	2090491
85.te00131	35	chr35_35.01127	1	-	1130754	1131686	2090491
85.te00133	35	chr35_35.01129	1	-	1136734	1137580	2090491
85.te00136	35	chr35_35.01131	1	-	1153181	1154055	2090491
85.te00138	35	chr35_35.01133	1	-	1191905	1192979	2090491
85.te00141	35	chr35_35.01134	1	-	1230221	1231402	2090491
85.te00142	35	chr35_35.01135	1	-	1233103	1233621	2090491

85.te00143	35	chr35_35.01136	1	-	1244370	1244822	2090491
85.te00149	35	chr35_35.01142	1	+	1406646	1407402	2090491
85.te00151	35	chr35_35.01144	1	+	1422091	1422477	2090491
85.te00152	35	chr35_35.01145	1	+	1423802	1424725	2090491
85.te00155	35	chr35_35.01148	1	-	1451702	1452618	2090491
85.te00156	35	chr35_35.01149	1	-	1453954	1454328	2090491
85.te00159	35	chr35_35.01152	1	-	1487412	1488330	2090491
85.te00162	35	chr35_35.01155	1	+	1585024	1585788	2090491
85.te00163	35	chr35_35.01156	1	+	1600926	1601844	2090491
85.te00166	35	chr35_35.01159	1	+	1706398	1707270	2090491
85.te00168	35	chr35_35.01161	1	+	1729878	1730226	2090491
85.te00169	35	chr35_35.01162	1	+	1731427	1732313	2090491
85.te00171	35	chr35_35.01164	1	-	1803448	1803723	2090491
85.te00173	35	chr35_35.01166	1	+	1829211	1829448	2090491
85.te00174	35	chr35_35.01167	1	+	1835794	1836213	2090491
85.te00181	35	chr35_35.01174	1	-	1941384	1941589	2090491
85.te00182	35	chr35_35.01175	1	-	1942891	1943641	2090491
85.te00189	35	chr35_35.01182	1	+	2029544	2029757	2090491
85.te00190	35	chr35_35.01183	1	+	2059060	2059644	2090491
85.te00191	35	chr35_35.01184	1	+	2063721	2064706	2090491
85.te00192	35	chr35_35.01185	1	-	2067597	2068343	2090491
85.te00128	35	chr35_35.01070	1	-	108750	109344	2090491
85.te00124	35	chr35_35.01065	2	-	10093	10677	2090491
85.te00207	35	chr35_35.01066	2	-	45720	46312	2090491
85.te00218	35	chr35_35.01067	2	+	63256	63826	2090491
85.te00193	35	chr35_35.01074	2	+	280016	280630	2090491
85.te00196	35	chr35_35.01077	2	+	335142	335776	2090491
85.te00199	35	chr35_35.01080	2	+	356557	357151	2090491
85.te00203	35	chr35_35.01084	2	+	405754	406357	2090491
85.te00204	35	chr35_35.01085	2	+	436081	436649	2090491
85.te00205	35	chr35_35.01086	2	-	447552	448139	2090491
85.te00208	35	chr35_35.01088	2	+	471027	471568	2090491
85.te00209	35	chr35_35.01089	2	-	491437	491794	2090491
85.te00210	35	chr35_35.01090	2	-	494371	494911	2090491
85.te00211	35	chr35_35.01091	2	-	516288	516763	2090491
85.te00214	35	chr35_35.01094	2	+	563403	563964	2090491
85.te00215	35	chr35_35.01095	2	-	571776	572334	2090491
85.te00217	35	chr35_35.01097	2	+	613989	614582	2090491
85.te00219	35	chr35_35.01098	2	+	635840	636326	2090491
85.te00220	35	chr35_35.01099	2	-	641647	642182	2090491
85.te00221	35	chr35_35.01100	2	-	648249	648745	2090491
85.te00223	35	chr35_35.01102	2	-	678044	678594	2090491
85.te00224	35	chr35_35.01103	2	-	707571	708125	2090491
85.te00226	35	chr35_35.01104	2	-	722665	723251	2090491
85.te00227	35	chr35_35.01105	2	-	728915	729467	2090491
85.te00228	35	chr35_35.01106	2	+	743252	743823	2090491
85.te00229	35	chr35_35.01107	2	+	754567	755126	2090491
85.te00233	35	chr35_35.01111	2	+	849692	850272	2090491
85.te00234	35	chr35_35.01112	2	+	857014	857596	2090491
85.te00236	35	chr35_35.01114	2	+	877911	878452	2090491
85.te00238	35	chr35_35.01116	2	+	952513	953126	2090491
85.te00239	35	chr35_35.01117	2	-	980557	980756	2090491

85.te00241	35	chr35_35.01119	2	-	982304	982508	2090491
85.te00242	35	chr35_35.01120	2	+	997635	998263	2090491
85.te00123	35	chr35_35.01121	2	+	1053343	1053939	2090491
85.te00126	35	chr35_35.01123	2	+	1087483	1087922	2090491
85.te00130	35	chr35_35.01126	2	-	1110526	1111098	2090491
85.te00132	35	chr35_35.01128	2	-	1133109	1133666	2090491
85.te00135	35	chr35_35.01130	2	-	1148999	1149506	2090491
85.te00137	35	chr35_35.01132	2	-	1191163	1191738	2090491
85.te00144	35	chr35_35.01137	2	-	1326412	1327034	2090491
85.te00145	35	chr35_35.01138	2	+	1356587	1357192	2090491
85.te00146	35	chr35_35.01139	2	-	1362508	1363088	2090491
85.te00147	35	chr35_35.01140	2	-	1396961	1397505	2090491
85.te00148	35	chr35_35.01141	2	+	1405035	1405562	2090491
85.te00150	35	chr35_35.01143	2	-	1407405	1407922	2090491
85.te00153	35	chr35_35.01146	2	-	1445402	1445848	2090491
85.te00154	35	chr35_35.01147	2	-	1451236	1451684	2090491
85.te00157	35	chr35_35.01150	2	-	1464748	1465131	2090491
85.te00158	35	chr35_35.01151	2	-	1486901	1487398	2090491
85.te00160	35	chr35_35.01153	2	-	1543191	1543737	2090491
85.te00161	35	chr35_35.01154	2	+	1566975	1567548	2090491
85.te00164	35	chr35_35.01157	2	+	1630776	1631367	2090491
85.te00165	35	chr35_35.01158	2	+	1697567	1698137	2090491
85.te00167	35	chr35_35.01160	2	-	1713355	1713934	2090491
85.te00170	35	chr35_35.01163	2	+	1788561	1789078	2090491
85.te00172	35	chr35_35.01165	2	+	1811470	1812125	2090491
85.te00175	35	chr35_35.01168	2	+	1838713	1839355	2090491
85.te00176	35	chr35_35.01169	2	+	1851097	1851728	2090491
85.te00177	35	chr35_35.01170	2	-	1872506	1873128	2090491
85.te00178	35	chr35_35.01171	2	+	1884803	1885309	2090491
85.te00179	35	chr35_35.01172	2	+	1913179	1913756	2090491
85.te00180	35	chr35_35.01173	2	+	1921566	1922153	2090491
85.te00183	35	chr35_35.01176	2	+	1961042	1961715	2090491
85.te00184	35	chr35_35.01177	2	+	1976295	1976956	2090491
85.te00185	35	chr35_35.01178	2	+	1983441	1984102	2090491
85.te00186	35	chr35_35.01179	2	+	1987446	1988102	2090491
85.te00187	35	chr35_35.01180	2	+	2019641	2020201	2090491
85.te00188	35	chr35_35.01181	2	+	2022219	2022763	2090491
86.te00152	36	chr36_36.01003	1	-	12395	13274	2682183
86.te00236	36	chr36_36.01004	1	-	26811	27793	2682183
86.te00280	36	chr36_36.01008	1	+	86672	86916	2682183
86.te00172	36	chr36_36.01010	1	+	176043	176981	2682183
86.te00186	36	chr36_36.01011	1	+	193247	193627	2682183
86.te00188	36	chr36_36.01012	1	+	194151	194837	2682183
86.te00201	36	chr36_36.01014	1	+	215543	216043	2682183
86.te00202	36	chr36_36.01015	1	+	216449	217201	2682183
86.te00211	36	chr36_36.01016	1	+	232529	233024	2682183
86.te00212	36	chr36_36.01017	1	+	233421	234217	2682183
86.te00234	36	chr36_36.01018	1	+	263384	264060	2682183
86.te00238	36	chr36_36.01020	1	+	305914	306639	2682183
86.te00241	36	chr36_36.01023	1	+	319281	319480	2682183
86.te00243	36	chr36_36.01025	1	+	348168	348443	2682183
86.te00245	36	chr36_36.01027	1	+	379139	380224	2682183

86.te00247	36	chr36_36.01029	1	+	431268	432053	2682183
86.te00250	36	chr36_36.01032	1	-	464838	465830	2682183
86.te00251	36	chr36_36.01033	1	-	466887	467493	2682183
86.te00252	36	chr36_36.01034	1	+	470062	470798	2682183
86.te00257	36	chr36_36.01038	1	+	502904	503071	2682183
86.te00258	36	chr36_36.01039	1	+	511133	511968	2682183
86.te00260	36	chr36_36.01041	1	-	569059	569989	2682183
86.te00262	36	chr36_36.01043	1	-	578306	579142	2682183
86.te00266	36	chr36_36.01047	1	-	596158	597225	2682183
86.te00274	36	chr36_36.01053	1	-	811633	812079	2682183
86.te00276	36	chr36_36.01055	1	+	830000	830367	2682183
86.te00277	36	chr36_36.01056	1	+	837352	837727	2682183
86.te00278	36	chr36_36.01057	1	-	843069	843335	2682183
86.te00279	36	chr36_36.01058	1	+	856877	857890	2682183
86.te00281	36	chr36_36.01059	1	+	888817	889578	2682183
86.te00285	36	chr36_36.01063	1	+	962537	962990	2682183
86.te00287	36	chr36_36.01065	1	-	994711	995064	2682183
86.te00137	36	chr36_36.01067	1	-	1005654	1006107	2682183
86.te00138	36	chr36_36.01068	1	+	1024673	1025122	2682183
86.te00141	36	chr36_36.01071	1	-	1055945	1056139	2682183
86.te00144	36	chr36_36.01074	1	+	1112180	1112417	2682183
86.te00149	36	chr36_36.01078	1	+	1174750	1175209	2682183
86.te00150	36	chr36_36.01079	1	+	1180863	1181287	2682183
86.te00153	36	chr36_36.01081	1	-	1345807	1346652	2682183
86.te00154	36	chr36_36.01082	1	-	1389081	1389346	2682183
86.te00158	36	chr36_36.01086	1	+	1505837	1506678	2682183
86.te00161	36	chr36_36.01089	1	+	1551899	1552072	2682183
86.te00162	36	chr36_36.01090	1	-	1553865	1554064	2682183
86.te00163	36	chr36_36.01091	1	+	1561262	1561666	2682183
86.te00165	36	chr36_36.01093	1	+	1577981	1578294	2682183
86.te00168	36	chr36_36.01096	1	+	1635302	1636327	2682183
86.te00169	36	chr36_36.01097	1	+	1656045	1657066	2682183
86.te00171	36	chr36_36.01099	1	+	1754626	1755366	2682183
86.te00174	36	chr36_36.01101	1	+	1765683	1766330	2682183
86.te00177	36	chr36_36.01104	1	-	1790323	1790544	2682183
86.te00178	36	chr36_36.01105	1	+	1819240	1819887	2682183
86.te00179	36	chr36_36.01106	1	+	1822135	1822780	2682183
86.te00180	36	chr36_36.01107	1	-	1838674	1838849	2682183
86.te00181	36	chr36_36.01108	1	+	1851847	1852392	2682183
86.te00183	36	chr36_36.01110	1	-	1906340	1907089	2682183
86.te00185	36	chr36_36.01112	1	-	1928183	1928929	2682183
86.te00190	36	chr36_36.01115	1	-	1994222	1994918	2682183
86.te00192	36	chr36_36.01117	1	-	2048082	2048621	2682183
86.te00193	36	chr36_36.01118	1	-	2079273	2079969	2682183
86.te00195	36	chr36_36.01120	1	-	2085860	2086538	2682183
86.te00197	36	chr36_36.01121	1	-	2111940	2112595	2682183
86.te00199	36	chr36_36.01123	1	-	2135672	2136324	2682183
86.te00205	36	chr36_36.01127	1	-	2188998	2189927	2682183
86.te00208	36	chr36_36.01130	1	-	2268878	2269941	2682183
86.te00209	36	chr36_36.01131	1	-	2284899	2285381	2682183
86.te00215	36	chr36_36.01135	1	-	2402075	2403247	2682183
86.te00216	36	chr36_36.01136	1	+	2439039	2440141	2682183

86.te00220	36	chr36_36.01140	1	-	2507189	2507446	2682183
86.te00221	36	chr36_36.01141	1	-	2507577	2508056	2682183
86.te00222	36	chr36_36.01142	1	-	2513813	2514276	2682183
86.te00223	36	chr36_36.01143	1	+	2538005	2538271	2682183
86.te00224	36	chr36_36.01144	1	+	2538282	2538950	2682183
86.te00226	36	chr36_36.01146	1	+	2574302	2574760	2682183
86.te00230	36	chr36_36.01150	1	+	2596544	2596810	2682183
86.te00231	36	chr36_36.01151	1	+	2596827	2597506	2682183
86.te00284	36	chr36_36.01062	1	+	948549	949068	2682183
86.te00254	36	chr36_36.01005	2	-	47630	48236	2682183
86.te00268	36	chr36_36.01006	2	-	68903	69488	2682183
86.te00272	36	chr36_36.01007	2	-	77582	78171	2682183
86.te00148	36	chr36_36.01009	2	+	116395	116974	2682183
86.te00196	36	chr36_36.01013	2	+	208842	209435	2682183
86.te00237	36	chr36_36.01019	2	+	304763	305123	2682183
86.te00239	36	chr36_36.01021	2	+	309270	309594	2682183
86.te00240	36	chr36_36.01022	2	-	318998	319280	2682183
86.te00242	36	chr36_36.01024	2	+	346529	347020	2682183
86.te00244	36	chr36_36.01026	2	+	352423	352961	2682183
86.te00246	36	chr36_36.01028	2	+	405242	405897	2682183
86.te00248	36	chr36_36.01030	2	+	438741	439264	2682183
86.te00249	36	chr36_36.01031	2	+	453354	453840	2682183
86.te00253	36	chr36_36.01035	2	+	478877	479400	2682183
86.te00255	36	chr36_36.01036	2	-	490656	491134	2682183
86.te00256	36	chr36_36.01037	2	+	493309	493786	2682183
86.te00259	36	chr36_36.01040	2	-	563308	563845	2682183
86.te00261	36	chr36_36.01042	2	-	571000	571556	2682183
86.te00263	36	chr36_36.01044	2	-	583578	584096	2682183
86.te00264	36	chr36_36.01045	2	-	585810	586346	2682183
86.te00265	36	chr36_36.01046	2	+	590522	591039	2682183
86.te00267	36	chr36_36.01048	2	-	630144	630654	2682183
86.te00269	36	chr36_36.01049	2	-	724675	725074	2682183
86.te00270	36	chr36_36.01050	2	-	768107	768667	2682183
86.te00271	36	chr36_36.01051	2	-	769756	770276	2682183
86.te00273	36	chr36_36.01052	2	-	787982	788426	2682183
86.te00275	36	chr36_36.01054	2	+	824346	824912	2682183
86.te00282	36	chr36_36.01060	2	+	911302	911903	2682183
86.te00283	36	chr36_36.01061	2	+	933318	933906	2682183
86.te00286	36	chr36_36.01064	2	+	983348	983959	2682183
86.te00288	36	chr36_36.01066	2	+	997474	998038	2682183
86.te00139	36	chr36_36.01069	2	+	1026030	1026581	2682183
86.te00140	36	chr36_36.01070	2	-	1038392	1038933	2682183
86.te00142	36	chr36_36.01072	2	-	1084645	1085044	2682183
86.te00143	36	chr36_36.01073	2	-	1110464	1110866	2682183
86.te00145	36	chr36_36.01075	2	-	1121789	1122202	2682183
86.te00146	36	chr36_36.01076	2	-	1130476	1130884	2682183
86.te00147	36	chr36_36.01077	2	-	1154355	1154778	2682183
86.te00151	36	chr36_36.01080	2	-	1301316	1301897	2682183
86.te00155	36	chr36_36.01083	2	-	1404297	1404876	2682183
86.te00156	36	chr36_36.01084	2	+	1409822	1410395	2682183
86.te00157	36	chr36_36.01085	2	+	1460723	1461333	2682183
86.te00159	36	chr36_36.01087	2	-	1513973	1514565	2682183

86.te00160	36	chr36_36.01088	2	+	1529778	1530361	2682183
86.te00164	36	chr36_36.01092	2	+	1573130	1573329	2682183
86.te00166	36	chr36_36.01094	2	+	1604513	1605071	2682183
86.te00167	36	chr36_36.01095	2	-	1627695	1628224	2682183
86.te00170	36	chr36_36.01098	2	+	1671431	1671994	2682183
86.te00173	36	chr36_36.01100	2	+	1764346	1764960	2682183
86.te00175	36	chr36_36.01102	2	+	1771696	1772335	2682183
86.te00176	36	chr36_36.01103	2	+	1789862	1790129	2682183
86.te00182	36	chr36_36.01109	2	+	1885590	1886168	2682183
86.te00184	36	chr36_36.01111	2	+	1907674	1908283	2682183
86.te00187	36	chr36_36.01113	2	-	1938645	1939198	2682183
86.te00189	36	chr36_36.01114	2	-	1981246	1981818	2682183
86.te00191	36	chr36_36.01116	2	-	2010807	2011317	2682183
86.te00194	36	chr36_36.01119	2	-	2081903	2082493	2682183
86.te00198	36	chr36_36.01122	2	-	2132585	2133219	2682183
86.te00200	36	chr36_36.01124	2	-	2138067	2138445	2682183
86.te00203	36	chr36_36.01125	2	+	2175021	2175274	2682183
86.te00204	36	chr36_36.01126	2	+	2182614	2182915	2682183
86.te00206	36	chr36_36.01128	2	-	2246356	2246916	2682183
86.te00207	36	chr36_36.01129	2	+	2265105	2265688	2682183
86.te00210	36	chr36_36.01132	2	-	2292375	2292864	2682183
86.te00213	36	chr36_36.01133	2	-	2346633	2347201	2682183
86.te00214	36	chr36_36.01134	2	-	2394622	2395109	2682183
86.te00217	36	chr36_36.01137	2	-	2442201	2442731	2682183
86.te00218	36	chr36_36.01138	2	-	2471638	2472088	2682183
86.te00219	36	chr36_36.01139	2	-	2496551	2497046	2682183
86.te00225	36	chr36_36.01145	2	-	2571585	2572178	2682183
86.te00227	36	chr36_36.01147	2	-	2577836	2578366	2682183
86.te00228	36	chr36_36.01148	2	-	2585844	2586424	2682183
86.te00229	36	chr36_36.01149	2	-	2595361	2595982	2682183
86.te00232	36	chr36_36.01152	2	-	2602295	2602922	2682183
86.te00233	36	chr36_36.01153	2	-	2629891	2630480	2682183
86.te00235	36	chr36_36.01154	2	-	2644826	2645451	2682183
