# Supplementary material for: Members of a Large Retroposon Family Are Determinants of Post-Transcriptional Gene Expression in Leishmania
Source: PLoS Pathog. 2007 Sep 28;3(9):e136. doi: 10.1371/journal.ppat.0030136 (PMC2323293; doi:10.1371/journal.ppat.0030136)
Supplement: Table S2 — (77 KB PDF) [file ppat.0030136.st002.pdf]

Table S2. Differential gene expression of *L. major* SIDER2-containing transcripts analyzed by DNA microarrays.

Code GeneDB		
**Promastigote-specific**[1] (26%)	**Amastigote-specific**[1] (24%)	**Constitutively expressed**[1] (50%)
LmjF21.1250	LmjF30.1500	LmjF30.0120
LmjF01.0220	*LmjF28.1400	LmjF24.1270
LmjF33.2540	LmjF19.0700	LmjF35.0570
LmjF33.2340	LmjF25.2380	LmjF25.2350
LmjF22.0690	LmjF28.0330	LmjF24.0380
*LmjF31.1890	LmjF24.1360	LmjF03.0600
*LmjF33.2550	*LmjF34.0980	LmjF18.1520
LmjF35.0970	LmjF10.0400	LmjF05.0140
LmjF05.0060	*LmjF34.0500	LmjF18.0560
LmjF23.0880		*LmjF08.1270
		LmjF33.0900
		LmjF36.2750
		LmjF35.3340
		*LmjF28.2860
		LmjF25.2280
		LmjF19.0180
		LmjF24.1260
		LmjF36.3810
		LmjF13.0440

*Confirmed by quantitative real-time RT-PCR.

[1]The data presented here are the median of four independent experiments with a standard deviation of ≤0.05. Differentially expressed mRNAs have an amastigote (A)/promastigote (P) or a P/A ratio >2.0.
