# Supplementary material for: Members of a Large Retroposon Family Are Determinants of Post-Transcriptional Gene Expression in Leishmania
Source: PLoS Pathog. 2007 Sep 28;3(9):e136. doi: 10.1371/journal.ppat.0030136 (PMC2323293; doi:10.1371/journal.ppat.0030136)
Supplement: Table S3 — (59 KB PDF) [file ppat.0030136.st003.pdf]

Table S3. Primers used for the generation of the luciferase-expressing vectors.

PCR fragment	Size (bp)	Primer sequence	Restriction site (underlined)
LUC-3'UTR3810	2371	5' GGCCGGGATCCGACGAACACA 3'	*Bam*HI
		5' AATAAGAGCGGATCCCCAAGC 3'	*Bam*HI
LUC-ΔSIDER3810	674	5' GGCCGGGATCCGACGAACACA 3'	*Bam*HI
		5' GTCTGCACTGCAGACCAAGC 3'	*Pst*I
	1047	5' CGCGTGCTGCAGTCTTGCCTTT 3'	*Pst*I
		5' AATAAGAGCGGATCCCCAAGC 3'	*Bam*HI
LUC-SIDER3810	1006	5' CGTGTGTCACGGATCCGCGCA 3'	*Bam*HI
		5' CAAGCCCGGATCCGTGTGCGG 3'	*Bam*HI
LUC-SIDER3810	634	5' GCTTGGATCCGCTGTGCAGAC 3'	*Bam*HI
		5' GGATCCAAGACAGCTGACGCG 3'	*Bam*HI
LUC-3'UTR1270	1553	5' GCGGCCGGATCCGCGCGAGCA 3'	*Bam*HI
		5' AAGCTTTGGATCCCCTTTCCCTC 3'	*Bam*HI
LUC-ΔSIDER1270	854	5' GCGGCCGGATCCGCGCGAGCA 3'	*Bam*HI
		5' AAGCTTGGATCCAGAAATGCACTG 3'	*Bam*HI
LUC-SIDER1270	699	5' GGATCCGGCCGTCTCGTGCCT 3'	*Bam*HI
		5' AAGCTTTGGATCCCCTTTCCCTC 3'	*Bam*HI
